# Effects of nutritional interventions during pregnancy on birth, child health and development outcomes: A systematic review of evidence from low‐ and middle‐income countries

**DOI:** 10.1002/cl2.1150

**Published:** 2021-06-21

**Authors:** Zohra S. Lassi, Zahra A. Padhani, Amna Rabbani, Fahad Rind, Rehana A. Salam, Zulfiqar A. Bhutta

**Affiliations:** ^1^ Robinson Research Institute University of Adelaide Adelaide South Australia SA 5005 Australia; ^2^ Center of Excellence in Women and Child Health Aga Khan University Hospital Karachi Pakistan; ^3^ Centre for Global Child Health The Hospital for Sick Children Toronto Canada

## Abstract

**Background:**

Optimal nutrition plays a crucial role in pregnancy. Poor maternal nutrition and maternal obesity has risk factors for serious fetal complications and neonatal outcomes, including intrauterine growth restriction, congenital abnormalities, stillbirth, low birth weight (LBW), preterm birth, fetal macrosomia, increased risk of neonatal infections, neonatal hypothermia, and neonatal death. The prevalence of maternal malnutrition is higher in low‐ and middle‐income countries (LMICs) (10–19%) when compared with high‐income countries, with variation by region and by country. Several behavioral interventions, including dietary control and exercise, have been found to reduce the risk of these adverse outcomes. However, none has reviewed dietary interventions to prevent maternal obesity in pregnant women.

**Objectives:**

The review aims to assess the effectiveness of balanced energy protein (BEP) supplementation, food distribution programs (FDPs), and dietary interventions to prevent maternal obesity during pregnancy on birth, child health, and developmental outcomes.

**Search Methods:**

We searched Cochrane Controlled Trials Register (CENTRAL), MEDLINE, Embase, CINAHL, and 12 other databases, and trials registers for ongoing studies up until April 2019. We also searched for gray literature from different sources and for citations on Google Scholar and Web of Sciences. We also checked the reference lists of included studies and relevant reviews and contacted the authors of studies for any ongoing and unpublished studies. The search was followed by title/abstract screening, full‐text screening and data extraction.

**Selection Criteria:**

We included randomized control trials, and quasi experimental trials to evaluate the impact of nutritional interventions (BEP, FDP, and dietary interventions to prevent maternal obesity) compared to control or standard of care, among healthy pregnant women of any age living in LMICs.

**Data Collection and Analysis:**

Two review authors independently assessed and screened studies for eligibility, extracted data, and assessed quality of the studies included in the review. We conducted a meta‐analysis of all reported primary and secondary outcomes. Subgroup analysis and GRADE assessment was performed for all reported primary outcomes.

**Main Results:**

The review included 15 studies, of these, eight were on BEP supplementation, five on FDP, and two on interventions for obesity prevention. BEP supplementation may show a reduction in the rate of stillbirths by 61% (risk ratio [RR], 0.39; 95% CI, 0.19–0.80; three studies, *n* = 1913; low quality on GRADE), perinatal mortality by 50% (RR, 0.50; 95% CI, 0.30–0.84; one study, *n* = 1446; low quality on GRADE), LBW infants by 40% (RR, 0.60; 95% CI, 0.41–0.86; three studies, *n* = 1830; low quality of evidence on GRADE); small for gestational age (SGA) by 29% (RR, 0.71; 95% CI, 0.54–0.94; five studies, *n* = 1844) and increased birth weight by 107.28 g (mean difference [MD], 107.28 g; 95% CI, 68.51–146.04, eight studies, *n* = 2190). An increase of 107.28 g of birthweight is clinically significant in the countries where the intervention was provided. BEP supplementation had no effect on miscarriage, neonatal mortality, infant mortality, preterm birth, birth length, and head circumference. FDP may show improvement in mean birth weight by 46 g (MD, 46.00 g; 95% CI, 45.10–46.90, three studies, *n* = 5272), in birth length by 0.20 cm (MD, 0.20 cm; 95% CI, 0.20–0.20, three studies, *n* = 5272), and reduction in stunting by 18% (RR, 0.82; 95% CI, 0.71–0.94; two studies; *n* = 4166), and wasting by 13% (RR, 0.87; 95% CI, 0.78–0.97; two studies, *n* = 3883). There was no effect of FDP on miscarriage, maternal mortality, perinatal mortality, neonatal mortality, infant mortality, preterm birth, LBW, SGA, head circumference, and underweight babies. Studies on interventions for obesity prevention among pregnant women failed to report on the primary outcomes. The studies showed a 195.57 g reduction in mean birth weight (MD, −195.57 g, 95% CI, −349.46 to −41.68, two studies, *n* = 180), and had no effect on birth length, and macrosomia.

**Authors' Conclusions:**

Our review highlights improvement in maternal, birth, and child outcomes through BEP supplementation and FDP during pregnancy. But, due to the small number of included studies and low quality of evidence, we are uncertain of the effect of BEP supplementation, FDP and dietary interventions for prevention of obesity on maternal, and child outcomes. Thus, further good quality research is recommended to assess the effect of these interventions on maternal, child and developmental outcomes.

## 1 PLAIN LANGUAGE SUMMARY

### 1.1 Nutrition interventions for pregnant women may improve some maternal and infant health and nutrition outcomes, but more studies are needed

Poor maternal nutrition and maternal obesity are risk factors for maternal and infant health and nutrition outcomes. Balanced energy protein (BEP) supplementation and FDPs improve some of these outcomes.

Dietary interventions to prevent maternal obesity during pregnancy can reduce birth weight with no effect on other outcomes.

#### 1.1.1 What is this review about?

Optimal nutrition plays a crucial role before, during and after pregnancy. Poor maternal nutrition and maternal obesity has risk factors for fetal complications and neonatal outcomes. Looking at birth, infant health, and developmental outcomes, this review aims to assess the effectiveness of BEP supplementation, FDPs, and dietary interventions to prevent maternal obesity during pregnancy.

**What is the aim of this review?**

This Campbell systematic review summarises the evidence from 15 studies of the effect of nutritional interventions for pregnant women on maternal and infant health outcomes.

### 1.2 What studies are included?

Eligible studies had to be randomized control trials (RCTs) or quasi‐experimental trials to evaluate the impact of nutritional interventions (BEP, FDP, and dietary interventions to prevent maternal obesity) compared to control or standard care, among healthy pregnant women of any age living in low‐ and middle‐income countries (LMICs).

Fifteen studies are included in the review. Of these, eight were on BEP supplementation, five on FDP, and two on interventions for obesity prevention. The included studies are mainly from Asia (seven studies) and Africa (six studies).

#### Do the interventions work?

Overall, BEP and FDP have a positive effect on selected maternal and infant outcomes, but not on others. Obesity prevention programs may beneficially reduce birth weight, with no effect on other outcomes. In all cases, the evidence is of low to moderate quality.

##### BEP supplementation

BEP supplementation may show a reduction in the rate of stillbirths, perinatal mortality, low birth weight (LBW), babies who are SGA, and an increase in birth weight of 107.3 g which is clinically significant in the countries where the intervention was provided.

BEP supplementation had no effect on miscarriage, neonatal mortality, infant mortality, pre‐term birth, birth length, and head circumference.

##### Food distribution programs

FDP may reduce stunting and wasting and improve mean birth weight by 46 g as well as birth length by 0.20 cm.

There was no effect of FDP on perinatal mortality, miscarriage, maternal mortality, neonatal mortality, infant mortality, preterm birth, LBW, small for genstational age, head circumference, or underweight babies.

##### Obesity prevention

Obesity prevention was associated with a 195.6 g reduction in mean birth weight but not macrosomia (the proportion of babies much larger than average for their gestational age) or birth length.

Studies on interventions for obesity prevention among pregnant women did not report other outcomes such as miscarriages and mortality.

### 1.3 **What** do the findings of this review **mean?**

Our review highlights improvement in selected maternal, birth, and infant outcomes through BEP supplementation and FDP during pregnancy, though not on others. However, due to the small number of included studies and low quality of evidence, we are uncertain of the effect of BEP supplementation, FDP and dietary interventions for prevention of obesity on maternal, and infant outcomes. Thus, further good quality research is recommended to assess the effect of these interventions on maternal, infant and developmental outcomes.

## 2 BACKGROUND

### 2.1 Description of the condition

Optimal nutrition plays a crucial role before, during, and after pregnancy (Alfaradhi & Ozanne, 2011; Black et al., 2013; Ota et al., 2015). Poor maternal nutritional status is a risk factor for serious fetal complications and the outcomes for the neonate, including intrauterine growth restriction (IUGR), stillbirth, low birth weight (LBW), preterm birth, increased risk of neonatal infections, neonatal hypothermia, and neonatal death (Ahmed et al., 2012; Black et al., 2013). Moreover, women who are undernourished at the time of conception have higher risk of obstructed labor, preeclampsia, anemia, and mortality when compared to healthy women (Christian et al., 2015; Zerfu et al., 2016).

The “Developmental Origins of Health and Disease (DOHaD)” hypothesis, previously proposed as “Fetal Origins of Adult Disease” in the 1990s (Hales et al., 1991), postulates that fetal exposure to certain environment such as exposure to a hostile uterine environment (caused by insults such as poor nutrition, infections, chemicals, metabolite, or hormonal perturbations), during critical period of development may lead to short and long term health consequences (Gluckman et al., 2008; Mandy & Nyirenda, 2018). However, if the individual then grows up in an extra‐uterine environment the reverse of that experienced in utero, therefore, would predispose them to a higher risk of certain noncommunicable diseases (Gluckman et al., 2008). This risk is further exacerbated by excessive weight gain in postnatal/adult life, and by the aging process itself. Life course theory also demonstrates the similar concept that childhood experiences affect health conditions at adulthood (e.g., diabetes, depression) (Cheng & Solomon, 2014). Likewise, antenatal malnutrition forces a fetus to adapt to an environment of scarcity and, consequently, the adverse effects extend beyond the perinatal period and end up with the child having long‐term chronic diseases such as cognitive dysfunction, obesity, diabetes mellitus, and hypertension. Conversely, evidence also suggests the harmful effects of over‐nutrition in all phases of pregnancy (Kimani‐Murage et al., 2015). Nutrition transition has engulfed developing countries, which has caused reduced mortality leading to increased populations, followed by a decrease in fertility. The increase in intake of sugar and fats has also reduced physical activity, contributing to obesity in pregnant women that lead to complicated pregnancies, which also affects the neonate at birth and in future life (Rozowski & Parodi, 2008). It was found that obesity increases the risk of fetal macrosomia, stillbirth, congenital obesity (Alfaradhi & Ozanne, 2011; Catalano & DeMouzon, 2015; Stothard et al., 2009), and infant mortality (Meehan et al., 2014).

The prevalence of maternal malnutrition is higher in LMICs when compared to high‐income countries (Black et al., 2013). Malnutrition refers to a group of nutritional disorders that include micronutrient deficiencies, under‐nutrition, and overweight/obesity. Maternal under‐nutrition is typically defined by a body‐mass index (BMI) <18.5 kg/m^2^, while overweight is classified as BMI ≥ 25 kg/m^2^ and obesity as BMI ≥ 30 kg/m^2^. The double burden of malnutrition is the co‐existence of under‐nutrition, overweight, and obesity, which has also been found to be highly prevalent in LMICs (Kimani‐Murage et al., 2015) due to diets that chronically lack diversity and infections and/or chronic disease that could contribute to deficiencies by directly inhibiting nutrient absorption.

The prevalence of maternal under‐nutrition ranges from 10% to 19% in LMICs, with variation by region and by country (Black et al., 2013). In addition, more than 10% of women aged 15–45 years living in LMICs have heights (i.e., maternal stunting defined as maternal height <145 cm) that are considerably below the average (Black et al., 2013). The prevalence of low BMI in adult women is more than 20% in Sub‐Saharan Africa and South‐Central and Southeastern Asia (Black et al., 2013). Some individual countries are worse than others. For example, in India, the prevalence of under‐nutrition among women of reproductive age reaches almost 40% (Black et al., 2013). In 2014, about 1.9 billion adult people worldwide were found to be overweight, a prevalence that surpassed that of underweight, which constituted about 462 million people. In addition, more than 600 million were reported to be obese (WHO, 2017). The prevalence of obesity is higher in the Americas and the Caribbean when compared to Africa, but overall, rates of overweight and obesity are rising globally, a situation that mimics that in high‐income countries and may be reflective of changing food environments (Black et al., 2013; WHO, 2017).

Both maternal under‐ and over‐nutrition can have adverse effects before, during, and after pregnancy (Kimani‐Murage et al., 2015). Maternal under‐nutrition throughout pregnancy has also been associated with long‐term health issues for the infant, such as obesity, diabetes mellitus, hypertension, and cognitive dysfunction (Crispi et al., 2018; Maršál, 2018). In addition, LBW has been associated with increased risk of death from coronary heart disease and stroke in adulthood (Crispi et al., 2018). Malnutrition or inadequate dietary intake during pregnancy can expose the fetus to a harsh environment, which forces the fetus to adapt. However, this adaptation can lead to permanent changes in function and structure that can later lead to chronic diseases in adult life (Crispi et al., 2018; Maršál, 2018). Maternal obesity has also been associated with higher risk of stillbirth and congenital abnormalities (Alfaradhi & Ozanne, 2011; Stothard et al., 2009). In addition, obesity during pregnancy is associated with increased risk of fetal macrosomia (Catalano & DeMouzon, 2015), which could lead to obstructed labor, and preterm birth, which is a major risk factor for infant mortality (Meehan et al., 2014). This review will focus on macronutrient supplementation during pregnancy. Micronutrient supplementation is being evaluated in a separate Campbell review of this series.

### 2.2 Description of the intervention

Several macronutrient supplementation interventions have been proposed to address maternal malnutrition especially in LMICs including balanced energy supplementation (BEP), food provision and distribution, and dietary intervention to prevent maternal obesity (Bhutta et al., 2013; Imdad & Bhutta Zulfiqar, 2012).

In LMICs, diets often lack foods rich in macronutrients and micronutrients that are typically found in meat, poultry, and fish (Gibson & Hotz, 2018). Therefore, it is important to increase the availability of macronutrients and micronutrients by promoting and introducing diverse crops, integrating farming systems with small livestock, promoting fish farming, and promoting better food storage (Gibson & Hotz, 2018). In addition, this intervention includes supplementation, which is designed to supply pregnant women in LMICs with multiple micronutrients (Allen et al., 2006; Gibson & Hotz, 2018; Zerfu et al., 2016). Such interventions have been found to be positively related to a reduced risk of maternal anemia, preterm birth, and LBW in a single study in Ethiopia (Zerfu et al., 2016).

A BEP supplement is a macronutrient food‐based supplement where proteins provide <25% of total energy content (Imdad & Bhutta Zulfiqar, 2012). BEP supplements, therefore, come in several forms. For example, a study from India provided supplements that consisted of dehusked sesame cake, jaggery, and oil containing 30 g of proteins and 417 kcal energy for undernourished pregnant women (Girija et al., 1984). In another study from Gambia, undernourished pregnant women were given daily supplements of high energy biscuits made with roasted nuts, rice flour, sugar, and groundnut oil as supplements that contained 4250 kJ energy, 22 g of proteins, 56 g fat, and vitamins, and minerals (Ceesay et al., 1997).

Two previous reviews have demonstrated the positive association of BEP interventions with pregnancy outcomes, such as reduced risk of stillbirth and small for gestational age (SGA) babies, and increase of birth weight (Imdad & Bhutta Zulfiqar, 2012; Ota et al., 2015).

Food distribution programs (FDPs) provide low‐income and undernourished pregnant and nonpregnant women and children with access to supplemental nutritious foods and often nutrition education (Baqui et al., 2008; Heaver, 2002; Kapil et al., 1992). These programs are typically run by local or international organizations. For example, India has the Integrated Nutrition and Health programme (INHP), which is a nongovernmental organization‐based program that is implemented together through CARE‐India and the Indian government (Baqui et al., 2008). This program educates pregnant women alongside the provision of healthcare services and supplementary nutrition, with the aim of increasing knowledge about maternal and newborn care, and the long‐term goal of reducing neonatal mortality (Baqui et al., 2008; Kapil, 2002). India also has the Tamil Nadu Integrated Nutrition Program (TINP), which is implemented by the state government of Tamil Nadu and supported by the World Bank. TINP aims to reduce maternal and child malnutrition through the use of a Community Nutrition Centre that makes supplementary nutrition available to pregnant women and children in villages (Heaver, 2002). In Bangladesh, the nutrition‐focused Maternal, Neonatal, and Child Health (MNCH) program supports pregnant women by providing several cross‐cutting services such as counseling on nutrition and health, micronutrient supplementation, and weight‐gain monitoring (Nguyen et al., 2017).

As noted above, obesity during pregnancy is associated with a host of maternal and fetal complications such as pre‐eclampsia, caesarian birth, macrosomia and congenital malformations (Dodd et al., 2008; Muktabhant et al., 2015). Several behavioral interventions, including dietary control, and exercise, have been found to be positively related to a reduced risk of macrosomia, cesarean delivery, and gestational weight gain (GWG; Catalano & DeMouzon, 2015; Dodd et al., 2008; Guelinckx et al., 2010; Muktabhant et al., 2015; Renault et al., 2014). Interventions can vary, and could include light to moderate‐intensity exercise, strength training, stretching, and relaxation exercises to prevent excessive weight gain (Nascimento et al., 2011) or combined dietary control and exercise interventions whereby diet counseling and advice is paired with exercise. However, in this review we will only focus on dietary interventions to prevent maternal obesity.

### 2.3 How the intervention might work

Inadequate maternal nutritional status at contraception and during pregnancy can result in adverse birth and child outcomes. Appropriate energy intake, such as BEP supplementation, and dietary education to pregnant women can lead to maternal weight gain, and increase in fetal growth (Viswanathan et al., 2008). Protein comprising 10–15% of dietary energy (Garlick & Reeds, 2000), and BEP supplementation which provides <25% of the total energy content has shown significant beneficial impact on maternal and perinatal birth outcomes such as improved birthweight (Ota et al., 2015), and reduction in stillbirths (Imdad & Bhutta Zulfiqar, 2012; Ota et al., 2015), and SGA births (Ota et al., 2015).

FDP directly provide nutritious foods or supplements to vulnerable populations, including pregnant women. There is some evidence to support the targeting of programs to pregnant women through the subsequent improvement in birth weight and reduction of infant mortality among infants of recipient mothers (Frith et al., 2015). Often, programs will provide pregnant women with healthy foods along with access to additional services, such as nutrition counseling. Counseling sessions may include information on the components of a healthy diet, the importance and consequences of poor nutrition, and food demonstrations, which provide women with the tools and knowledge necessary to maintain good antenatal health (Nguyen et al., 2017). Other interventions use community platforms, such as community health centers, to provide services such as immunization, promotion of maternal and neonatal care, and distribution of food supplements. These strategies have been shown to reduce neonatal deaths and improve maternal anemia (Baqui et al., 2008; Leroy et al., 2016).

Lifestyle interventions that include dietary control, exercise and behavioral change have been associated with a reduced risk of excessive GWG and macrosomia and decreased risk of adverse pregnancy outcomes (Catalano & DeMouzon, 2015; Dodd et al., 2008; Guelinckx et al., 2010; Muktabhant et al., 2015; Renault et al., 2014). Moreover, lifestyle interventions for maternal obesity can be implemented using a combination of dietary control and physical activity (Renault et al., 2014) or diet and exercise and behavioral change alone (Muktabhant et al., 2015; Nascimento et al., 2011). Dodd et al. (2014) used a comprehensive antenatal dietary and lifestyle counseling intervention for pregnant women in Australia. The intervention included exercise, home visits that provided dietary advice, and behavioral strategies delivered by a registered dietician (Dodd et al., 2014).

### 2.4 Why it is important to do this review

**Figure 1:**
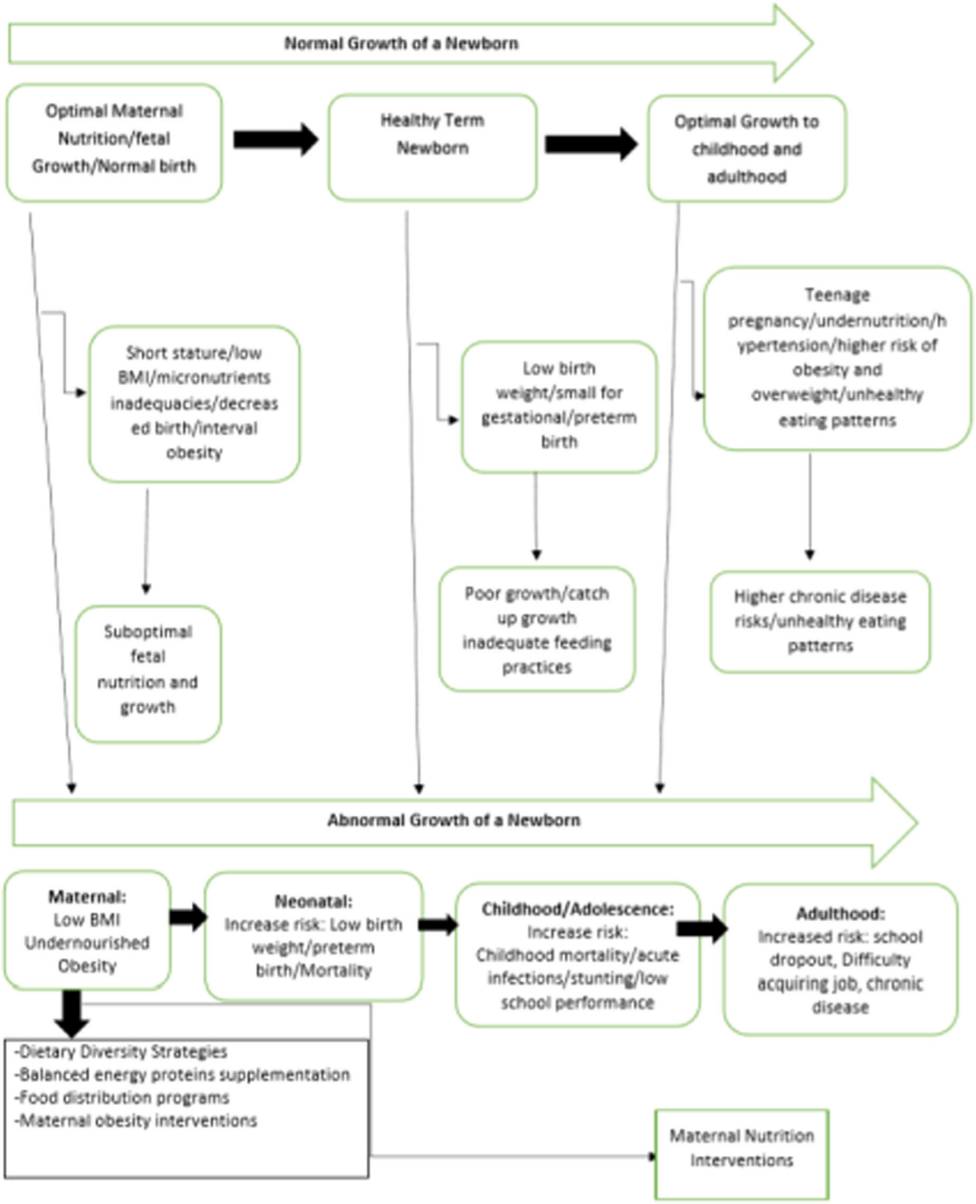
Conceptual framework for maternal nutrition interventions

Several reviews have been published that examine the impact of the interventions described above (Bhutta et al., 2013; Gibson & Hotz, 2018; Imdad & Bhutta Zulfiqar, 2012; Muktabhant et al., 2015; Ota et al., 2015; Zerfu et al., 2016). However, most of these reviews focused on the efficacy of these interventions using randomized trials and did not address the question of effectiveness of large‐scale nutrition programs. Studies of effectiveness are needed to understand whether an intervention will be impactful in a real‐world setting. Additional studies have been published recently (Devi et al., 2017; Dwarkanath et al., 2016; Huseinovic et al., 2017; Saville et al., 2018), indicating a need to update the systematic review evidence. Dietary interventions alone to prevent maternal obesity during pregnancy have not been reviewed previously. Therefore this review will make a first attempt to study its evidence. Furthermore, previous reviews did not assess the long‐term effects of these interventions during childhood. Taken together, this review will incorporate the latest evidence from RCTs and nonrandomized trials, and also assess the long‐term effects of maternal nutritional supplementation (Figure 1).

## 3 OBJECTIVES

This review aims to assess the effectiveness of nutritional interventions during pregnancy on maternal, neonatal, and childhood outcomes. The specific objectives are to assess the effectiveness of the following interventions during pregnancy on birth, child health, and developmental outcomes:

1. BEP supplementation
2. Food distribution programs
3. Dietary interventions to prevent maternal obesity

Each intervention was assessed, analysed, and reported separately.

## 4 METHODS

### 4.1 Criteria for considering studies for this review

#### 4.1.1 Types of studies

We included the following study designs:

1. Randomized controlled trials (RCTs), where participants were randomly assigned, individually or in clusters, to intervention, and comparison groups. Cross‐over designs were eligible for inclusion.
2. Quasi‐experimental designs, which include:

a. Natural experiments: studies where nonrandom assignment was determined by factors that are out of the control of the investigator. One common type includes allocation based on exogenous geographical variation.
b. Controlled before‐and‐after studies (CBA), in which measures were taken of an experimental group and a comparable control group both before and after the intervention. We also ensured that appropriate methods were used to control for confounding, such as statistical matching (e.g., propensity score matching, or covariate matching) or regression adjustment (e.g., difference‐in‐differences, instrumental variables).
c. Regression discontinuity designs; here, allocation to intervention/control was based upon a cut‐off score.
d. Interrupted time series (ITS) studies, in which outcomes were measured in the intervention group at least three time points before the intervention and after the intervention.

#### 4.1.2 Types of participants

This review includes healthy pregnant women of any age living in LMICs, as defined by the World Bank. Studies where women were recruited in the preconception period were eligible, given that women are followed throughout pregnancy. In this review, we considered women who were undernourished (inadequate nutrition) and obese women who had no other co‐morbids. We excluded all the studies conducted in high income countries. In case of multicountry studies, we planned to include data of studies conducted in LMIC only, but we did not encounter any such studies.

#### 4.1.3 Types of interventions

This review includes the following interventions that target pregnant women:

1. BEP supplementation: defined as a food supplement where proteins provide <25% of the total energy content (Imdad & Bhutta Zulfiqar, 2012).
2. FDP: FDPs are defined by their direct provision of foods to recipients, who, in this case, are pregnant women. Eligible FDPs could be locally or internationally‐led, and may or may not include elements of nutrition education.
3. Dietary interventions for prevention of maternal obesity: eligible interventions for preventing or reducing maternal obesity include dietary control and lifestyle interventions (counseling sessions) only.

Each intervention was analysed separately, and was not compared to the other interventions listed here.

##### Comparison groups

Comparison groups include standard of care (routine diet).

Each intervention was summarized separately and was not compared to each other directly.

#### 4.1.4 Types of outcome measures

This review includes studies that have the following primary and secondary maternal outcomes, fetal outcomes, newborn, and child outcomes.

##### Primary outcomes

Maternal outcomes

- Body mass index

##### Fetal and newborn outcomes

Mortality:

- Miscarriage defined as spontaneous expulsion of a human fetus before it is viable and especially between the 12th and 28th week of gestation
- Stillbirth defined as baby born with no signs of life at or after 28 weeks' gestation (WHO, 2020)
- Perinatal mortality is defined as stillbirth and deaths ≤7 days
- Neonatal mortality (death <28 days)

##### Child outcomes

- Infant mortality (deaths between 0 and 12 months)
- Under‐five mortality (deaths between 0 and 59 months)

##### Secondary outcomes

Maternal outcomes

Morbidity:

- Maternal mortality defined as the death of a woman while pregnant or within 42 days of termination of pregnancy, irrespective of cause.
- Pre‐eclampsia as defined by trial authors
- Placental abruption
- Overweight (BMI >25 and <30)
- Obesity (BMI >30)

Biochemical status:

- Anemia (hemoglobin of <10.9 g/L)
- Iron deficiency anemia

##### Fetal outcomes

Morbidity:

- Congenital anomalies

##### Newborn outcomes

Morbidity:

- LBW (<2500 g)
- Preterm birth (<37 weeks gestation)
- SGA (World Health Organization)
- Macrosomia (birth weight >4000 g)

Anthropometry:

- Birth weight (g)
- Birth Length (cm)
- Head circumference (cm)

##### Child outcomes

Morbidity:

- Stunting (<−2 *Z* score for height for age)
- Wasting (< −2 *Z* score for weight for height)
- Underweight (< −2 *Z* score for weight for age)
- Development outcomes (different scales for psychomotor development, cognitive development, attention, memory, language)
- Respiratory disease
- Allergic disease

Anemia:

- Hemoglobin concentration (g/dl)
- Iron deficiency anemia (Hb concentration of ≤10.0 or ≤10.5 g/dl) (CDC, 1998).

Studies were excluded if they have not reported the outcomes mentioned above.

###### Duration of follow up

We included all participants in eligible studies that had outcomes of interest measured. There were no restrictions based on duration of exposure, duration of follow‐up, or timing of outcome measurement. If outcomes were reported at multiple time points of follow‐up, we included outcomes based on definitions of outcomes, that is, neonates (0–28 days) versus infants (0–12 months), and so forth. Where the time of follow‐up was not clearly given, we contacted the authors for the missing information. For childhood and adulthood outcomes, we included the outcome at the longest follow‐up.

###### Type of settings

We included studies from LMIC. These countries are defined as those with a gross national income (GNI) per capita of USD 1005 or less in 2016, and lower middle‐income economies are those with a GNI per capita between USD 1006 and 3955 in 2016.

### 4.2 Search methods for identification of studies

We did not impose any restrictions on language, date, publication status, and on the literature searches described below. We also searched for any relevant retraction statements, and errata for information.

#### 4.2.1 Electronic searches

We performed searches in April 2019 on the following electronic databases:

Cochrane Controlled Trials Register (CENTRAL), MEDLINE, EMBASE, CINAHL, PsycINFO, ERIC, Sociofiles, HMIS (Health Management Information Consortium), CAB Global Health (https://www.cabi.org/publishing-products/online-information-resources/global-health/), the WHO nutrition databases (http://www.who.int/nutrition/databases/en/), Popline (https://www.popline.org), Epistemonikos (https://www.epistemonikos.org/en/), Social Science Citation Index, Dissertation Abstracts International, and WHO Global Health Index which covers the WHO Regional journals from Latin America (LILACS), Africa (AFRO), and so forth. We also searched the web sites of selected development agencies or research firms (e.g., JOLIS, IDEAS, IFPRI, NBER, USAID, World Bank, and Eldis.org). The trials registry Clinicaltrials.gov and WHO's ICRTP were searched for ongoing trials (Appendix 1).

#### 4.2.2 Searching other resources

We made every effort to contact relevant organizations and experts in the field to identify unpublished or ongoing studies. We also searched for citations at Google Scholar and Web of Sciences. References of the included articles, and relevant reviews, were scanned for eligible studies. We also searched for *gray literature on:*

- Nutrition International (NI)
- Global Alliance for Improved Nutrition (GAIN)
- World Food Programme (WFP)
- United Nations International Children's Emergency Fund (UNICEF)
- Emergency Nutrition Network (ENN)
- International Food Policy and Research Institute (IFPRI)
- WHOLIS (WHO library database)
- WHO Reproductive Health Library

We also searched the reference section of the previously published included studies and systematic reviews and contacted the authors of studies and other experts in case of any missing information.

### 4.3 Data collection and analysis

We conducted data collection and analysis in accordance with the Cochrane Handbook for Systematic Reviews of Interventions (Higgins et al., 2011).

#### 4.3.1 Criteria for determination of independent findings

Before initiating the synthesis (detailed below), we ensured that all articles reporting on the same study were appropriately linked. To ensure independence and appropriate combination of outcome constructs, syntheses were conducted according to the type of interventions specified above. If multi‐arm studies were included, intervention groups were combined or separated into different forest plots, and we ensured that there was no double counting of participants. If an outcome is reported in several different metrics, we performed unit conversions in order to pool the data. We anticipated differences in the types of literature, and therefore ensured that any analysis took possible sources of dependency into account by grouping papers into studies, and ensuring that no double counting of evidence took place when synthesizing across studies.

#### 4.3.2 Details of study coding categories

Three review authors (A. R., F. R., and Z. A. P.) independently extracted data in pairs, and a fourth review author (Z. L.) checked for reliability and resolved any conflict. We extracted the primary data for the study characteristics including details of the populations, setting, socio‐demographic characteristics, interventions, comparators, outcomes, and study design in duplicate. We checked primary study data for accuracy.

The following information was extracted for each included study:

- Background: time period when study took place, type of publication (e.g., full‐text journal article, abstract, conference paper, thesis), study country or countries,
- Population and setting: population age, and setting
- Methods: Study design, description of study arms, unit of allocation, sample or cluster size per study arm (for individually or cluster‐randomized trials respectively)
- Participants: total number randomized/allocated, socio‐demographic data
- Intervention group details: number randomized/allocated to group, description of intervention, duration and follow‐up, timing, delivery of intervention, providers and their training. We described all the study intervention arms in the tables of included studies, however, we only reported the intervention arms that met the review inclusion criteria
- Comparison group details: number randomized to group, description of comparison, duration, and follow‐up, timing, providers and their training
- Outcomes: measurement tool, validation of the tool, total number in intervention and comparison groups, change indicated at each time point
- Other information: funding source(s), and conflicts of interest.

#### 4.3.3 Selection of studies

Three review authors (Z. A. P., F. R., AR) independently screened titles and abstracts of all retrieved references in duplication. We retrieved the full‐text study reports for all citations that at least one review author considered potentially relevant. Three review authors (Z. A. P., F. R., and A. R.) independently screened the full text articles in duplication, and identified studies for inclusion, and identified and recorded reasons for exclusion of ineligible studies. We included studies irrespective of whether measured outcome data are reported in a “usable” way. We resolved any disagreement through discussion or, if required, we consulted a fourth review author (Z.L.). We identified and excluded duplicates and collated multiple reports of the same study so that each study, rather than each report, is the unit of interest in the review. We recorded the selection process in sufficient detail to complete a Preferred Reporting Items for Systematic Reviews and Meta‐Analyses (PRISMA) flow diagram.

#### 4.3.4 Data extraction and management

##### Details of study coding categories

Three authors (Z. A. P., F. R., A. R.) independently carried out data extraction in duplication using a standard data extraction form. This data extraction sheet was designed in the software Covidence and pilot tested before use. We extracted information on the following study characteristics such as, study authors, type of study, date of publication, journal, study site, participant's demographics (e.g., age, race, gender, socioeconomic status), study population, details of intervention (type, route, volume, source), outcomes, inclusion/exclusion criteria, and risk of bias (ROB). For randomized controlled trials, data was extracted on an intention to treat basis. The dichotomous outcomes were extracted as number of events in the intervention and control group. For continuous outcomes, data was extracted as mean and standard deviation (*SD*). If the mean and *SD* are not available, we converted the data by using standard methods. We also examined any published errata to assess the retraction status of the study. If a study was included with more than two intervention arms (multiarmed), we only included the arms that met the eligibility criteria.

#### 4.3.5 Assessment of ROB in included studies

Three review authors (ZAP, FR, AR) independently assessed the ROB for each included study. We resolved any disagreements by discussion or by involving a fourth review author.

For RCTs, including cluster‐RCTs, we used the Cochrane Collaboration Risk of Bias tool (Higgins et al., 2011). We assessed the ROB according to the following domains. We justified the categorical ROB/study quality judgments (e.g., high, low, and unclear) with information directly from the study.

- Random sequence generation
- Allocation concealment
- Blinding of participants and personnel
- Blinding of outcome assessment for each outcome
- Incomplete outcome data
- Selective outcome reporting
- Other bias such as the validity of outcome measure and baseline comparability

For nonrandomized controlled trials, CBA studies, and ITS, we used EPOC methods (EPOC, 2017) to assess the ROB according to the following domains. We justified the categorical ROB/study quality judgments (e.g., high, low, and unclear) with information directly from the study.

- Random sequence generation
- Allocation concealment
- Baseline outcome measurements
- Baseline characteristics similar
- Incomplete outcome data
- Knowledge of the allocated interventions adequately prevented during the study
- Protection against contamination
- Selective outcome reporting
- Other risks of bias

#### 4.3.6 Measures of treatment effect

We uploaded the outcome data for each study into the data tables in RevMan to calculate the treatment effects (RevMan, 2014). We used the risk ratio (RR) for dichotomous outcomes. We used the mean difference (MD) for continuous outcomes reported on the same scale, and the standardized mean difference (SMD) for continuous outcomes reporting the same outcome but measured on different scales. We expressed the uncertainty with 95% confidence intervals (CIs) for all effect estimates. If means and *SD*s had not been reported, we would have used other available data (e.g., CIs, *t* and *p* values) and appropriate methods described in the Cochrane Handbook for Systematic Reviews of Interventions (Higgins et al., 2011) to calculate the appropriate effect size. Where other available data were not sufficient to calculate *SD*s, we contacted the study authors. When we were unable to enter the results in either way, we described them in the table or entered the data into the “Additional tables” section. We also considered the possibility and implications of skewed data when analysing continuous outcomes as they could mislead results due to small sample size. We analysed outcomes from studies with multiple groups in an appropriate way to avoid double counting of participants by adding them to different subgroups within same plot. In such a scenario, we did not report the overall pooled estimate and only reported subgroup pooled estimate.

#### 4.3.7 Unit of analysis issues

We had a number of different outcomes and outcome subcategories. Conceptually, these subcategories could not be combined (e.g., within the cognitive development, language cannot be combined with intelligence). Therefore, a meta‐analysis was conducted separately for each outcome. Furthermore, for each outcome, we separately meta‐analysed different study designs (ITS, RCT, and CBA). We reported all the effect sizes for each outcome and did not prioritize any from others.

Where trials used clustered randomizations, we anticipated that study investigators would have presented their results after appropriately controlling for clustering effects (e.g., variance inflated standard errors, hierarchical linear models). If it was unclear whether a cluster‐randomized controlled trial had appropriately accounted for clustering, the study investigators were contacted for further information. Where appropriate controls for clustering were not used, we requested an estimate of the intra‐class correlation coefficient (ICC). Following this, effect sizes and standard errors were meta‐analysed in RevMan using the generic inverse method (Higgins et al., 2011). They were combined with estimates from individual‐level trials.

#### 4.3.8 Dealing with missing data

We contacted three authors (i.e., Abdel‐Aziz et al., 2018; Aşcı & Rathfisch, 2016; Ceesay et al., 1997) to verify key study characteristics and obtain missing numerical outcome data where possible (e.g., when we identify a study as an abstract only), but we did not receive any missing information from them. If we did not find a full report even after we contacted the study authors, we listed such an abstract as a “study awaiting classification.” If numerical outcome data were missing, such as *SD*s or correlation coefficients, and we could not obtain these from the study authors, we calculated them from other available statistics, such as *p* values, according to the methods described in the Cochrane Handbook for Systematic Reviews of Interventions (Higgins et al., 2011).

#### 4.3.9 Assessment of heterogeneity

Statistical heterogeneity was assessed using *τ* ^2^, *I* ^2^, and significance of the *χ* ^2^ test; we also assessed heterogeneity visually using forest plots. Based on prior theory and clinical knowledge, we expected clinical and methodological heterogeneity in effect sizes in this literature. Therefore, we attempted to explain any observed statistical heterogeneity using subgroup analysis.

#### 4.3.10 Assessment of reporting biases

If sufficient studies were found, funnel plots were drawn to investigate any relationship between effect size and study precision. Ten studies were usually considered sufficient to draw a funnel plot. As a direct test for publication bias, we compared the results extracted from published journal reports with results obtained from other sources (including correspondence). Whilst funnel plot asymmetry may indicate publication bias, this was not inevitably the case, and possible explanations for any asymmetry found were considered and discussed in the text of the review.

#### 4.3.11 Data synthesis

##### Synthesis procedures and statistical analysis

We prepared a matrix of all studies for each intervention which outlined all the differences in the studies, in the intervention, duration, timing, and so forth, and examined how to pool them together. Our meta‐analyses were random effects meta‐analyses, given the diverse contexts, participants, interventions, and so forth.

For each comparison, we descriptively summarized the findings from the contextual factors such as setting, timings of intervention, duration of intervention, people delivering interventions, and so forth, to assess their impact on the implementation and effectiveness of each intervention.

##### “Summary of findings” tables

We constructed “Summary of findings” tables for all of the primary outcomes using the Grading of Recommendations Assessment, Development and Evaluation (GRADE) criteria (GRADEpro GDT, 2015). These covered consideration of within‐study ROB (methodological quality), directness of evidence, heterogeneity, precision of effect estimates and risk of publication bias. We rated the certainty of evidence for each key outcome as “high,” “moderate,” “low,” or “very low.” The GRADE evidence is described in Table 1. Nonrandomized studies were initially rated as “low” quality. If there were no serious methodological flaws, we upgraded the evidence for studies with a large magnitude of effect; presence of a dose response relationship; and effect of plausible residual confounding.

**Table 1:** Quality of evidence, as determined by GRADE criteria

Quality	Description
Very low	Any estimate of effect is uncertain
Low	Further research is very likely to have important impact on our confidence in the estimate of effect and is likely to change the estimate
Moderate	Further research is likely to have an important impact on our confidence in the estimate of effect and ay change the estimate
High	Further research is very unlikely to change our confidence in the estimate of effect

We used GRADE and prepared the summary of findings tables on the following primary outcomes:

- Stillbirth defined as baby born with no signs of life at or after 28 weeks' gestation
- Perinatal mortality (stillbirth and deaths ≤7 days)
- Neonatal mortality (death <28 days)
- Infant mortality (deaths between 0 and 12 months)
- Under‐five mortality (deaths between 0 and 59 months)
- Miscarriage
- Mean maternal BMI

#### 4.3.12 Subgroup analysis and investigation of heterogeneity

We conducted the following subgroup analyses on primary outcomes when there were enough studies in each subgroup. The following subgroups would help in differentiating the impact of nutritional interventions for women based on their nutritional status, geographical location and settings, and duration of supplementation. This would aid in implementing interventions for specific population.

1) Nutritional Status: undernourished (BMI < 18.5) versus well nourished (BMI > 18.5) pregnant women defined based on BMI (for BEP and FDP)
2) Region: Africa versus South Asia versus South America and Carribean
3) Duration of supplementation: whole pregnancy versus second trimester versus third trimester
4) Nutritional Status: normal weight versus overweight versus obese (for interventions on maternal obesity)
5) Location: rural versus urban versus mixed

The subgroup analyses were conducted using Review Manager 5.3 with a test for interaction. We used *χ* ^2^ statistical tests to assess subgroup differences. *p* values of <.1 were considered significant for heterogeneity. We then assessed the potential reason of heterogeneity to see if the effect of intervention might be different in certain populations.

#### 4.3.13 Sensitivity analysis

If numbers permitted, sensitivity analyses was performed on the primary outcomes to consider the impact of the following.

- Allocation concealment (adequate versus inadequate and/or unclear)
- Attrition (<10% vs. ≥10%)
- Imputed inter correlation coefficients (ICC) that have been derived in different ways

#### 4.3.14 Treatment of qualitative research

Qualitative research was outside the scope of this review.

## 5 RESULTS

### 5.1 Description of studies

See: Characteristics of included studies and Characteristics of excluded studies.

#### 5.1.1 Results of the search

**Figure 2:**
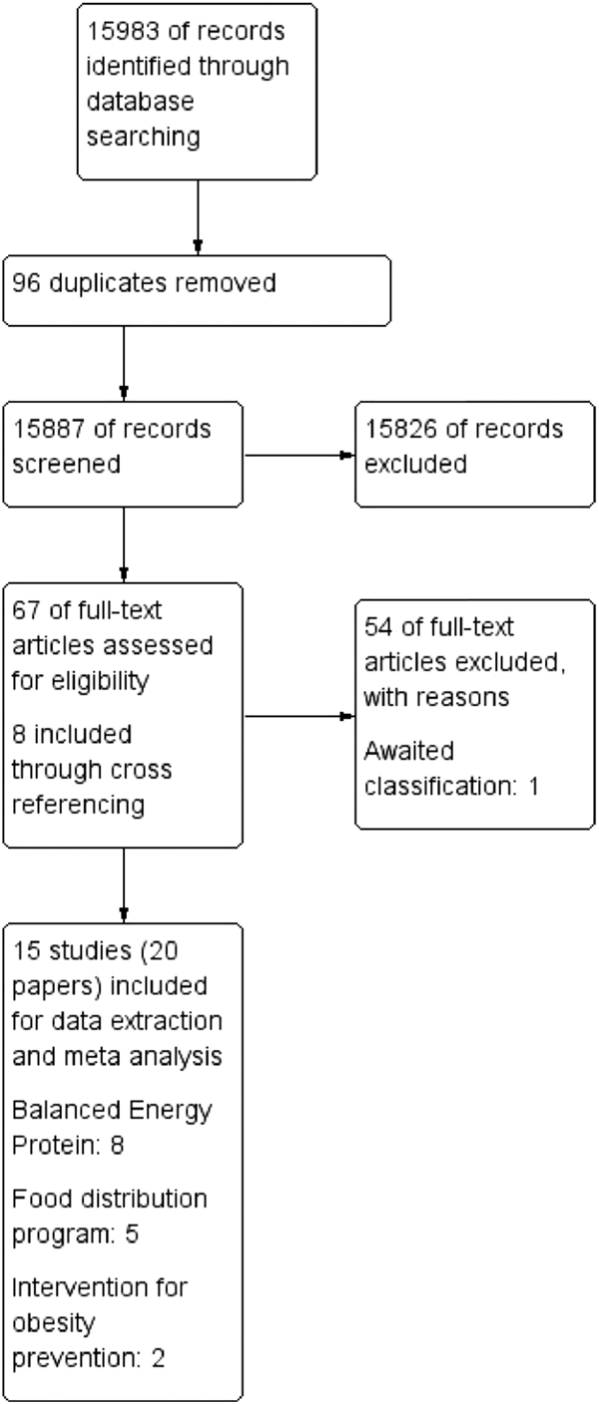
PRISMA flow diagram

The search identified 15,983 records from outlined search engines. After the removal of 96 duplicates, the remaining 15,887 records underwent title and abstract screening using Covidence. On abstract screening, 67 articles were selected for full‐text screening and at the same time eight articles were included through cross referencing. Fifteen studies were finally included for data extraction and meta analysis (see Figure 2).

#### 5.1.2 Included studies

We included 15 studies (see Characteristics of included studies). Of these, eight were on BEP supplementation (Ceesay et al., 1997; Dwarkanath et al., 2016; Girija et al., 1984; Kaseb et al., 2002; Mora et al., 1978a; Prentice et al., 1987; Ross et al., 1985; Tontisirin et al., 1986), five were on food supplementation (Ashorn et al., 2015; Frith et al., 2015; Johnson et al., 2016; Leroy et al., 2016; Mridha et al., 2016), and two were on lifestyle modification for obesity prevention (Aşcı & Rathfisch, 2016; Liu et al., 2017). Of these 15 studies, nine were randomized controlled trials (RCTs) (Aşcı & Rathfisch, 2016, Ashorn et al., 2015; Ceesay et al., 1997; Dwarkanath et al., 2016; Frith et al., 2015; Johnson et al., 2016; Kaseb et al., 2002; Ross et al., 1985; Tontisirin et al., 1986), two were cluster‐randomized controlled trials (cRCTs) (Leroy et al., 2016; Mridha et al., 2016), and four were quasi‐experimental trials (Girija et al., 1984; Liu et al., 2017; Mora et al., 1978a; Prentice et al., 1987).

##### BEP supplementation

Eight studies supplemented BEP (Ceesay et al., 1997; Dwarkanath et al., 2016; Girija et al., 1984; Kaseb et al., 2002; Mora et al., 1978a; Prentice et al., 1987; Ross et al., 1985; Tontisirin et al., 1986) as interventions were <25% energy from protein. These included five randomized controlled trials (Ceesay et al., 1997; Dwarkanath et al., 2016; Kaseb et al., 2002; Ross et al., 1985; Tontisirin et al., 1986), and three were quasi‐experimental studies (Girija et al., 1984; Mora et al., 1978a; Prentice et al., 1987).

###### Outcomes

Primary outcomes reported were miscarriage (Dwarkanath et al., 2016), stillbirth (Ceesay et al., 1997; Dwarkanath et al., 2016; Mora et al., 1978a), perinatal mortality (Ceesay et al., 1997), neonatal mortality (Ceesay et al., 1997), and infant mortality (Ceesay et al., 1997). Secondary outcomes reported were LBW (Ceesay et al., 1997; Dwarkanath et al., 2016; Prentice et al., 1987), preterm birth (Dwarkanath et al., 2016; Mora et al., 1978b), SGA (Ceesay et al., 1997; Dwarkanath et al., 2016; Girija et al., 1984; Mora et al., 1978a; Prentice et al., 1987), birth weight (Ceesay et al., 1997; Dwarkanath et al., 2016; Girija et al., 1984; Kaseb et al., 2002; Mora et al., 1978a; Prentice et al., 1987; Ross et al., 1985; Tontisirin et al., 1986), birth length (Dwarkanath et al., 2016; Tontisirin et al., 1986), and head circumference (Tontisirin et al., 1986).

Meta‐analysis was performed on stillbirth (Ceesay et al., 1997; Dwarkanath et al., 2016; Mora et al., 1978a), LBW (Ceesay et al., 1997; Dwarkanath et al., 2016; Prentice et al., 1987), preterm birth (Dwarkanath et al., 2016; Mora et al., 1978b), SGA (Ceesay et al., 1997; Dwarkanath et al., 2016; Girija et al., 1984; Mora et al., 1978a; Prentice et al., 1987), birth weight (Ceesay et al., 1997; Dwarkanath et al., 2016; Girija et al., 1984; Kaseb et al., 2002; Mora et al., 1978a; Prentice et al., 1987; Ross et al., 1985; Tontisirin et al., 1986), and birth length (Dwarkanath et al., 2016; Tontisirin et al., 1986).

###### Setting

Of included studies, four were conducted in Asia: two each in India (Dwarkanath et al., 2016; Girija et al., 1984), one in Thailand (Tontisirin et al., 1986), and one in Iran (Kaseb et al., 2002), three in Africa: two in Gambia (Ceesay et al., 1997; Prentice et al., 1987); and one in South Africa (Ross et al., 1985), and one in South America (Mora et al., 1978b).

The interventions took place in a varying combinations of villages, clinics, hospitals, healthcare centers, and communities. Four studies were conducted in communities (Ceesay et al., 1997; Mora et al., 1978a; Prentice et al., 1987; Tontisirin et al., 1986), one in slums (Girija et al., 1984), one in healthcare center (Kaseb et al., 2002), one in clinic (Ross et al., 1985), and one in an inpatient hospital setting (Dwarkanath et al., 2016).

Five studies were conducted in rural setting (Ceesay et al., 1997; Girija et al., 1984; Mora et al., 1978a; Prentice et al., 1987; Tontisirin et al., 1986), two in urban (Dwarkanath et al., 2016; Kaseb et al., 2002), and one study failed to provide sufficient detail (Ross et al., 1985).

###### Participants

All participants were healthy pregnant women of child bearing age with no comorbids. The age range of pregnant females was 15–45 years, with the mean age of 25.4 years. Three studies included well nourished pregnant women with a BMI range of 20.5–26.4 (Ceesay et al., 1997; Kaseb et al., 2002; Tontisirin et al., 1986), two studies included undernourished women with mean BMI of 17.4 (Dwarkanath et al., 2016; Mora et al., 1978a), while three studies failed to report BMI of pregnant women (Girija et al., 1984; Prentice et al., 1987; Ross et al., 1985). The maximum population was 12,000 (Ceesay et al., 1997) while two studies failed to report on it (Girija et al., 1984; Mora et al., 1978a).

###### Intervention

The studies on BEP assessed macronutrient supplementation in various forms. For example, Ceesay et al. (1997) and Prentice et al. (1987) provided BEP in the form of biscuits, while other studies provided it in the form of energy.

All studies provided supplementation during pregnancy, however, the timing and duration of supplementation varied from the discovery of pregnancy to the last trimester of pregnancy. Kaseb et al. (2002) implemented comparatively early supplementation starting at the 4th month of pregnancy. One study provided supplementation throughout pregnancy (Dwarkanath et al., 2016). Three studies began supplementation at 20 weeks of gestation (Ceesay et al., 1997; Ross et al., 1985; Tontisirin et al., 1986). One study began supplementation at 24 weeks of gestation (Prentice et al., 1987), and two studies began supplementation in the last trimester of pregnancy (Girija et al., 1984; Mora et al., 1978a).

Four of the BEP studies involved daily supplementation (Ceesay et al., 1997; Dwarkanath et al., 2016). Two studies involved supplementation five times a week (Kaseb et al., 2002; Ross et al., 1985). Prentice et al. (1987) instructed the use of supplements six times a week. Participants in Tontisirin et al. (1986) were instructed to consume the supplement three times a day in addition to their normal home meals. While two studies failed to report on it (Girija et al., 1984; Mora et al., 1978a).

###### Comparison groups

All the included studies under BEP supplementation were provided with routine standard‐of‐care only (Ceesay et al., 1997, Dwarkanath et al., 2016; Girija et al., 1984; Kaseb et al., 2002; Mora et al., 1978a; Prentice et al., 1987; Ross et al., 1985; Tontisirin et al., 1986).

##### Food distribution program

Five studies were included in FDP (Ashorn et al., 2015; Frith et al., 2015; Johnson et al., 2016; Leroy et al., 2016; Mridha et al., 2016), where food supplementation was provided in addition to dietary counseling sessions. These studies included three randomized controlled trials (Ashorn et al., 2015; Frith et al., 2015; Johnson et al., 2016), and two cluster‐randomized controlled trials (Leroy et al., 2016; Mridha et al., 2016).

###### Outcomes

Primary outcomes reported included miscarriage (Mridha et al., 2016), stillbirth (Ashorn et al., 2015; Mridha et al., 2016), perinatal mortality (Ashorn et al., 2015; Mridha et al., 2016), neonatal mortality (Ashorn et al., 2015), and infant mortality (Ashorn et al., 2015). Secondary outcomes reported included maternal mortality (Ashorn et al., 2015; Mridha et al., 2016), anemia (Leroy et al., 2016), LBW (Ashorn et al., 2015; Frith et al., 2015; Johnson et al., 2016; Mridha et al., 2016), preterm birth (Ashorn et al., 2015; Johnson et al., 2016; Mridha et al., 2016), SGA (Ashorn et al., 2015; Johnson et al., 2016; Mridha et al., 2016), birth weight (Ashorn et al., 2015; Frith et al., 2015; Mridha et al., 2016), birth length (Ashorn et al., 2015; Frith et al., 2015; Mridha et al., 2016), head circumference (Frith et al., 2015; Mridha et al., 2016), stunting (Ashorn et al., 2015; Mridha et al., 2016), wasting (Ashorn et al., 2015; Mridha et al., 2016), and underweight (Ashorn et al., 2015; Mridha et al., 2016).

Meta analysis was conducted on perinatal mortality (Ashorn et al., 2015; Mridha et al., 2016), maternal mortality (Ashorn et al., 2015; Mridha et al., 2016), LBW (Ashorn et al., 2015; Frith et al., 2015; Johnson et al., 2016; Mridha et al., 2016), preterm birth (Ashorn et al., 2015; Johnson et al., 2016; Mridha et al., 2016), SGA (Ashorn et al., 2015; Johnson et al., 2016; Mridha et al., 2016), birth weight (Ashorn et al., 2015; Frith et al., 2015; Mridha et al., 2016), birth length (Ashorn et al., 2015; Frith et al., 2015; Mridha et al., 2016), head circumference (Frith et al., 2015; Mridha et al., 2016), stunting (Ashorn et al., 2015; Mridha et al., 2016), wasting (Ashorn et al., 2015; Mridha et al., 2016), and underweight (Ashorn et al., 2015; Mridha et al., 2016).

###### Setting

Three studies were conducted in Africa: one in Malawi (Ashorn et al., 2015), and one each in Gambia (Johnson et al., 2016), and Burundi (Leroy et al., 2016), and two studies were conducted in Asia, in Bangladesh (Frith et al., 2015; Mridha et al., 2016).

The interventions took place in varying combination of slums, communities, hospitals, and villages. Three studies provided intervention in community (Frith et al., 2015; Johnson et al., 2016; Mridha et al., 2016), one in hospital (Ashorn et al., 2015), and one study failed to mention the setting (Leroy et al., 2016).

Three studies were conducted in rural setting (Ashorn et al., 2015; Frith et al., 2015; Johnson et al., 2016), and one study failed to mention sufficient detail on the setting (Leroy et al., 2016).

###### Participants

All participants were healthy pregnant women of child bearing age with no comorbids. The age range of pregnant females was 18–45 years, with the mean age of 25 years. Four studies included well nourished pregnant women with a BMI range of 18.8–23.3 (Ashorn et al., 2015; Frith et al., 2015; Johnson et al., 2016; Mridha et al., 2016), while one study failed to report BMI of pregnant women (Leroy et al., 2016). The maximum population was 279,614 (Mridha et al., 2016) and the minimum population of studies was 15,000 (Johnson et al., 2016). The average population of the studies is 138,653. Study populations were not mentioned by two studies (Frith et al., 2015; Leroy et al., 2016).

###### Intervention groups

Studies on FDP provided nutritional supplement to the pregnant women along with food rations (Leroy et al., 2016), and free medical care such as antenatal/postnatal care (Frith et al., 2015), maternity services or obstetrical care (Mridha et al., 2016), malaria treatment (Ashorn et al., 2015), and HIV counseling (Ashorn et al., 2015).

Two studies had similar interventions, which included a food package providing 118 kcal/day with some micronutrients, and antimalarial intervention (Ashorn et al., 2015; Mridha et al., 2016). Frith et al., (2015) and Johnson et al. (2016) provided food supplementations providing 598 and 746 kcal, respectively. Leroy et al. (2016) failed to specify the nutritional information of the supplement.

Four studies recommended daily use of supplementation (Ashorn et al., 2015; Johnson et al., 2016; Leroy et al., 2016; Mridha et al., 2016), and Frith et al., (2015) provided the intervention six times a week.

The durations of supplementation varied greatly between studies. Two studies enrolled at first trimester of pregnancy and then continued supplementation until six months postpartum (Ashorn et al., 2015; Mridha et al., 2016). Frith et al., (2015) provided supplementation from nine weeks of pregnancy to six months post partum. Johnson et al. (2016) simply provided supplementation from the 20th week of gestation and 3rd month of gestation until birth, respectively. Leroy et al. (2016) provided supplementation for a mean of 1000 days, that is, from pregnancy to 18–24 months post partum.

###### Comparison groups

Participants of all FDP control groups received standard of care (Johnson et al., 2016) with some additional changes between studies. Two studies provided anti‐malarials, HIV pretesting and micro‐nutrition (Ashorn et al., 2015; Mridha et al., 2016), one received bonus pregnancy education and it's respective intervention for shorter duration (Frith et al., 2015), and one received bonus community and general health education (Leroy et al., 2016).

##### Interventions for obesity prevention

Two trials were included in the interventions for obesity prevention (Aşcı & Rathfisch, 2016; Liu et al., 2017). The interventions included dietary and lifestyle counseling aimed at preventing or reducing the risk of obesity.

Of the two included studies (Aşcı & Rathfisch, 2016; Liu et al., 2017), one was a randomized controlled trial (Aşcı & Rathfisch, 2016), and one was a quasi experimental trial (Liu et al., 2017).

###### Outcomes

None of the primary outcomes were reported in the included obesity prevention studies. Secondary outcomes reported were macrosomia (Liu et al., 2017), birth weight (Aşcı & Rathfisch, 2016; Liu et al., 2017), and birth length (Aşcı & Rathfisch, 2016).

Meta‐analysis could only be performed on birth weight (Aşcı & Rathfisch, 2016; Liu et al., 2017).

###### Settings

Of these included studies, one was from Asia: China (Liu et al., 2017), and one study was from Europe: Turkey (Aşcı & Rathfisch, 2016).

The study interventions took place in a tertiary health care center (Liu et al., 2017), and one in a family healthcare center (Aşcı & Rathfisch, 2016; Table 2).

**Table 2:** Matrix (interventions for obesity prevention)

Study (author and year)	Region (Africa/South Asia/South America and Carribean)	Duration of supplementation (whole pregnancy/second trimester/third trimester)	Nutritional status (normal weight/overweight/obese)	Location (rural/urban/mixed)
*Obesity prevention*				
Aşcı and Rathfisch (2016)	Europe	Whole pregnancy	Normal weight	Urban
Liu et al. (2017)	East Asia	Second trimester	Normal weight	Urban

None of the studies reported sufficient information to judge the level of infrastructure (i.e., urban or rural) of the study setting.

###### Participants

All participants were healthy pregnant women with no additional comorbid conditions. The mean age of pregnant females was 25.5 years. Both studies included healthy normal weight women.

Study population was only reported by one study (Aşcı & Rathfisch, 2016) with 21,000 people, whereas the other study failed to report on study population (Liu et al., 2017).

###### Intervention groups

Intervention for obesity prevention included dietary interventions along with nutrition education, counseling sessions on healthy lifestyle and behavioral change (Aşcı & Rathfisch, 2016; Liu et al., 2017).

###### Comparison groups

Aşcı and Rathfisch (2016) and Liu et al. (2017) provided participants with the standard‐of‐care.

#### 5.1.3 Excluded studies

In totality, we excluded 54 studies from the review. Seven trials were excluded because the population did not include healthy pregnant women (Anonymous, 1995; Edrisi et al., 2018; Ello‐Martin et al., 2007; Perichart‐Perera et al., 2009; Pollak et al., 2014; Wang et al., 2015; Zhang, 1997), 23 trials were excluded because they were not conducted in LMICs (Blackwell et al., 1973; Clements et al., 2016; Dodd et al., 2014, 2015; Guelinckx et al., 2010; Halkjaer et al., 2016; Hawkins et al., 2015; Huseinovic et al., 2017; Korpi‐Hyövälti et al., 2012; McGowan et al., 2013; Mueller & Pollitt, 1984; Mustila et al., 2018; Paul & Olson, 2013; Peccei et al., 2017; Phelan et al., 2011; Piirainen et al., 2006; Polley et al., 2002; Rauh et al., 2013; Renault et al., 2015; Rhodes et al., 2010; Ruchat et al., 2012; Rush et al., 1980), 13 of the trials were excluded as they did not report on any of our outcomes of interest (Alderman et al., 2014; Changamire et al., 2014; Fraser et al., 1983; Harding et al., 2017; Hawkesworth et al., 2008, 2011; Hoa et al., 2005; Huybregts et al., 2013; Jahan et al., 2014; Kardjati et al., 1990; Matias et al., 2016; Sahariah et al., 2016; Thornton et al., 2009b), three were excluded because of no standard of care control group (Kusin et al., 1992; van Steenbergen et al., 1989; Winkvist et al., 1998), five studies were excluded because the population was given the wrong intervention/control (micronutrient supplementation) (Devi et al., 2017; Huybregts et al., 2009; Katz et al., 2006; Nossier et al., 2015; Saville et al., 2018), and three studies were excluded because of wrong study design; two were letters to the editor (Asbee et al., 2009; Thornton et al., 2009a) and one was a legislative document (Anonymous, 2016). See Characteristics of excluded studies for more details.

##### BEP supplementation

Out of the 54 excluded studies, 13 were on BEP supplementation (Anonymous, 1995; Blackwell et al., 1973; Devi et al., 2017; Hawkesworth et al., 2011; Kardjati et al., 1990; Kusin et al., 1992; Mueller & Pollitt, 1984; Rush et al., 1980; Saville et al., 2018; van Steenbergen et al., 1989; Wang et al., 2015; Winkvist et al., 1998; Zhang, 1997). Three studies (Anonymous, 1995; Wang et al., 2015; Zhang, 1997) were excluded due to their wrong patient population of interest; three were excluded due to missing standard of care control group (Kusin et al., 1992; van Steenbergen et al., 1989; Winkvist et al., 1998), and two studies were excluded due to wrong intervention as they provided BEP along with micronutrient supplementation (Devi et al., 2017; Saville et al., 2018). Zhang (1997) only included IUGR births, Wang et al. (2015) only included women suffering from GDM, and Anonymous (1995) did not exclusively include pregnant women. Three studies were excluded as they were conducted in higher income countries (Blackwell et al., 1973; Mueller & Pollitt, 1984; Rush et al., 1980), and two studies failed to report any of the outcomes of our interest (Hawkesworth et al., 2011; Kardjati et al., 1990).

##### Food distribution program

Out of the excluded studies, 16 were on FDP (Alderman et al., 2014; Changamire et al., 2014; Edrisi et al., 2018; Ello‐Martin et al., b; Fraser et al., 1983; Harding et al., 2017; Hawkesworth et al., 2008; Hoa et al., 2005; Huseinovic et al., 2017; Huybregts et al., 2013; Jahan et al., 2014; Katz et al., 2006; Matias et al., 2016; Perichart‐Perera et al., 2009; Piirainen et al., 2006; Sahariah et al., 2016). Ten of the studies did not report any outcomes of our interest (Alderman et al., 2014; Changamire et al., 2014; Fraser et al., 1983; Harding et al., 2017; Hawkesworth et al., 2008; Hoa et al., 2005; Huybregts et al., 2013; Jahan et al., 2014; Matias et al., 2016; Sahariah et al., 2016). Three (Edrisi et al., 2018; Ello‐Martin et al., 2007; Perichart‐Perera et al., 2009) were excluded due to their wrong patient population; Edrisi et al. (2018) included men in its sample, Ello‐Martin et al. (2007) included only obese participants and Perichart‐Perera et al. (2009) only included GDM women. Two studies were excluded as they were conducted in high‐income countries (Huseinovic et al., 2017; Piirainen et al., 2006). One study was excluded due to delivering the wrong intervention; Katz et al. (2006) only provided micronutrient supplementation to its intervention group.

##### Interventions for obesity prevention

Out of the excluded studies, 23 were on obesity prevention interventions (Anonymous, 2016; Asbee et al., 2009; Clements et al., 2016; Dodd et al., 2014, 2015; Guelinckx et al., 2010; Halkjaer et al., 2016; Hawkins et al., 2015; Korpi‐Hyövälti et al., 2012; McGowan et al., 2013; Mustila et al., 2018; Paul & Olson, 2013; Peccei et al., 2017; Phelan et al., 2011; Pollak et al., 2014; Polley et al., 2002; Rauh et al., 2013; Renault et al., 2015; Rhodes et al., 2010; Ruchat et al., 2012; Thornton et al., 2009a; Thornton et al., 2009b). Eighteen studies failed to report any outcomes of our interest (Clements et al., 2016; Dodd et al., 2014, 2015; Guelinckx et al., 2010; Halkjaer et al., 2016; Hawkins et al., 2015; Korpi‐Hyövälti et al., 2012; McGowan et al., 2013; Mustila et al., 2018; Paul & Olson, 2013; Peccei et al., 2017; Phelan et al., 2011; Polley et al., 2002; Rauh et al., 2013; Renault et al., 2015; Rhodes et al., 2010; Ruchat et al., 2012). Three of the studies were excluded as they were not scientific papers; Anonymous (2016) was a legislative guideline, and Thornton et al. (2009a) and Asbee et al. (2009) were both “Letter to the Editors.” One study, Thornton et al. (2009b) did not report on outcomes of interest. One study, Pollak et al. (2014) was excluded as its patient population of interest was previously diabetic.

### 5.2 ROB in included studies

**Figure 3:**
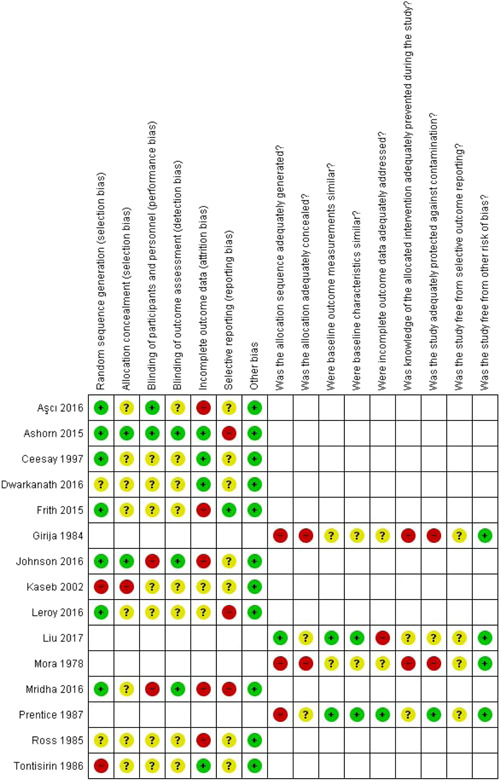
Risk of bias summary: review authors' judgments about each risk of bias item for each included study

#### 5.2.1 BEP supplementation

##### Randomized control trials

Five RCTs (Ceesay et al., 1997; Kaseb et al., 2002; Dwarkanath et al., 2016; Ross et al., 1985; Tontisirin et al., 1986) were related to BEP supplementation during pregnancy.

#### 5.2.2 Food distribution program

##### Randomized control trials

Three RCTs (Ashorn et al., 2015; Frith et al., 2015; Johnson et al., 2016) and two cluster‐RCTs (Leroy et al., 2016; Mridha et al., 2016) were related to FDP.

#### 5.2.3 Interventions for obesity prevention

##### Randomized control trials

One RCT was related to intervention for obesity prevention (Aşcı & Rathfisch, 2016).

#### 5.2.4 Allocation (selection bias)

##### BEP supplementation

###### Sequence generation

Adequate randomization was done in one trial (Ceesay et al., 1997), due to which the ROB was low. Stratified design according to the village size was used for random sequence generation (Ceesay et al., 1997).

Two trials had high risk for sequence generation (Kaseb et al., 2002; Tontisirin et al., 1986). In Kaseb et al., (2002) and Tontisirin et al. (1986), the experimental and control groups were selected sequentially which led to an increased predictability among personnel and participants making the ROB high. The randomization method was not clearly mentioned in two trials (Dwarkanath et al., 2016; Ross et al., 1985), making the ROB unclear.

###### Allocation concealment

The ROB for allocation concealment was high in one trial (Kaseb et al., 2002), because participants were selected sequentially by the women referred to the health care of Ghaemieh and Baghfayz. The method of allocation concealment was not clearly mentioned in four trials (Ceesay et al., 1997; Dwarkanath et al., 2016; Ross et al., 1985; Tontisirin et al., 1986), making the ROB unclear.

##### Food distribution program

###### Sequence generation

Five trials (Ashorn et al., 2015; Frith et al., 2015; Johnson et al., 2016; Leroy et al., 2016; Mridha et al., 2016) adequately randomized participants; which were achieved by block randomization (Ashorn et al., 2015; Johnson et al., 2016), stratified design (Mridha et al., 2016), factorial design (Frith et al., 2015), and through a lottery event (Leroy et al., 2016).

###### Allocation concealment

ROB was low for allocation concealment in two studies. It was low because of use of opaque, sealed envelopes (Ashorn et al., 2015), and numbered boxes (Johnson et al., 2016). The ROB for allocation concealment was unclear in three trials (Frith et al., 2015; Leroy et al., 2016, Mridha et al., 2016).

##### Interventions for obesity prevention

###### Sequence generation

One trial adequately randomized participants (Aşcı & Rathfisch, 2016), using drawing randomization lots.

###### Allocation concealment

One trial failed to mention any method of allocation concealment (Aşcı & Rathfisch, 2016).

#### 5.2.5 Blinding (performance bias and detection bias)

##### BEP supplementation

###### Blinding of participants and personnel

Five RCTs did not mention blinding of participants/personnel distinctly, and had unclear ROB (Ceesay et al., 1997; Dwarkanath et al., 2016; Kaseb et al., 2002; Ross et al., 1985; Tontisirin et al., 1986).

###### Blinding for outcome assessors

Five RCTs failed to report on blinding of outcome assessment making them unclear ROB (Ceesay et al., 1997; Dwarkanath et al., 2016; Kaseb et al., 2002; Ross et al., 1985; Tontisirin et al., 1986).

##### Food distribution program

###### Blinding of participants and personnel

One RCT (Ashorn et al., 2015) was at low risk due to adequate blinding or because blinding was not required due to the type of intervention occurring. Two RCTs which did not mention blinding of participants/personnel distinctly were at unclear ROB (Frith et al., 2015; Leroy et al., 2016). There was a high ROB concerning blinding in two trial as the interviewers were not blinded to the interventions that the women in different arms of the trial would be receiving (Johnson et al., 2016; Mridha et al., 2016).

###### Blinding of outcome assessors

Three of the trials were at low ROB because they had performed adequate blinding of outcome assessors (Ashorn et al., 2015; Johnson et al., 2016; Mridha et al., 2016). Two trials did not mention blinding of outcome assessor and hence, had an unclear ROB (Frith et al., 2015; Leroy et al., 2016).

##### Interventions for obesity prevention

###### Blinding of participants and personnel

One trial reported to perform adequate blinding of participants and personnel (Aşcı & Rathfisch, 2016).

###### Blinding of outcome assessors

One trial had insufficient information to permit judgment (Aşcı & Rathfisch, 2016).

#### 5.2.6 Incomplete outcome data (attrition bias)

##### BEP supplementation

###### Incomplete outcome data

Studies that were at low risk addressed incomplete outcome data adequately in three studies (Ceesay et al., 1997; Dwarkanath et al., 2016; Tontisirin et al., 1986). The issue of incomplete outcome data was not addressed adequately in one trial (Kaseb et al., 2002) putting it at an unclear ROB. Two studies were at high risk of attrition bias due to significant loss to follow‐up from both intervention and control groups (Ross et al., 1985).

##### Food distribution program

###### Incomplete outcome data

This issue was addressed reasonably well due to low attrition rates by one study (Ashorn et al., 2015). The issue of incomplete outcome data was not addressed adequately in one trial (Leroy et al., 2016) putting it at an unclear ROB. Three studies were at high risk of attrition bias due to significant loss to follow‐up from both intervention and control groups (Frith et al., 2015; Johnson et al., 2016; Mridha et al., 2016).

##### Interventions for obesity prevention

###### Incomplete outcome data

One study was at high risk of attrition bias due to significant loss to follow‐up from both intervention and control groups (Aşcı & Rathfisch, 2016).

#### 5.2.7 Selective reporting (reporting bias)

##### BEP supplementation

###### Selective reporting

Five of the trials had unclear ROB as there was insufficient evidence to disregard the notion of selective reporting (Ceesay et al., 1997; Dwarkanath et al., 2016; Kaseb et al., 2002; Ross et al., 1985; Tontisirin et al., 1986).

##### Food distribution program

###### Selective reporting

One trial had low ROB when there was sufficient evidence to disregard the notion of selective reporting (Frith et al., 2015), and one had unclear ROB when there was not (Johnson et al., 2016). Two trials were at high risk because they were unable to report all the outcomes mentioned in the protocol (Ashorn et al., 2015; Leroy et al., 2016).

##### Interventions for obesity prevention

One trial had unclear ROB as there was insufficient evidence to disregard the notion of selective reporting (Aşcı & Rathfisch, 2016).

#### 5.2.8 Other potential sources of bias

##### BEP supplementation

All five of the BEP RCTs were free from other sources of bias (Ceesay et al., 1997; Kaseb et al., 2002; Dwarkanath et al., 2016; Ross et al., 1985; Tontisirin et al., 1986).

##### Food distribution program

All five of the FDP RCTs were free from other sources of bias (Ashorn et al., 2015; Frith et al., 2015; Johnson et al., 2016; Leroy et al., 2016; Mridha et al., 2016).

##### Interventions for obesity prevention

One study on obesity prevention was free from other sources of bias (Aşcı & Rathfisch, 2016).

#### 5.2.9 Quasi‐experimental

##### BEP supplementation

There were three quasi experimental trials related to BEP supplementation in pregnant women (Girija et al., 1984; Mora et al., 1978b; Prentice et al., 1987).

##### Food distribution program

There were no quasi experimental trials related to FDP.

##### Interventions for obesity prevention

There was only one quasi experimental trial on interventions related to obesity prevention in pregnant women (Liu et al., 2017).

#### 5.2.10 Was the allocation sequence adequately generated?

##### BEP supplementation

Adequate randomization was not generated in three trials (Girija et al., 1984; Mora et al., 1978b; Prentice et al., 1987), making their ROB high.

##### Interventions for obesity prevention

Adequate randomization was done in one trial (Liu et al., 2017), due to which the ROB was low.

#### 5.2.11 Was the allocation adequately concealed?

##### BEP supplementation

Two quasi experimental studies did not conceal allocation adequately, making the ROB high (Girija et al., 1984; Mora et al., 1978b). The ROB for allocation concealment was unclear in one trial (Prentice et al., 1987) as it was not mentioned in the respective text.

##### Interventions for obesity prevention

The ROB for allocation concealment was unclear in the only included obesity prevention trial (Liu et al., 2017) as it was not mentioned in the respective text.

#### 5.2.12 Were baseline outcome measurements similar?

##### BEP supplementation

Baseline outcomes were similar across groups in one trial in BEP supplementation program (Prentice et al., 1987). Two trials failed to report on it making the ROB unclear (Girija et al., 1984; Mora et al., 1978b).

##### Interventions for obesity prevention

Baseline outcomes were similar across groups in the only trial in obesity prevention program (Liu et al., 2017).

#### 5.2.13 Were baseline characteristics similar?

##### BEP supplementation

Baseline characteristics were similar across groups in one trial (Prentice et al., 1987) in BEP supplementation program. Two trials failed to report on it making the ROB unclear (Girija et al., 1984; Mora et al., 1978b).

##### Interventions for obesity prevention

Baseline characteristics were similar across groups in one trial (Liu et al., 2017) in obesity prevention program.

#### 5.2.14 Were incomplete outcome data adequately addressed?

##### BEP supplementation

One trial was at low risk addressing incomplete outcome data adequately (Prentice et al., 1987) on BEP supplementation, while two trials failed to report on it making the ROB unclear (Girija et al., 1984; Mora et al., 1978b).

Liu et al., 2017 had a substantial attrition rate with 10.9% attrition as 11 of the 101 participants were lost to follow‐up.

#### 5.2.15 Was knowledge of the allocated intervention adequately prevented during the study?

##### BEP supplementation

In one trial, there was an unclear ROB with regards to prevention of knowledge of the allocated interventions (Prentice et al., 1987), while two studies failed to prevent knowledge of the allocated interventions, making the ROB high (Girija et al., 1984; Mora et al., 1978b).

##### Interventions for obesity prevention

There was an unclear ROB with regards to prevention of knowledge of the allocated interventions in one trial (Liu et al., 2017).

#### 5.2.16 Was the study adequately protected against contamination?

##### Balanced energy protein

Adequate measures were taken in one trial (Prentice et al., 1987) with regards to contamination, while two trials failed to take measures, making the ROB high (Girija et al., 1984; Mora et al., 1978b).

##### Interventions for obesity prevention

With regards to contamination, there was insufficient data to make a judgment in one case (Liu et al., 2017).

#### 5.2.17 Was the study free from selective outcome reporting?

##### BEP supplementation

Three trials on BEP supplementation had unclear ROB since there was insufficient evidence to disregard the notion of selective reporting (Girija et al., 1984; Prentice et al., 1987; Mora et al., 1978b).

##### Interventions for obesity prevention

One trial was at unclear risk of selective outcome reporting (Liu et al., 2017) in obesity prevention studies.

#### 5.2.18 Was the study free from other risks of bias?

##### Balanced energy protein

Three trials (Girija et al., 1984; Prentice et al., 1987; Mora et al., 1978b) on BEP supplementation were free from other sources of bias.

##### Interventions for obesity prevention

The one trial (Liu et al., 2017) on obesity prevention was free from other sources of bias.

### 5.3 Effects of interventions

#### 5.3.1 Comparison 1: BEP supplementation versus control

Eight trials (Ceesay et al., 1997; Dwarkanath et al., 2016; Girija et al., 1984; Kaseb et al., 2002; Mora et al., 1978b; Prentice et al., 1987; Ross et al., 1985; Tontisirin et al., 1986), involving 12,744 participants assessed the impact of BEP supplementation on pregnant women.

#### 5.3.2 Primary outcomes

Among primary outcomes, included studies reported on miscarriage (Dwarkanath et al., 2016), stillbirth (Ceesay et al., 1997; Dwarkanath et al., 2016; Mora et al., 1978b), perinatal mortality (Ceesay et al., 1997), neonatal mortality (Ceesay et al., 1997), and infant mortality (Ceesay et al., 1997). Studies on BEP failed to report on maternal BMI, and under five mortality.

##### Maternal outcomes

Studies on BEP supplementation failed to report on maternal outcomes (i.e., maternal BMI).

##### Fetal and newborn outcomes

###### Miscarriage

One study (Dwarkanath et al., 2016) reported on the incidence of miscarriage, as a spontaneous abortion at 24.2 weeks. BEP supplementation may make little or no difference in number of miscarriages (RR, 1.00; 95% CI, 0.07–14.21, one study, 24 participants; low quality on GRADE) (Analysis 1.1; Tables 3 and 4).

**Table 3:** Dichotomous lone reported outcomes

Outcome	Study		Intervention		Control		Risk ratio (95% CI)
			Events	Total	Events	Total	
*Balanced energy protein supplementation*							
Miscarriage	Dwarkanath et al. (2016)		1	12	1	12	1.00 (0.07, 14.21)
Perinatal mortality	Ceesay et al. (1997)		22	737	42	709	0.50 (0.30, 0.84)
Neonatal mortality	Ceesay et al. (1997)		17	737	28	709	0.58 (0.32, 1.06)
Infant mortality	Ceesay et al. (1997)		28	737	27	709	1.00 (0.59, 1.68)
*Food distribution program*							
Miscarriage	Mridha et al. (2016)		56	1047	178	2964	0.89 (0.67, 1.19)
Neonatal mortality	Ashorn et al. (2015)		8	414	18	427	0.46 (0.20, 1.04)
Infant mortality	Ashorn et al. (2015)		0	414	1	427	0.34 (0.01, 8.41)
Anemia of pregnant women	Leroy et al. (2016)	T24	154	414	223	419	0.70 (0.60, 0.82)
		T18	359	849	224	419	0.79 (0.70, 0.89)
*Obesity prevention*							
Macrosomia	Liu et al. (2017)		4	45	7	45	0.57 (0.18, 1.82)

*T24: program benefits provided during pregnancy and till 23.9 months postpartum.

**T18: program benefits provided during pregnancy and till 18 months postpartum.

**Table 4:** Balanced energy protein compared to control for maternal, neonatal and childhood outcomes

Balaced energy protein compared to control for maternal, neonatal and childhood outcomes						
Patient or population: Pregnant women						
Setting: Developing countries						
Intervention: Balanced energy protein						
Comparison: Control						
Outcomes	Anticipated absolute effects (95% CI)^b^		Relative effect (95% CI)	No. of participants (studies)	Certainty of the evidence (GRADE)	Comments
	Risk with control (newly added)	Risk with balanced energy protein				
Miscarriage	Study population		RR 1.00	24	⊕⊕⊝⊝	
			(0.07–14.21)	(1 RCT)	Low^a^ ^c^
	83 per 1000	83 per 1000			
		(6–1000)			
Stillbirth	Study population		RR 0.39	1913	⊕⊕⊝⊝	
			(0.19–0.80)	(3 RCTs)	Low^c^ ^d^	
	28 per 1000	11 per 1000				
		(5–22)				
Perinatal Mortality	Study population		RR 0.50	1446	⊕⊝⊝⊝	
			(0.30–0.84)	(1 RCT)	Very low^c^ ^d^ ^e^
	59 per 1000	30 per 1000			
		(18–50)			
Neonatal Mortality	Study population		RR 0.58	1446	⊕⊝⊝⊝	
			(0.32–1.06)	(1 RCT)	Very low^c^ ^d^ ^f^	
	39 per 1000	23 per 1000				
		(13–42)				
Infant Mortality	Study population		RR 1.00	1446	⊕⊕⊕⊝	
			(0.59–1.68)	(1 RCT)	Moderate^c^
	38 per 1000	38 per 1,000			
		(22–64)			

*Note*: GRADE Working Group grades of evidence: *High certainty*: We are very confident that the true effect lies close to that of the estimate of the effect. *Moderate certainty*: We are moderately confident in the effect estimate: The true effect is likely to be close to the estimate of the effect, but there is a possibility that it is substantially different. *Low certainty*: Our confidence in the effect estimate is limited: The true effect may be substantially different from the estimate of the effect. *Very low certainty*: We have very little confidence in the effect estimate: The true effect is likely to be substantially different from the estimate of effect.

Abbreviations: CI, confidence interval; OR, odds ratio; RR, risk ratio.

^a^ Sequence generation, allocation concealment, blinding of personnel, participants, outcome assessor and selective reporting is unclear.

^b^ *The risk in the intervention group* (and its 95% confidence interval) is based on the assumed risk in the comparison group and the *relative effect* of the intervention (and its 95% CI).

^c^ Since mortality is a not frequently occurring outcome, so we mark it as serious.

^d^ Allocation concealment, blinding of personnel, participants and outcome assessor is unclear.

^e^ *p* value is .008, which shows existence of heterogeneity.

^f^ *p* value is .08, which shows existence of heterogeneity.

###### Stillbirth

**Figure 4:**
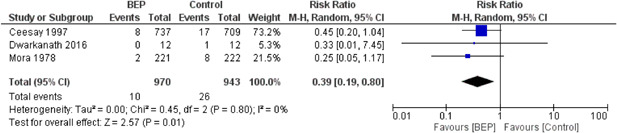
(Analysis 1.2) Forest plot of comparison: 1 balanced energy protein versus control, outcome: 1.2 stillbirth

Three studies (Ceesay et al., 1997; Dwarkanath et al., 2016; Mora et al., 1978b) reported on stillbirths. BEP supplementation may reduce incidence of stillbirths by 61% (RR, 0.39; 95% CI, 0.19–0.80, three studies, 1913 participants; heterogeneity: *χ* ^2^ *p* = .80, *I* ^2^ = 0%, low quality on GRADE) (Analysis 1.2; Figure 4; Table 4).

Upon subgroup analysis based on nutritional status, an insignificant reduction was reported in the rate of stillbirths in studies conducted on well nourished women (RR, 0.45; 95% CI, 0.20–1.04, one study, 1446 participants) (Ceesay et al., 1997), and on under‐nourished women (RR, 0.27; 95% CI, 0.07–1.05; two studies, 467 participants) (Dwarkanath et al., 2016; Mora et al., 1978b). There was no difference between the well‐nourished and under‐nourished women in stillbirths, however the overall effect was significant (RR, 0.39; 95% CI, 0.19–0.80, three studies, 1913 participants; heterogeneity: *χ* ^2^ *p* = .80, *I* ^2^ = 0%) Analysis 2.1. The subgroup analyses on the basis of geographical setting, location, and duration of supplementation in different trimesters of pregnancy show similar results as the subgroup analysis on the basis of nutritional status (Analysis 3.1; Analysis 4.1; Analysis 5.1; Table 5).

**Table 5:** Matrix (balanced energy protein supplementation)

Study (author and year)	Region (Africa/South Asia/South America and Carribean)	Duration of supplementation (whole pregnancy/second trimester/third trimester)	Nutritional status (pregnant women defined based on BMI) undernourished (BMI < 18.5) vs. well nourished (BMI > 18.5)	Location (rural/urban/mixed)
*Balanced energy protein (BEP)*				
Ceesay et al. (1997)	West Africa	Second trimester	Well nourished	Rural
Dwarkanath et al. (2016)	South East Asia	Whole pregnancy	Undernourished	Urban
Girija et al. (1984)	South Asia	Third trimester	Not specified	Rural
Kaseb et al. (2002)	Middle East	Second trimester	Well nourished	Urban
Mora et al. (1978b)	South America	Third trimester	Undernourished	Rural
Prentice et al. (1987)	West Africa	Whole pregnancy	Not specified	Rural
Ross et al. (1985)	South Africa	Second trimester	Not specified	Not specified
Tontisirin et al. (1986)	Southeast Asia	Third trimester	Well nourished	Rural

###### Perinatal mortality

One study (Ceesay et al., 1997) reported on perinatal mortality which showed that BEP supplementation may reduce incidence of perinatal mortality by 50% (RR, 0.50; 95% CI, 0.30–0.84; one study,1446 participants; low quality on GRADE) (Analysis 1.3; Tables 3 and 4).

###### Neonatal mortality

One study (Ceesay et al., 1997) reported on neonatal mortality which showed that BEP supplementation may not have any effect on neonatal mortality (RR, 0.58; 95% CI, 0.32–1.06; one study, 1446 participants; moderate quality of evidence on GRADE) (Analysis 1.4; Tables 3 and 4).

##### Child outcomes

###### Infant mortality

One study (Ceesay et al., 1997) reported on infant mortality which showed that BEP supplementation does not probably have any effect on infant mortality (RR, 1.00; 95% CI, 0.59–1.68; one study, 1446 participants; moderate quality of evidence on GRADE) (Analysis 1.5; Tables 3 and 4).

#### 5.3.3 Secondary outcomes

##### Maternal outcomes

The BEP trials failed to report on maternal secondary outcomes including maternal mortality, pre‐eclampsia, placental abruption, overweight, obesity, anemia, and iron deficiency anemia.

##### Fetal outcomes

The BEP trials failed to report on fetal secondary outcomes, that is, congenital anomalies.

##### Newborn outcomes

The included studies on BEP intervention failed to report on macrosomia as an outcome.

###### Low birth weight

Three studies (Ceesay et al., 1997; Dwarkanath et al., 2016; Prentice et al., 1987) reported on LBW. BEP supplementation showed a significant 40% reduction in the incidence of LBW births (birth weight <2500 g) (RR, 0.60; 95% CI, 0.41–0.86, three studies,1,830 participants; heterogeneity: *χ* ^2^ *p* = .25, *I* ^2^ = 27%) (Analysis 1.6).

###### Preterm birth

Two studies (Dwarkanath et al., 2016; Mora et al., 1978b) reported on preterm birth. BEP supplementation showed no impact on preterm births (RR, 0.86; 95% CI, 0.50–1.46, two studies, 467 participants, heterogeneity: *χ* ^2^ *p* = .54, *I* ^2^ = 0%) (Analysis 1.7).

###### Small‐for‐gestational age

Five studies (Ceesay et al., 1997; Dwarkanath et al., 2016; Girija et al., 1984; Mora et al., 1978b; Prentice et al., 1987) reported on SGA babies. BEP supplementation showed a significant 29% reduction in risk of SGA babies (RR, 0.71; 95% CI, 0.54–0.94; five studies, 1844 participants; heterogeneity: *χ* ^2^ *p* = .28, *I* ^2^ = 21%) (Analysis 1.8).

###### Birth weight

Eight studies (Ceesay et al., 1997; Dwarkanath et al., 2016; Girija et al., 1984; Kaseb et al., 2002; Mora et al., 1978b; Prentice et al., 1987; Ross et al., 1985; Tontisirin et al., 1986) reported on birth weight. BEP supplementation showed a significant 107.28 g increase in birth weight (MD, 107.28 g; 95% CI, 68.51–146.04, eight studies, 2190 participants; heterogeneity: *χ* ^2^ *p* = .13, *I* ^2^ = 35%) (Analysis 1.9).

###### Birth length

Two studies (Dwarkanath et al., 2016; Tontisirin et al., 1986) reported on birth length. BEP supplementation showed no impact on birth length (MD, 0.28 cm; 95% CI, −0.36–0.92, two studies, 67 participants; heterogeneity: *χ* ^2^ *p* = .77, *I* ^2^ = 0%) (Analysis 1.10).

###### Head circumference

One study (Tontisirin et al., 1986) reported on head circumference. BEP had no impact on head circumference (MD, 0.54 cm; 95% CI, −0.20–1.29, one study, 71 participants) (Analysis 1.11; Table 6).

**Table 6:** Continuous lone reported data

Outcome	Study		Intervention			Control			Mean difference (95% CI)
			Mean	*SD*	Total	Mean	*SD*	Total	
*Balanced energy protein supplementation*									
Head circumference (cm)	Tontisirin et al. (1986)	Group 1	35.2	1.2	28	34.4	1.3	7	0.80 (−0.26, 1.86)
		Group 2	34.7	1.4	28	34.4	1.3	8	0.30 (−0.74, 1.34)
*Obesity prevention*									
Birth weight (g)	Liu et al. (2017)		3339	301	45	3536	435	45	−197.00 (−351.56, −42.44)
Birth length (cm)	Aşcı and Rathfisch (2016)		50.04	1.78	45	50.4	1.9	45	−0.36 (−1.12, 0.40)

*T24: program benefits provided during pregnancy and till 23.9 months postpartum.

**T18: program benefits provided during pregnancy and till 18 months postpartum.

##### Child outcomes

The included studies on BEP failed to report on stunting, wasting, underweight, developmental outcomes, respiratory disease, allergic disease, hemoglobin concentration, and iron deficiency anemia in children.

#### 5.3.4 Comparison 2: Food program versus control

We included five trials (Ashorn et al., 2015; Frith et al., 2015; Johnson et al., 2016; Leroy et al., 2016; Mridha et al., 2016), involving a total of 46,442 participants to study the effect of FDP in pregnancy on neonatal and maternal outcomes.

#### 5.3.5 Primary outcomes

Among primary outcomes, included studies reported on miscarriage (Mridha et al., 2016), stillbirth (Ashorn et al., 2015; Mridha et al., 2016), perinatal mortality (Ashorn et al., 2015; Mridha et al., 2016), neonatal mortality (Ashorn et al., 2015), and infant mortality (Ashorn et al., 2015). Included studies on FDP failed to report on any other primary outcomes including maternal BMI and under‐five mortality.

##### Maternal outcomes

None of the included trials on FDP reported on mean maternal BMI.

##### Fetal and newborn outcomes

###### Miscarriage

One study (Mridha et al., 2016) reported on rates of miscarriage. FDP may show little or no difference in number of miscarriages (RR, 0.89; 95% CI, 0.67–1.19; random effect, one study, 4,011 participants; low quality on GRADE) (Analysis 6.1; Tables 3 and 7).

**Table 7:** Food distribution program compared to control for maternal, neonatal and childhood outcomes

Food distribution program compared to control for maternal, neonatal and childhood outcomes						
Patient or population: Pregnant women						
Setting: Developing countries						
Intervention: Food distribution program						
Comparison: Control						
Outcomes	Anticipated absolute effects (95% CI)^a^		Relative effect (95% CI)	No. of participant (studies)	Certainty of the evidence (GRADE)	Comments
	Risk with Control	Risk with food distribution program				
Miscarriage	Study population		RR 0.89 (0.67–1.19)	4011 (1 RCT)	⊕⊕⊝⊝ Low^b^ ^c^	
	60 per 1000	53 per 1000 (40–71)			
Perinatal mortality	Study population		RR 0.67 (0.41–1.09)	4852 (2 RCTs)	⊕⊕⊝⊝ Low^c^ ^d^	
	24 per 1000	16 per 1000 (10–26)				
Neonatal mortality	Study population		RR 0.46 (0.20–1.04)	841 (1 RCT)	⊕⊕⊝⊝ Low^e^ ^f^	
	42 per 1000	19 per 1000 (8–44)			
Infant mortality	Study population		RR 0.34 (0.01–8.41)	841 (1 RCT)	⊕⊕⊕⊝ Moderate^f^	
	2 per 1000	1 per 1000 (0–20)				

*Note*: GRADE Working Group grades of evidence: *High certainty*: We are very confident that the true effect lies close to that of the estimate of the effect. *Moderate certainty*: We are moderately confident in the effect estimate: The true effect is likely to be close to the estimate of the effect, but there is a possibility that it is substantially different. *Low certainty*: Our confidence in the effect estimate is limited: The true effect may be substantially different from the estimate of the effect. *Very low certainty*: We have very little confidence in the effect estimate: The true effect is likely to be substantially different from the estimate of effect.

Abbreviations: CI, confidence interval; OR, odds ratio; RR, risk ratio.

^a^ *The risk in the intervention group* (and its 95% confidence interval) is based on the assumed risk in the comparison group and the *relative effect* of the intervention (and its 95% CI).

^b^ The study is at high risk of blinding of participants and personnel, incomplete outcome data and selective reporting. It was unclear for allocation concealment.

^c^ Number of events are <300.

^d^ Out of two studies, one study is at high risk for blinding of participants, incomplete outcome assessment and selective reporting.

^e^ *p* value is .06, which shows existence of heterogeneity.

^f^ Since mortality is not a frequently occurring outcome, thus we mark it as serious.

###### Perinatal mortality

**Figure 5:**
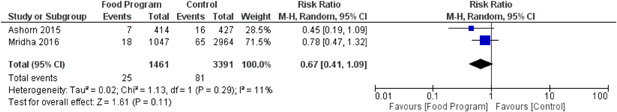
(Analysis 6.2) Forest plot of comparison: 6 Food distribution program vs control, outcome: 6.2 Perinatal mortality

Two studies (Ashorn et al., 2015; Mridha et al., 2016) reported on perinatal mortality, which showed that FDP may reduce the incidence of perinatal mortality by 33% (RR, 0.67; 95% CI, 0.41–1.09, two studies, 4852 participants; heterogeneity: *χ* ^2^ = 0.29, *I* ^2^ = 11%, low quality on GRADE) (Analysis 6.2; Figure 5; Table 7).

Upon subgroup analysis based on region, a nonsignificant reduction was reported in the rate of perinatal mortality in studies conducted in Africa (RR, 0.45, 95% CI, 0.19–1.09, one study, 841 participants) (Ashorn et al., 2015), and Asia (RR, 0.78; 95% CI, 0.47–1.32; one study, 4011 participants) (Mridha et al., 2016). There was no difference between Africa, and Asia for perinatal mortality on FDP (RR, 0.67; 95% CI, 0.41–1.09; two studies, 4852 participants; heterogeneity: *χ* ^2^ *p* = .29, *I* ^2^ = 11%) (Analysis 7.1; Table 8).

**Table 8:** Matrix (food distribution program)

Study (author and year)	Region (Africa/South Asia/South America and Carribean)	Duration of supplementation (whole pregnancy/second trimester/third trimester)	Nutritional status (pregnant women defined based on BMI) undernourished (BMI < 18.5) vs. well nourished (BMI > 18.5)	Location (rural/urban/mixed)
*Food program*				
Ashorn et al. (2015)	East Africa	Whole pregnancy	Well nourished and undernourished	Rural
Frith et al., (2015)	Southeast Asia	Whole pregnancy (from 2nd trimester for control)	Well nourished	Rural
Johnson et al. (2016)	West Africa	Whole pregnancy	Well nourished	Rural
Leroy et al. (2016)	East Africa	Second trimester	Not specified	Not specified
Mridha et al. (2016)	Southeast Asia	Whole pregnancy	Well nourished	Rural

###### Neonatal mortality

One study (Ashorn et al., 2015) reported on neonatal mortality, which showed that FDP may not have had any effect on neonatal mortality (RR, 0.46; 95% CI, 0.20–1.04; one study, 841 participants, low quality on GRADE) (Analysis 6.3; Tables 3 and 7).

##### Child outcomes

###### Infant mortality

One study (Ashorn et al., 2015) reported on infant mortality, which showed that FDP does not probably have any effect on infant mortality (RR, 0.34; 95% CI, 0.01–8.41; one study, 841 participants; moderate quality on GRADE) (Analysis 6.4; Tables 3 and 7).

#### 5.3.6 Secondary outcomes

##### Maternal outcomes

Included studies of FDP failed to report on pre‐eclampsia, placental abruption, overweight, obesity, and iron deficiency anemia.

###### Maternal mortality

Two studies (Ashorn et al., 2015; Mridha et al., 2016) reported on maternal mortality. FDP had no impact on maternal mortality (RR, 0.41; 95% CI, 0.07–2.49; two studies, 4925 participants; heterogeneity: *χ* ^2^ *p* = .79, *I* ^2^ = 0%) (Analysis 6.5).

###### Anemia

One study (Leroy et al., 2016 Table 3) reported on anemia among mothers at 18 and 24 months postpartum. which indicated separate associations for the T24 (mothers received all program benefits during pregnancy until 23.9 months of age of the child; RR, 0.70; 95% CI, 0.60–0.82) and the T18 group (similar to T24 group but received benefits until child was 18 months of age; RR, 0.79; 95% CI, 0.70–0.89). Both of the intervention groups revealed significant reductions in the incidences of anemia in pregnant women (RR, 0.75; 95% CI, 0.67–0.85, one study, 2101 participants) (Analysis 6.6;).

##### Fetal outcomes

Studies on FDP failed to report on congenital anomalies.

##### Newborn outcomes

Studies on FDP failed to report on newborn outcome of macrosomia.

###### Low birth weight

Four studies (Ashorn et al., 2015; Frith et al., 2015; Johnson et al., 2016; Mridha et al., 2016) reported on LBW. FDP showed a significant 8% reduction in the incidence of LBW infants (RR, 0.92; 95% CI, 0.84–1.00, four studies, 5552 participants; heterogeneity: *χ* ^2^ *p* = .85, *I* ^2^ = 0%) (Analysis 6.7).

###### Preterm birth

Three studies (Ashorn et al., 2015; Johnson et al., 2016; Mridha et al., 2016) reported on the incidence of preterm birth. FDP had no impact on preterm birth (RR, 0.92; 95% CI 0.78 to 1.10, three studies, 4,608 participants; heterogeneity: *χ* ^2^ *p* = .56, *I* ^2^ = 0%) (Analysis 6.8).

###### Small‐for‐gestational age

Three studies (Ashorn et al., 2015; Mridha et al., 2016; Johnson et al., 2016) reported on the incidence of SGA. FDP had no impact on reduction of SGA (RR, 0.94; 95% CI, 0.89–1.00; three studies, 4,511 participants; heterogeneity: *χ* ^2^ *p* = .84, *I* ^2^ = 0%) (Analysis 6.9).

###### Birth weight

Three studies (Ashorn et al., 2015; Frith et al., 2015; Mridha et al., 2016) reported on birth weight. FDP showed a significant 46 g increase in birth weight (MD, 46.00 g, 95% CI, 45.10–46.90, three studies, 5,272 participants; heterogeneity: *χ* ^2^ *p* = .83, *I* ^2^ = 0%) (Analysis 6.10).

###### Birth length

Three studies (Ashorn et al., 2015; Frith et al., 2015; Mridha et al., 2016) reported on birth length. FDP showed a significant 0.20 cm increase in mean birth length (MD, 0.20 cm, 95% CI, 0.20–0.20, three studies, 5272 participants; heterogeneity: *χ* ^2^ *p* = .46, *I* ^2^ = 0%) (Analysis 6.11).

###### Head circumference

Two studies (Frith et al., 2015; Mridha et al., 2016) reported on head circumference. FDP had no impact on head circumference (MD, 0.07 cm, 95% CI, −0.22–0.36, two studies, 4490 participants; heterogeneity: *χ* ^2^ *p* = .003, *I* ^2^ = 88%) (Analysis 6.12).

##### Child outcomes

The studies failed to report on the secondary outcomes of developmental outcomes, respiratory disease, allergic diseases, hemoglobin concentration, and iron deficiency anemia in children.

###### Stunting

Two studies (Ashorn et al., 2015; Mridha et al., 2016) reported stunting. FDP showed a significant 18% reduction in risk of stunting (RR, 0.82; 95% CI, 0.71–0.94; two studies, 4166 participants; heterogeneity: *χ* ^2^ *p* = .72, *I* ^2^ = 0%) (Analysis 6.13).

###### Wasting

Two studies (Ashorn et al., 2015; Mridha et al., 2016) reported wasting. FDP showed a significant 13% reduction in risk of wasting (RR, 0.87; 95% CI, 0.78–0.97; two studies, 3883 participants; heterogeneity: *χ* ^2^ *p* = .95, *I* ^2^ = 0%) (Analysis 6.14).

###### Underweight

Two studies (Ashorn et al., 2015; Mridha et al., 2016) reported on underweight. FDP showed no effect on reduction of underweight babies (RR, 0.84; 95% CI, 0.63–1.13; two studies, 4174 participants; heterogeneity: *χ* ^2^ *p* = .19, *I* ^2^ = 41%) (Analysis 6.15).

#### 5.3.7 Comparison 3: Interventions for obesity prevention versus control

Two studies (Aşcı & Rathfisch, 2016; Liu et al., 2017), including 192 participants, targeted on prevention of obesity among pregnant women through dietary interventions and lifestyle modifications.

#### 5.3.8 Primary outcomes

None of the studies have reported any data on the effect of supplementation on primary outcomes, namely maternal BMI, miscarriage, stillbirths, perinatal mortality, neonatal mortality, infant mortality, and under‐five mortality.

#### 5.3.9 Secondary outcomes

##### Maternal outcomes

None of the included obesity prevention trials have reported any data on the effect of supplementation on maternal outcomes, namely maternal mortality, pre‐eclampsia, placental abruption, anemia, overweight, obesity, and iron deficiency anemia.

##### Fetal outcomes

No fetal outcomes were reported by the obesity prevention trials.

##### Newborn outcomes

The obesity prevention studies failed to report preterm birth, LBW, SGA, and head circumference of newborns.

###### Macrosomia

One study (Liu et al., 2017) reported on macrosomia. The counseling on lifestyle modification intervention had no impact on macrosomia (RR, 0.57; 95% CI, 0.18–1.82, random effect, one study, 90 participants) (Analysis 8.1; Table 3).

###### Birth weight

Two trials (Aşcı & Rathfisch, 2016; Liu et al., 2017) reported on mean birth weight. The counseling on lifestyle modification intervention showed a significant reduction in mean birthweight (MD, −195.57; 95% CI, −349.46 to −41.68, random effect, two studies,180 participants; heterogeneity: *χ* ^2^ *p* = .84, *I* ^2^ = 0%) (Analysis 8.2).

###### Birth length

One study (Aşcı & Rathfisch, 2016) reported on birth length. The interventions to prevent obesity had no impact on mean birth length (MD, −0.36; 95% CI, −1.12–0.40, random effect, one study, 90 participants) (Analysis 8.3; Table 6).

##### Child outcomes

No child outcomes were reported by the obesity prevention trials.

#### 5.3.10 Sensitivity analysis

##### Allocation concealment

###### BEP supplementation

Sensitivity analysis was conducted on BEP trials prejudicing for unclear risk of selection bias. We conducted sensitivity analysis on primary outcomes for allocation concealment by removing Mora et al. (1978b) due to high risk of allocation concealment. No significant change was observed in stillbirths after removing Mora et al. (1978b).

###### Food distribution program

Sensitivity analysis was conducted in FDP trials prejudicing for unclear risk of selection bias and none of the FDP trials were excluded as a result.

###### Interventions for obesity prevention

We were unable to conduct sensitivity analysis on trials of obesity prevention since no primary outcomes of our interest were reported in the included studies.

##### Attrition bias

###### BEP supplementation

All BEP trials reporting primary outcomes reported attrition rates below 10%, clearing them of attrition bias. Thus sensitivity analysis was not performed on BEP trials to study the impact of attrition bias.

###### Food distribution program

We excluded Mridha et al. (2016) while performing sensitivity analysis to consider the impact of attrition bias, and no significant shifts were experienced in the overall findings of perinatal mortality.

###### Interventions for obesity prevention

Sensitivity analysis could not be performed for any obesity prevention outcomes in any capacity as the trials did not investigate any of the primary outcomes of the study.

##### **I**mputed inter correlation coefficients

###### BEP supplementation

In our cluster‐adjustment methods sensitivity analysis, ICC applied was derived from Leroy et al. (2016) (i.e., 0.006 ICC) for Ceesay et al. (1997). The subsequent exclusion of Ceesay et al. (1997) resulted in a significant change in the results where the results of stillbirth changed from (RR, 0.39; 95% CI, 0.19–0.80, three studies, 1913 participants; heterogeneity: *χ* ^2^ *p* = .80, *I* ^2^ = 0%) to (RR, 0.27; 95% CI, 0.07–1.05, two studies, 467 participants; heterogeneity: *χ* ^2^ *p* = .87, *I* ^2^ = 0%).

###### Food distribution program

For FDP studies, no significant shift was observed in perinatal mortality after removing Mridha et al. (2016).

###### Interventions for obesity prevention

Sensitivity analysis could not be performed for obesity prevention outcomes in any capacity as the trials did not investigate any of the primary outcomes of the study.

## 6 DISCUSSION

### 6.1 Summary of main results

We included a total of 15 trials involving healthy pregnant women from LMICs. Eight studies evaluated the impact of BEP supplementation (25% of energy provided by protein), five on FDP, and two on obesity prevention program. This review summarized the current evidence on the effect of dietary interventions during pregnancy on maternal, neonatal, and child outcomes.

BEP supplementation may reduce incidence of stillbirths by 61%, perinatal mortality by 5%, LBW infants by 40%, SGA by 29%, and increased birth weight by 107.28 g. An increase of 107.28 g of birthweight is clinically significant in the countries where the intervention has been provided. BEP supplementation had no effect on miscarriage, neonatal mortality, infant mortality, preterm birth, birth length, and head circumference. Sensitivity analysis was conducted for primary outcomes on allocation concealment, attrition bias, and ICC. The subsequent exclusion of a study for sensitivity analysis of allocation concealment did not result in a significant change of outcome of stillbirths, however, sensitivity analysis for ICC resulted in a significant change in results. There was no effect of attrition bias on the sensitivity analysis.

FDPs showed an improvement in mean birth weight by 46 g, birth length by 0.20 cm, and reduction in stunting by 18%, and wasting by 13%. No improvements were observed for miscarriages, maternal mortality, perinatal mortality, neonatal mortality, infant mortality, LBW, preterm birth, SGA babies, mean head circumference, and rates of underweight babies. For sensitivity analysis, all trials were unclear for selection bias therefore none of them were excluded. Sensitivity analysis for attrition bias and ICC resulted in no significant change in the findings of perinatal mortality.

Trials on counseling on lifestyle modification for pregnant women on obesity prevention failed to report on the primary outcomes but did show a significant 195.57 g reduction in mean birthweight and had no effect on mean birth length, and macrosomia. A sensitivity analysis could not be conducted for this comparison.

### 6.2 Overall completeness and applicability of evidence

An extensive search was done to locate relevant papers on multiple search engines outlined. The reference lists of included studies and reviews were searched as well. Data was extracted and quality was assessed in duplicate. Differences were discussed and resolved between the authors, and where needed a fourth author was consulted. The quality of selected outcomes was also assessed using GRADE analysis.

This review summarized findings from 15 studies. These studies were conducted in LMICs, particularly in Africa, Asia, Central and South America. BEP supplementation appeared to be effective in improving rates of stillbirth, perinatal mortality, LBW, SGA, and birth weight, whereas FDP was found to improve birth weight, birth length, stunting, and wasting. The obesity prevention studies were few in number but demonstrated a significant reduction in mean birth weight. We also conducted a sensitivity analyses to assess the effects by removing trials that imputed inter‐correlation coefficients through different ways; and the results show a significant change in the estimates. However, the included studies were conducted among healthy pregnant women in LMICs, thus these results cannot be generalized in high income countries.

### 6.3 Quality of the evidence

We judged the quality of the evidence of the individual studies by utilizing ROB assessment tool for RCTs and cRCTs, and EPOC for quasi experimental trials. GRADE methodology was used to assess the quality of evidence for the primary outcomes. We also conducted a sensitivity analysis for outcomes with high risk of allocation concealment, attrition bias, and imputed inter‐correlation coefficients, and analysed the change in estimate.

In BEP, the overall quality of evidence for infant mortality was moderate, however, stillbirths, perinatal mortality, neonatal mortality, LBW, and preterm birth was low to very low due to down grading for imprecision and ROB. For FDP, the overall quality of evidence for all outcomes ranged from moderate to low due to downgrading for imprecision and ROB. We did not perform GRADE analysis for interventions for obesity prevention, since none of the included studies reported on the primary outcomes of our interest.

### 6.4 Potential biases in the review process

Efforts were made to reduce all potential sources of bias. We conducted an extensive electronic search on the pre‐specified databases. The methodology was followed religiously as per the protocol (Lassi et al., 2019). We included 15 studies out of which eight were on BEP supplementation. To improve the quality of the study, we also performed sensitivity analysis. Also, we did not conduct a funnel plot analysis as it would have been inconclusive due to the small number of studies (each intervention/outcome comparison had fewer than 10 studies).

### 6.5 Agreements and disagreements with other studies or reviews

Our findings on BEP supplementation are in agreement with Ota et al. (2015), which included 11 studies investigating BEP; seven studies from LMICs and four from high‐income countries (HICs). Ota et al. (2015) reported reductions in stillbirth, and SGA babies with BEP supplementation. Our pooled analyses also showed reduction in rates of stillbirths, LBW, SGA and improved birth weight.

A systematic review by Imdad and Bhutta (2011) also assessed the effectiveness of BEP supplementation and included six studies from LMICs and five from HICs. Our review reported reductions in stillbirths and increases in birth weight. These findings are in concordance with Imdad and Bhutta (2011). Kramer and Kakuma (2003) also reported similar findings, however it included studies from HICs. Though our review reported similar impacts as earlier reviews, direct comparison with Imdad and Bhutta (2011) and Ota et al. (2015) is difficult as those reviews included studies from HICs, whereas, our review only included eight studies on BEP from LMICs.

We were unable to find systematic reviews on FDP due to the lack of a definition of a FDP. Also no such review was conducted in LMIC settings to be compared with our review.

Reviews investigating obesity prevention programs in recent time were not particularly successful with Furber et al. (2013) netting no studies. One comparable review on mitigating the excessive weight gain during pregnancy by Flynn et al. (2016) reported outcomes pertaining to the state of the participants’ diabetes mellitus. This was not wholly comparable to our review's focus on maternal mortality, child mortality, child growth, and developmental outcomes. This makes our review the first to review the evidence for dietary interventions for obesity prevention in pregnancy.

## 7 AUTHORS' CONCLUSIONS

### 7.1 Implications for practice

Our review highlights improvement in maternal, neonatal, infant, and child outcomes through BEP supplementation and FDP during pregnancy. The results from the review suggests that BEP supplementation was effective in improving rates of stillbirth, perinatal mortality, LBW, SGA, and birth weight. BEP supplementation showed a clinical significance in increase in birthweight, when supplemented to pregnant women during pregnancy. This review also showed that FDPs have many benefits to longer term anthropometric outcomes and early life mortality. The lack of standardization of the term “food supplementation” indicated in this review put the generalizability of the intervention in question. The studies on obesity prevention demonstrated improvement in the rates of macrosomia upon relevant dietary and lifestyle counseling. However, we are uncertain of the role of BEP, FDP, and dietary intervention for obesity prevention due to limited, and low to very low quality of evidence. The low number of studies, small number of participants, and setting varied in the included studies, thus the findings of the review has limited applicability and generalisibility. Thus, we need a more robust evidence based on BEP supplementation, FDP, and obesity prevention among pregnant women through dietary interventions to confidently understand maternal, neonatal and child outcomes.

### 7.2 Implications for research

We found limited data on effectiveness of BEP supplementation, FDP, and dietary intervention for prevention of obesity among pregnant women. Further good quality studies are required to evaluate the role and potential effect of BEP supplementation, FDP, and dietary intervention for prevention of obesity on maternal, neonatal, and child outcomes. Moreover, the effect of intervention in various population groups should also be explored for generalizability of results, and to draw authentic conclusions.

## CONTRIBUTIONS OF AUTHORS

Aamer Imdad, Deepika Ranjit, Gamael Saint Saint Surin, Rehana A. Salam, Zulfiqar A. Bhutta, and Zohra S. Lassi wrote the protocol. Search strategy and search run was conducted by Zahra A. Padhani. Title/abstract screening was done by Fahad Rind and Zahra A. Padhani. Full text screening and data extraction was done by Amna Rabbani, Fahad Rind, and Zahra A. Padhani. Analysis and write‐up was done by Amna Rabbani, Fahad Rind, and Zahra A. Padhani. Study was reviewed by Zohra S. Lassi.

## DECLARATIONS OF INTEREST

The authors declare no conflicts of interest arising from financial or researcher interest.

## DIFFERENCES BETWEEN PROTOCOL AND REVIEW

In the protocol we planned to analyse GRADE on primary outcomes inclusive of two secondary outcomes, that is, low birthweight and preterm birth. Also one of our primary outcome 'miscarriages' was missing from the list of GRADE. Thus, in this review we have analysed only primary outcomes, and have also included miscarriage on our list. We have not analysed GRADE on low birth weight and preterm birth.

## CHARACTERISTICS OF STUDIES

### Characteristics of included studies

Ashorn et al. (2015)

**Table:** 

**Methods**	**Study design:** Randomized controlled trial
	**Unit of randomization:** Individually randomised
	**Type of study:** Food Distribution Program
**Participants**	**Location/setting:** One public district hospital in Mangochi, one semi‐private hospital in Malindi and 2 Public health centres in Lungwena and Namwera in Mangochi District of Malawi
	**Population:** Not specified
	**Sample size:** 1391
	**Drop outs/withdrawal:** 84 IFA (*n* = 26), LNS (*n* = 26)
	**Socio‐demographics**
	**Mean (*SD*) age:** LNS: 25 (6), IFA: 25 (6)
	**Occupation:** Farming and fishing
	**Race:** Not specified
	**Education:** Maternal education, completed years (mean)
	IFA: 3.9
	LNS: 4.1
	**Family income:** Not specified
	**Inclusion criteria:**
	Pregnant women who came for antenatal care at any of the study clinics during the enrolment period and met the following inclusion criteria: ultrasound confirmed pregnancy of no more than 20 completed gestational weeks, residence in the defined catchment area, availability during the period of the study and signed or thumb‐printed informed consent
	**Exclusion criteria:**
	Age younger than 15 years, need for frequent medical attention due to a chronic health condition, diagnosed asthma treated with regular medication, severe illness warranting hospital referral, history of allergy toward peanuts, history of anaphylaxis or serious allergic reaction to any substance, requiring emergency medical care, pregnancy complications evident at enrolment visit (moderate to severe edema, blood haemoglobin concentration <50 g/L, systolic blood pressure >160 mmHg or diastolic blood pressure >100 mmHg), earlier participation in the iLiNS‐DYAD‐M trial (during a previous pregnancy) or concurrent participation in any other clinical trial
**Interventions**	**Intervention (sample size):**
	LNS: Tailor‐made SQ‐LNS from enrolment to 6 months postpartum. Daily dose of 20 g to provide the same micronutrients as MMN, 4 additional minerals (calcium, phosphorus, potassium, magnesium), protein and fat providing 118 kcal of energy. Raw ingredients for LNS included soybean oil, dried skimmed milk, peanut paste, mineral and vitamin mix, and sugar. Intervention was delivered through data collectors (*n* = 462)
	**Control (sample size):**
	IFA: received standard antenatal care including supplementation of iron 60 mg, folic acid 400 µg from enrolment to delivery. It was delivered through data collectors (*n* = 463)
	**Concomitant interventions:**
	Received intermittent preventive malaria treatment, that is, 2 doses of intermittent preventive malaria treatment with sulphadoxine‐pyrimethamine (3 tablets of 500 mg sulphadoxine and 25 mg pyrimethamine orally). One sulphadoxine‐pyrimethamine dose was given at enrolment and the other between weeks 28 and 34 of gestation
	Health facility nurses gave pretest HIV counselling and tested for HIV infection in all participants, except those who opted out or were already known to be HIV infected, by using a whole‐blood antibody rapid test. LNS (*n* = 462)
	**Training:**
	Study physicians trained health providers at all the known private and public health facilities in the area to identify the study participants from their iLiNS identification cards and to record information on any nonscheduled visits on structured data collection forms that were collected and reviewed by the study team on a weekly basis
	**Follow‐up:**
	Study coordinators invited the participants for follow‐up at the study clinic twice during pregnancy (at 32 and 36 gestational weeks) and once after birth, at 1–2 weeks after delivery and at 6 months; post‐natal follow‐up done till 6 weeks after delivery
	Participants were also provided with mobile phones and airtime so that they could immediately inform the study coordinators about the deliveries (which took place outside the clinics). Upon notification the coordinator visited the site of delivery for interview and infant measurements
**Outcomes**	**Primary outcomes:**
	Perinatal mortality
	Neonatal mortality
	Infant mortality
	**Secondary outcomes:**
	Matenal mortality
	Low birthweight
	Preterm birth
	Small‐for‐gestational age
	Birth weight
	Birth length
	Stunting
	Underweight
	**Timing of outcome assessment:** Within 6 weeks of birth
**Notes**	**Study start date:** February 2011
	**Study end date:** August 2012
	**Time period:** 28 months
	**Study country:** Malawi
	**Study limitations:**
	Large number of missing data, delays in anthropometric measurements of some participants, temporary discontinuation of LNS distribution and inability to observe consumption of study supplements. However, the smaller sample size than originally intended (due to budget reduction) limited the statistical power of the study. In preliminary analyses from the current study population, maternal malaria, HIV infection, and inflammatory response appeared associated with adverse pregnancy outcomes and also seemed to modify some of the intervention effects on them
	**Funding source:**
	Supported in part by a grant to the University of California, Davis, from the Bill & Melinda Gates Foundation, with additional funding from the Office of Health, Infectious Diseases, and Nutrition, Bureau for Global Health, US Agency for International Development (USAID) under terms of Cooperative Agreement No. AID‐OAA‐A‐12‐00005, through the Food and Nutrition Technical Assistance III Project (FANTA), managed by FHI 360. For data management and statistical analysis, the team received additional support in grants from the Academy of Finland (grant 252075) and the Medical Research Fund of Tampere University Hospital (grant 9M004). YBC was supported by the Singapore Ministry of Health's National Medical Research Council under its Clinician Scientist Award
	**Conflict of Interest:** Not specified
	**Comments:**
	There were 3 arms in the study: LNS (as intervention) and IFA and MMN as control. For our study we took IFA as our control

Risk of bias table

**Table:** 

Bias	Authors' judgement	Support for judgement
Random sequence generation (selection bias)	Low risk	**Quote:** “A study statistician not involved in data collection generated 4 randomisation code lists in blocks of 9 (one list for each of the 4 enrolment sites)”
		**Comment:** Adequately done
Allocation concealment (selection bias)	Low risk	**Quote:** “The researcher sealed the slips into individual opaque randomisation envelopes, marked each envelope with the trial name and an individual participant number”
		**Comment:** Adequately done
Blinding of participants and personnel (performance bias)	Low risk	**Quote:** “IFA and MMN interventions were provided by using double‐masked procedures—that is, the capsules looked identical, and neither the participants nor the research team members were aware of the nutrient contents of the supplement capsules. For the LNS group, we used single‐masked procedures”
		**Comment:** Adquately done
Blinding of outcome assessment (detection bias)	Low risk	**Quote:** “Researchers responsible for the data cleaning remained blind to the trial code until the database was fully cleaned”
		**Comment:** Adequately done
Incomplete outcome data (attrition bias)	Low risk	**Comment:** IFA: 26/463 × 100 = 5.1% lost to follow‐up
		LNS: 26/462 × 100 = 5.6% lost to follow‐up
Selective reporting (reporting bias)	High risk	**Comment:** Outcomes mentioned in the protocol are not completely reported in the study (NCT01239693)
Other bias	Low risk	**Comment:** No other potential sources of bias reported
Was the allocation sequence adequately generated?	Unclear risk	
Was the allocation adequately concealed?	Unclear risk	
Were baseline outcome measurements similar?	Unclear risk	
Were baseline characteristics similar?	Unclear risk	
Were incomplete outcome data adequately addressed?	Unclear risk	
Was knowledge of the allocated intervention adequately prevented during the study?	Unclear risk	
Was the study adequately protected against contamination?	Unclear risk	
Was the study free from selective outcome reporting?	Unclear risk	
Was the study free from other risk of bias?	Unclear risk	

Aşcı and Rathfisch (2016)

**Table:** 

**Methods**	**Study design:** Randomized controlled trial
	**Unit of randomization:** Individually randomised
	**Type of study:** Obesity prevention
**Participants**	**Location/setting:** Family healthcare centre, Istanbul, Turkey
	**Population:** 21 000
	**Sample size:** 102
	**Drop outs/withdrawal:** 12 (6 in intervention group, 6 from control) due to miscarriage(2), preterm birth (2) relocation (3) and 5 discontinuations
	**Socio‐demographics**
	**Mean (*SD*) age/age range:** Intervention: 24.31 (4.22) Control: 24.28 (4.15)
	**Occupation:** Not specified
	**Race:** Not specified
	**Education:**
	Intervention: 7.6 ± 3.2
	Control: 6.6 ± 2.8
	**Family income:**
	Working women *n* (%)
	Intervention:
	Low: 15 (33.3)
	Middle: 30 (66.7)
	Control:
	Low: 11 (24.4)
	Middle: 34 (75.6)
	**Inclusion criteria:**
	Pregnant women aged over 18, who had no health problem, did not intend to lose weight in prepregnancy period, got pregnant in natural ways for two times at most, and were pregnant for a period of 3 months or less, were included in the study
	**Exclusion criteria**: Pregnancy complications, not coming to regular follow‐ups
**Interventions**	**Intervention (sample size):**
	Lifestyle intervention for whole pregnancy till 6 weeks postpartum. 4 meetings were held with women regarding healthy lifestyle, nutrition, exercise, and weight follow‐up. Patients were given a card on which weight, height, and gestational weight gain were recorded. The women were provided with praise and those that were not able to meet objectives had their short comings reviewed. Women who could not reach their objectives were given more intensive consultancy (repetition of basic nutrition and physical activity recommendations, reviewing individual objectives, and supportive phone consultancy) was provided. Counselling and behavioral coaching was personalized according to the barriers for individuals. Meetings were supplemented with 15 minute health training and brochures were delivered
	It was delivered by the first author of the study (nurse) (*n* = 51)
	**Control (sample size):**
	Standard of care. Consultancies mostly consist of subjects such as pregnancy complaints, scope of antenatal care, tests to be performed, birth, post‐partum period, and circumstances that might pose danger during pregnancy, There is no standard training and consultancy. Women in the standard care group (control) are followed up at least four times by midwives or nurses. Every trip weights are measured. Consultancy is granted for pregnancy complaints, tests, birth and postpartum period but lifestyle advice is not granted. Duration was from 12th week pregnancy till 6 weeks postpartum. It was delivered by nurses and midwives (*n* = 51)
	**Training:** Not specified
	**Follow‐up:**
	Intervention group: In weeks 12–15, the focus was on healthy life and health practices. In weeks 16–18, the focus was on physical activity and exercises. In weeks 20–24, interviews regarding nutrition were held, that is, meal frequency, size, content. On week 37, only weights were followed up and target achievement was reviewed and the intervention was ended. At 6 weeks postpartum obstetric and neonatal outcomes were measured
	Control Group: Women are generally followed up by at least four times by midwives or nurses in standard care. In every follow‐up, weights of women are measured
	Duration: From 12th week of pregnancy to 6 weeks postpartum
**Outcomes**	**Primary outcomes:** None
	**Secondary outcomes:**
	Birth weight
	Birth length
	**Timing of outcome assessment:** 6 week postartum
**Notes**	**Study start date:** June 2011
	**Study end date:** July 2012
	**Time period:** 13 months
	**Study country:** Turkey
	**Study limitations:**
	Participation in the study was stopped as soon as the sample size determined by the power analysis was obtained without taking case losses into consideration due to the time constraints for the completion of the study. This situation caused the study to be completed with a smaller sample size than planned. Intervention was applied by the same person in the study (first author of this study who was the nurse officially rendering services in the centre on the dates that the study was conducted) within official working hours. From these aspects, the intervention was strong in terms of controlling the contents of consultancy for each participant and “realistic” in terms of applicability by nurses and midwives. However, the fact that the study was conducted in only one centre and the sample group was limited to healthy pregnant women who did not intend to lose weight and had less than two pregnancies even if they were selected randomly is not sufficient for generalizing the results of the study
	**BMI pre‐pregnancy:**
	Intervention: 23.86 ± 4.10
	Control: 22.82 ± 3.93
	**Funding Source:** Not specified
	**Conflict of Interest:** None

Risk of bias table

**Table:** 

Bias	Authors' judgement	Support for judgement
Random sequence generation (selection bias)	Low risk	**Quote:** “Women were divided into randomised groups by a staff who was not involved in this study, by drawing lots”
		**Comment:** Adequately done
Allocation concealment (selection bias)	Unclear risk	**Comment:** Insufficient information to permit judgement
Blinding of participants and personnel (performance bias)	Low risk	**Quote:** “Participants were blind about which group they were involved in and the evaluated study outcomes”
		**Comment:** Adequately done
Blinding of outcome assessment (detection bias)	Unclear risk	**Comment:** Insufficient information to permit judgement
Incomplete outcome data (attrition bias)	High risk	**Comment:** Intervention: 6/51 × 100 = 11.7% lost to follow‐up
		Control: 6/51 × 100 = 11.7% lost of follow‐up
Selective reporting (reporting bias)	Unclear risk	**Comment:** Published protocol not found. The outcomes specified in the methodology section have been reported in the results section
Other bias	Low risk	**Comment:** No other potential sources of bias reported
Was the allocation sequence adequately generated?	Unclear risk	
Was the allocation adequately concealed?	Unclear risk	
Were baseline outcome measurements similar?	Unclear risk	
Were baseline characteristics similar?	Unclear risk	
Were incomplete outcome data adequately addressed?	Unclear risk	
Was knowledge of the allocated intervention adequately prevented during the study?	Unclear risk	
Was the study adequately protected against contamination?	Unclear risk	
Was the study free from selective outcome reporting?	Unclear risk	
Was the study free from other risk of bias?	Unclear risk	

Ceesay et al. (1997)

**Table:** 

**Methods**	**Study Design:** Randomized controlled trial
	**Unit of Randomization:** Individually randomised
	**Type of study:** Balanced Energy Protein
**Participants**	**Location/Setting:** Farming communities, West Kiang region, Gambia
	**Population:** 12,000
	**Sample size:** 2047
	**Drop outs/withdrawal:** Not specified
	**Socio‐demographics**
	**Mean (*SD*) age/age range:** Intervention: 24 (6.2) Control: 23.7 (6.4)/15–45 years
	**Occupation:** Farming
	**Race:** Not specified
	**Education:** Not specified
	**Family income:** Not specified
	**Inclusion criteria:** Women of reproductive age
	**Exclusion criteria:** Not specified
**Interventions**	**Intervention (sample size):**
	Frequency of intervention was the daily intake of two biscuits
	Composition: The biscuits were made of roasted groundnuts, rice flour, sugar, groundnut oil and they provided a maximum intake of 4250 kJ energy, 22 g protein, 56 g fat, 47 mg Calcium and 1.8 mg Iron. The biscuits were made at a single village. Duration of intervention was about 20 weeks (82 days). Supplementation was delivered by two birth attendants in each village (*n* = 1010)
	**Control (sample size):**
	Pregant females who received supplementation with high energy biscuits after delivery. Supplentation was delivered by two birth attendants in each village (*n* = 1037)
	**Concomitant interventions:**
	Both groups received:
	1. Routine antenatal care (interview; abdominal palpation; blood pressure and haemoglobin and urine protein concentrations; treatment or referral as indicated) from midwives in a mobile clinic that visited each village twice monthly 2. Iron and folate supplements (according to haemoglobin concentration) 3. Tetanus toxoid to not previously protected women 4. prophylactic dose of chloroquine (in hungry season)
	**Training:**
	The field workers were trained in anthropometric techniques and Parkin scoring in the delivery wards of the Gambia's main hospital
	**Follow‐up:**
	Maternal height (baseline), maternal weight (fortnightly), parity, birth weight, birth length, head circumference, and gestational age were assessed by eight field workers within 48 h of delivery
	Stillbirths were recorded.
	Infant deaths were recorded by a governmentappointed village recorder and cross checked by field workers when each infant became due for follow‐up anthropometry
**Outcomes**	**Primary outcome:**
	Stillbirth
	Perinatal mortality
	Neonatal mortality
	Infant mortality
	**Secondary outcome:**
	Low birthweight
	Birth weight
	Birth length
	Head circumference
	**Timing of outcome assessment:** Within 48 h of delivery
**Notes**	**Study start date:** July 1989
	**Study end date:** October 1994
	**Time period:** 5 years
	**Study country:** Gambia, West Africa
	**Study limitations:**
	Had inadequate sample power to test the prediction of a 37% reduction in neonatal mortality (equivalent to an odds ratio of 0.60)
	**Funding source:**
	Medical Research Council, Overseas Development Administration, and Nestlé Foundation
	**Conflict of interest:** None

Risk of bias table

**Table:** 

Bias	Authors' judgement	Support for judgement
Random sequence generation (selection bias)	Low risk	**Quote:** “Using a stratified design according to village size”
		**Comment:** Adequately done
Allocation concealment (selection bias)	Unclear risk	**Comment:** Insufficient information to permit judgement
Blinding of participants and personnel (performance bias)	Unclear risk	**Comment:** Insufficient information to permit judgement
Blinding of outcome assessment (detection bias)	Unclear risk	**Comment:** Insufficient information to permit judgement
Incomplete outcome data (attrition bias)	Low risk	**Quote:** “Over 95% agreed and remained in the trial throughout”
Selective reporting (reporting bias)	Unclear risk	**Comment:** Published protocol not found.The outcomes specified in the methodology section have been reported in the results section
Other bias	Low risk	**Comment:** No other potential sources of bias reported
Was the allocation sequence adequately generated?	Unclear risk	
Was the allocation adequately concealed?	Unclear risk	
Were baseline outcome measurements similar?	Unclear risk	
Were baseline characteristics similar?	Unclear risk	
Were incomplete outcome data adequately addressed?	Unclear risk	
Was knowledge of the allocated intervention adequately prevented during the study?	Unclear risk	
Was the study adequately protected against contamination?	Unclear risk	
Was the study free from selective outcome reporting?	Unclear risk	
Was the study free from other risk of bias?	Unclear risk	

Dwarkanath et al. (2016)

**Table:** 

**Methods**	**Study design:** Randomized controlled trial
	**Unit of randomization:** Individually randomised
	**Type of study:** Balanced Energy Protein
**Participants**	**Location/setting:** Metabolic ward of St. John's Research Institute, Bangalore, India
	**Population:** Not specified
	**Sample size:** 24
	**Drop outs/withdrawal:** None
	**Socio‐demographics**
	**Mean (*SD*) age:** Total: 22.9 (3.24), Intervention: 23.7 (4.1), Control: 22.2 (1.99)
	**Occupation:** Not specified
	**Race:** Not specified
	**Education:** Not specified
	**Family income:** Not specified
	**Inclusion criteria:**
	Only pregnant women from the same dwelling area and similar socioeconomic background were invited to join the study. Pregnant women at <13 weeks of gestation with low BMIs (≤18.5) and normal plasma folate and vitamin B‐12 status were identified and invited
	**Exclusion criteria:**
	Women with multiple pregnancies, those with a clinical diagnosis of chronic illness (e.g., diabetes mellitus, hypertension, cardiac disease, thyroid disease, or epilepsy), and those who tested positive for hepatitis B surface antigen, HIV, or syphilis were excluded
**Interventions**	**Intervention (sample size):**
	In addition to their normal habitual diet, the supplemented group received a daily dietary supplement of 300 kcal/day energy and 15 g protein/day (i.e., 20% of energy from protein), provided as 3 small, round granola‐type treats, called “*ladoos*“ in the local language, and made of crushed roasted peanuts, puffed rice, skimmed milk, clarified butter, and unrefined sugar from 1st trimester till delivery. Intervention was delivered by a social worker (*n* = 12)
	**Control (sample size):**
	No supplementation. The control group continued to consume their habitual diet. It was delivered by a social worker
	(*n* = 12)
	**Training:** Not specified
	**Follow‐up:**
	Protein kinetics and serine and glycine fluxes were measured by using standard stable isotope tracer methods in the fasting and postprandial state at end of 1st and beginning of 3rd trimester. 24‐h food recalls were obtained three times during each trimester of pregnancy
**Outcomes**	**Primary outcomes:** Miscarriage
	**Secondary outcomes:**
	Low birthweight
	Preterm birth
	Small‐for‐gestational age
	Birth weight
	Birth length
	**Timing of outcome assessment:** At birth
**Notes**	**Study start date:** Not specified
	**Study end date:** Not specified
	**Time period:** Not specified
	**Study country:** India
	**Study limitations:** The sample size was relatively small.
	**Funding source:**
	Supported by federal funds from the USDA, Agricultural Research Service, under Cooperative Agreement 58‐6250‐6001
	**Conflict of interest:** None

Risk of bias table

**Table:** 

Bias	Authors' judgement	Support for judgement
Random sequence generation (selection bias)	Unclear risk	**Quote:** “Enrolled subjects were randomly assigned to either a supplemented or non supplemented group”**Comment:** Insufficient information to determine level of bias
Allocation concealment (selection bias)	Unclear risk	**Comment:** Insufficient information to permit judgement
Blinding of participants and personnel (performance bias)	Unclear risk	**Comment**: Insufficient information to permit judgement
Blinding of outcome assessment (detection bias)	Unclear risk	**Comment:** Insufficient information to permit judgement
Incomplete outcome data (attrition bias)	Low risk	**Quote:** “One woman in each group lost her fetus before 30 weeks of gestation and one woman in the supplemented group missed her infusion date because of travel. However, she continued participating in the study by taking her dietary supplement and her pregnancy outcome data are included”
Selective reporting (reporting bias)	Unclear risk	**Comment:** Published protocol not found. The outcomes specified in the methodology section have been reported in the results section
Other bias	Low risk	**Comment:** No other potential sources of bias reported
Was the allocation sequence adequately generated?	Unclear risk	
Was the allocation adequately concealed?	Unclear risk	
Were baseline outcome measurements similar?	Unclear risk	
Were baseline characteristics similar?	Unclear risk	
Were incomplete outcome data adequately addressed?	Unclear risk	
Was knowledge of the allocated intervention adequately prevented during the study?	Unclear risk	
Was the study adequately protected against contamination?	Unclear risk	
Was the study free from selective outcome reporting?	Unclear risk	
Was the study free from other risk of bias?	Unclear risk	

Frith et al. (2015)

**Table:** 

**Methods**	**Study design:** Randomized controlled trial
	**Unit of randomization:** Individually randomised
	**Type of study:** Food Distibution Program
**Participants**	**Location/setting:** Matlab, Chandipur district, Bangladesh.
	**Population:** Not specified
	**Sample size:** 1041
	**Drop outs/withdrawal:** 259
	**Socio‐demographics**
	**Mean (*SD*) age:** Total: 26.6 (5.7), Intervention: 26.7 (5.6) Control: 26.5 (5.8)
	**Occupation:** Not specified
	**Race:** Not specified
	**Education:** Not specified
	**Family income:** Not specified
	**Inclusion criteria:**
	Women (aged 14–50 years) in the study area (Matlab) with pregnancy confirmed by urine test and ultrasound with gestational age <14 weeks. The sample for this sub study was recruited from all eligible MINIMat participants who gave birth between June 2003 and March 2004
	**Exclusion criteria:** Not specified
**Interventions**	**Intervention (sample size):**
	Early Supplementation:The supplement contained rice, lentils, molasses and oil, contained 2.5 MJ per day, 6 days a week (29% of recommended energy intake), 25% of which was vegetable protein from 9 weeks of pregnancy to birth to 6 months postpartum. Intervention was delivered by community nutrition educators (*n* = 533)
	**Control (sample size):**
	Usual start of joining of government food supplementation. The supplement contained rice, lentils, molasses and oil, contained 2.5 MJ per day, 6 days a week (29% of recommended energy intake), 25% of which was vegetable protein. Duration of supplementation was from 20 weeks of pregnancy to birth to 6 months post partum. Intervention was delivered by community nutrition educators (*n* = 508)
	**Concomitant Interventions**:
	Each participant was also assigned to receive one of two counselling protocols from 30 weeks of gestation until 6 months after giving birth as follows: either usual health messages alone or usual health messages with exclusive breastfeeding counselling. Beginning at 14 weeks of gestation until 3 months post‐partum, each participant received one of three daily micronutrient supplements of either 60 mg or 30 mg of iron with 400 mg folic acid or multiple micronutrients
	**Training:**
	Community nutrition educators were trained by the implementing organisation, BRAC, to deliver nutrition education messages and to encourage women to consume food packets completely on site
	**Follow‐up:**
	Daily follow‐ups were conducted for salivary cortisol level monitoring but that is not relevant to our review. Measured cortisol from 28 to 32 weeks of gestation. Birth weight measured between one day of birth
**Outcomes**	**Primary outcomes:** None
	**Secondary outcomes:**
	Low birthweight
	Birth weight
	Birth length
	Head circumference
	**Timing of outcome assessment:** Within one day of birth
**Notes**	**Study start date:** June 2003
	**Study end date:** March 2004
	**Time period:** 9 months
	**Study country:** Bangladesh
	**Study limitations:**
	Pregnant women could have partially or fully substituted the food packets for food at home, although the nutritional quality of food supplement may have been better than the food at home
	**Funding source:**
	Supported by the American Institute of Bangladesh Studies, Cornell Einaudi Center for International Studies, and NIH (training grant 5T32DK07158).The Maternal Infant Nutritional Interventions Matlab (MINIMat) research study was funded by United Nations Children's Fund, Swedish International Development Cooperation Agency (SIDA), UK Medical Research Council, Swedish Research Council, Department of International Development (DFID), International Centre for Diarrhoeal Disease Research, Bangladesh (ICDDR, B), Global Health Research Fund‐Japan, Child Health and Nutrition Research Initiative, Uppsala University and the United States Agency for International Development. Donors who provided unrestricted support to the Centre's research efforts: Australian International Development Agency, Government of Bangladesh, Canadian International Development Agency, Government of Japan, Government of Netherlands, SIDA, Swiss Development Cooperation and DFID
	**Conflict of interest:** None

Risk of bias table

**Table:** 

Bias	Authors' judgement	Support for judgement
Random sequence generation (selection bias)	Low risk	**Quote:** “MINIMat was a randomised controlled field trial with a 2×3×2 factorial design”
		**Comment:** Adequately done
Allocation concealment (selection bias)	Unclear risk	**Comment:** Insufficient information to permit judgement
Blinding of participants and personnel (performance bias)	Unclear risk	**Comment:** Insufficient information to permit judgement
Blinding of outcome assessment (detection bias)	Unclear risk	**Comment:** Insufficient information to permit judgement
Incomplete outcome data (attrition bias)	High risk	**Quote:** “One hundred and thirteen women had temporarily moved to another location outside of Matlab for the pregnancy and birth; 11 had permanently moved; 20 were absent from their homes and no one reported where they had gone; two women refused to participate; two women had measles; and 111 had either miscarried, dropped out”
		**Comment:** 259/1300 × 100 = 19.9% lost to follow‐up
Selective reporting (reporting bias)	Low risk	**Comment:** Outcomes mentioned in the protocol were reported (ISRCTN16581394)
Other bias	Low risk	**Comment:** No other potential sources of bias reported
Was the allocation sequence adequately generated?	Unclear risk	
Was the allocation adequately concealed?	Unclear risk	
Were baseline outcome measurements similar?	Unclear risk	
Were baseline characteristics similar?	Unclear risk	
Were incomplete outcome data adequately addressed?	Unclear risk	
Was knowledge of the allocated intervention adequately prevented during the study?	Unclear risk	
Was the study adequately protected against contamination?	Unclear risk	
Was the study free from selective outcome reporting?	Unclear risk	
Was the study free from other risk of bias?	Unclear risk	

Girija et al. (1984)

**Table:** 

**Methods**	**Study design:** Quasi‐experimental study
	**Unit of randomization:** Not randomised
	**Type of study:** BEP
**Participants**	**Location/Setting:** India
	**Population:** Not specified
	**Sample size:** 20
	**Drop outs/withdrawal:** Not specified
	**Socio‐demographics**
	**Age range:** 20‐33 years
	**Occupation:**
	**Race:** Not specified
	**Education:** Not specified
	**Family income:** Less than Rs. 700 (US$100)
	**Inclusion criteria:**
	Pregnant women, belonging to a low socioeconomic group with a monthly family income of less than Rs. 700 (US$100) and who were in the last trimester of pregnancy were selected
	**Exclusion criteria:** Not specified
**Interventions**	**Intervention (sample size):**
	The supplement consisted of 50 g of sesame cake, 40 g jaggery and 10 g oil. The supplement contributed 30 g protein and 417 kCals (*n* = 533)
	**Control (sample size):**
	Routine diet (*n* = 508)
	**Concomitant interventions**: None
	**Training:** Not specified
	**Follow up:** Not specified
**Outcomes**	**Outcomes:**
	Nutritional intake
	Weight gain
	Haemoglobin level
	Birth weight
	Birth length
	Head circumference
	Arm circumference
	**Timing of outcome assessment:** Not specified
**Notes**	**Study start date:** Not specified
	**Study end date:** Not specified
	**Time period:** Not specified
	**Study country:** India
	**Study limitations:** Not specified
	**Funding source:** Not specified
	**Conflict of interest:** None

Risk of bias table

**Table:** 

Bias	Authors' judgement	Support for judgement
Random sequence generation (selection bias)	Unclear risk	
Allocation concealment (selection bias)	Unclear risk	
Blinding of participants and personnel (performance bias)	Unclear risk	
Blinding of outcome assessment (detection bias)	Unclear risk	
Incomplete outcome data (attrition bias)	Unclear risk	
Selective reporting (reporting bias)	Unclear risk	
Other bias	Unclear risk	
Was the allocation sequence adequately generated?	High risk	**Comment**: Quasi‐experimental trial
Was the allocation adequately concealed?	High risk	**Comment**: Quasi‐experimental trial
Were baseline outcome measurements similar?	Unclear risk	**Comment**: Insufficient information to permit judgement
Were baseline characteristics similar?	Unclear risk	**Comment**: Insufficient information to permit judgement
Were incomplete outcome data adequately addressed?	Unclear risk	**Comment:** The reasons for loss of follow‐up was not described
Was knowledge of the allocated intervention adequately prevented during the study?	High risk	**Comment**: Quasi‐experimental trial
Was the study adequately protected against contamination?	High risk	**Comment**: Quasi‐experimental trial
Was the study free from selective outcome reporting?	Unclear risk	**Comment:** Study protocol was not available to permit judgment
Was the study free from other risk of bias?	Low risk	**Comment:** No potential source of bias reported

Johnson et al. (2016)

**Table:** 

**Methods**	**Study design:** Randomized controlled trial
	**Unit of randomization:** Individually randomised
	**Type of study:** Food Distribution Program
**Participants**	**Location/setting:** Farming communities of West Kiang region, Gambia
	**Population:** 15 000
	**Sample size:** 620
	**Drop outs/withdrawal:** The exclusions were due to missing birth weight data (*N* = 129)
	**Socio‐demographics**
	**Median (*SD*) age:**
	Intervention: 29.8
	Control: 30
	Age range: 18–45 years
	**Occupation:** Farming
	**Race:** Not specified
	**Education:** Not specified
	**Family income:** Not specified
	**Inclusion criteria:**
	Women confirmed as being between 10 and 20 weeks pregnant by ultrasound
	Nonpregnant women of reproductive age (18–45 years)
	**Exclusion criteria:**
	Women who were
	(i) currently pregnant (beyond 20 weeks on ultrasound assessment), (ii) currently enrolled in another MRC study, (iii) severely anaemic at booking (haemoglobin (Hb) <7 g/dl), or (iv) report the onset of menopause were excluded from entry into the trial
**Interventions**	**Intervention (sample size):**
	Protein Energy (PE) provided with 746 kcal/day of energy from protein and lipids. It was provided to pregnant women between 10‐20th week of gestation. LNS was distributed on a weekly basis to participating women. LNS were supplied in jars, with a single (daily) dose per jar. It was provided through field workers and midwives (*n* = 151)
	**Control (sample size):**
	FeFol (iron 60 mg/day and folic acid 400 μg/day) was given as a prenatal care. It was provided to pregnant women between 10‐20th week of gestation. It was provided through field workers and midwives. Supplementation commenced the following week, receiving daily tablet supplements. Both supplement types (tablets and LNS) were distributed on a weekly basis to participating women. Women were supplied with 14 tablets per week in individual bottles and advised to take two tablets per day, preferably with food (*n* = 146)
	**Concomitant interventions:** Not specified
	**Training:**
	Prior to the start of the study, two sonographers were trained in fetal biometry
	**Follow‐up:**
	Subsequent clinic visits were made at 20 and 30 weeks of gestation, and a home visit performed within 72 h of birth
**Outcomes**	**Primary outcomes:** None
	**Secondary outcomes:**
	Low birthweight
	Preterm birth
	Small for gestational age
	**Timing of outcome assessment:** At birth
**Notes**	**Study start date:** January 2010
	**Study end date:** February 2014
	**Time period:** 4 years
	**Study country:** Gambia
	**Study limitations:**
	This was a post‐hoc analysis with a sample size that was not powered on the outcomes or for the subgroup analyses. Other limitations include not having a maternal weight measure at the very end of gestation, which would have allowed the author to quantify weight gain throughout the final trimester, and not necessarily being able to generalize results to other populations
	**Funding source:**
	This trial was supported by the UK Medical Research Council (MRC) (MC‐A760‐5QX00) and the UK Department for International Development (DFID) under the MRC/DFID Concordat agreement. WJ and SEM are funded by the UK MRC programme MC_UP_1005/1
	**Conflict of interest:** None

Risk of bias table

**Table:** 

Bias	Authors' judgement	Support for judgement
Random sequence generation (selection bias)	Low risk	**Quote:** “Randomization into the trial is in blocks of 8, using an automated system, with the 8 groups reflecting the 8 combinations of prenatal and infancy supplements”
		**Comment:** Adequately done
Allocation concealment (selection bias)	Low risk	**Quote:** “Each box of supplement is then distinguished by a number between 1 and 8”
		**Comment:** Adequately done
Blinding of participants and personnel (performance bias)	High risk	**Quote:** “Since it is not be possible to blind the field assistants or the women to the supplement type (tablet vs. LNS); all other investigators however will not know to which group the women belong”
		**Comment:** Not adequately done
Blinding of outcome assessment (detection bias)	Low risk	**Quote:** “Since it is not be possible to blind the field assistants or the women to the supplement type (tablet vs. LNS); all other investigators however will not know to which group the women belong”
		**Comment:** Adequately done
Incomplete outcome data (attrition bias)	High risk	**Comment:** 159/620 × 100 = 25.64%
Selective reporting (reporting bias)	Unclear risk	**Comment:** Published protocol not found. The outcomes specified in the methodology section have been reported in the results section
Other bias	Low risk	**Comment:** No other potential sources of bias reported
Was the allocation sequence adequately generated?	Unclear risk	
Was the allocation adequately concealed?	Unclear risk	
Were baseline outcome measurements similar?	Unclear risk	
Were baseline characteristics similar?	Unclear risk	
Were incomplete outcome data adequately addressed?	Unclear risk	
Was knowledge of the allocated intervention adequately prevented during the study?	Unclear risk	
Was the study adequately protected against contamination?	Unclear risk	
Was the study free from selective outcome reporting?	Unclear risk	
Was the study free from other risk of bias?	Unclear risk	

Kaseb et al. (2002)

**Table:** 

**Methods**	**Study design:** Randomized controlled trial
	**Unit of randomization:** Individually randomised
	**Type of study:** Balanced Energy Program
**Participants**	**Location/setting:** Health care centres of Ghaemieh and Baghfayz, Iran
	**Population:** Not specified
	**Sample size:** 53
	**Drop outs/withdrawal:** Not specified
	**Socio‐demographics**
	**Mean (*SD*) age:**
	Supplemented: 26.3 (6)
	Control: 25.5 (7)
	**Occupation:** Not specified
	**Race:** Not specified
	**Education:** Not specified
	**Family income:** Not specified
	**Inclusion criteria:**
	Healthy pregnant women who were free from systemic and genetic disorders and without a history of stillbirth, premature birth, or low birth weight infants
	**Exclusion criteria:** Using medication or addicted to illicit drugs were excluded
**Interventions**	**Intervention (sample size):**
	Traditional Food Supplementation: The experimental group received traditional food supplementation everyday, supplying an extra 400 kcal and 15 g protein through the Ghaemiah health care centre. Food supplementation which included rice‐milk porridge, lentils, pottage, cheese, yogurt, eggs and milk with bread, was given five days during the week. It was provided from 4th month of pregnancy until childbirth (*n* = 28)
	**Control (sample size):** Unsupplemented (*n* = 25)
	**Concomitant interventions:** Prenatal care
	**Training:** Not specified
	**Follow‐up:**
	Mothers were weighed once a month throughout pregnancy and 24‐h recall was used to estimate food intake. Infants were followed monthly until 4 months of age
**Outcomes**	**Primary outcomes:** None
	**Secondary outcomes:** Birth weight
	**Timing of outcome assessment:** At birth
**Notes**	**Study start date:** Not specified
	**Study end date:** Not specified
	**Time period:** Not specified
	**Study country:** Iran
	**Study limitations:** Not specified
	**Funding source:** Not specified
	**Conflict of interest:** Not specified

Risk of bias table

**Table:** 

Bias	Authors' judgement	Support for judgement
Random sequence generation (selection bias)	High risk	**Quote:** “The experimental and control groups were selected sequentially from women referred to the health care centres of Ghaemieh and Baghfayz”
		**Comment:** Not adequately done
Allocation concealment (selection bias)	High risk	**Quote:** “The experimental and control groups were selected sequentially from women referred to the health care centres of Ghaemieh and Baghfayz”
		**Comment:** Not adequately done
Blinding of participants and personnel (performance bias)	Unclear risk	**Comment:** Insufficient information to permit judgement
Blinding of outcome assessment (detection bias)	Unclear risk	**Comment:** Insufficient information to permit judgement
Incomplete outcome data (attrition bias)	Unclear risk	**Comment:** Insufficient information to permit judgement
Selective reporting (reporting bias)	Unclear risk	**Comment:** Published protocol not found. The outcomes specified in the methodology section have been reported in the results section
Other bias	Low risk	**Comment:** No other potential sources of bias reported
Was the allocation sequence adequately generated?	Unclear risk	
Was the allocation adequately concealed?	Unclear risk	
Were baseline outcome measurements similar?	Unclear risk	
Were baseline characteristics similar?	Unclear risk	
Were incomplete outcome data adequately addressed?	Unclear risk	
Was knowledge of the allocated intervention adequately prevented during the study?	Unclear risk	
Was the study adequately protected against contamination?	Unclear risk	
Was the study free from selective outcome reporting?	Unclear risk	
Was the study free from other risk of bias?	Unclear risk	

Leroy et al. (2016)

**Table:** 

**Methods**	**Study design:** Cluster‐randomized controlled trial
	**Unit of randomization:** Cluster‐randomised
	**Type of study:** Food Distribution Program
**Participants**	**Location/setting:** 2 provinces in Eastern Burundi (Cankuzo and Ruyigi)
	**Population:** Not specified
	**Sample size:** 2505
	**Drop outs/withdrawal:** Not specified
	**Socio‐demographics**
	**Mean (*SD*) age/age range:**
	Total: 28.5 (6.5), Intervention: 28.7 (6.4), Control: 28.2 (6.5)
	**Occupation:** Farming, agriculture and labor
	**Race:** Not specified
	**Education:**
	Education % (At follow‐up)
	Treatment
	None/preschool: 42.9
	Primary incomplete: 52.2
	primary complete: 0.8
	Secondary education: 4.1
	Control
	None/preschool: 52.5
	Primary incomplete: 44.7
	primary complete: 1.0
	Secondary education: 1.8
	**Family income:** Not specified
	**Inclusion criteria:**
	All pregnant women (at or after the fourth month of gestation) and mothers of children aged <6 months living in these 2 provinces
	**Exclusion criteria:** Not specified
**Interventions**	**Intervention (sample size):**
	2 arms
	T24: program benefits during pregnancy and until 23.9 months of the child
	T18: program benefits received during pregnancy and until 18 months of child's age
	The food component aimed to increase household food security in terms of both quantity and quality (through a family ration containing micronutrient‐fortified foods) and maternal and child nutrition (through the individual micronutrient‐fortified food rations targeted at pregnant and lactating mothers and children from 6 to 24 months of age) Corn‐soy blend (CSB) and fortified vegetable oil were the 2 commodities provided in household and individual rations. Intervention was delivered by *tubaramure* health promoters (*n* = 1662)
	**Control (sample size):**
	Standard care. The control group did not receive any program benefits but continued to have access to the standard care provided by the Ministry of Health (*n* = 843)
	**Concomitant interventions:**
	The core program package included 3 components: the distribution of food rations, improvements in the provision and use of health services, and a behavior change communication (BCC) strategy focused on improving health, hygiene, and nutrition practices
	**Training:**
	Repeated testing was done to assess who had acquired the required skills to conduct the fieldwork
	**Follow‐up:**
	In 2012 to assess the impact on maternal and child anaemia and on maternal knowledge and practices. In 2014, anthropometric measuring was conducted in 2014
**Outcomes**	**Primary outcomes:** None
	**Secondary outcomes:**
	Anemia (pregnant women)
	Stunting
	Haemoglobin (child)
	Iron deficiency anaemia of child
	**Timing of outcome assessment:** Follow‐up
**Notes**	**Study start date:** October 2010
	**Study end date:** 2016
	**Time period:** 6 years
	**Study country:** Burundi
	**Study limitations:**
	One limitation of our study is the lack of biomarker information to determine the etiology of anaemia in this population. In addition, the process evaluation found that some aspects of the care group sessions could have been strengthened. For example, leader mothers did not always have the required technical expertise or teaching skills to adequately transfer knowledge to beneficiary mothers. In addition, many beneficiaries were not exposed to messages on complementary feeding practices because of delays in the rollout of this BCC component
	**Funding source:**
	Supported by the Office of Food for Peace, Bureau for Democracy, Conflict, and Humanitarian Assistance and the Office of Health, Infectious Diseases, and Nutrition, Bureau for Global Health, US Agency for International Development (USAID), under terms of cooperative agreement AID‐OAA‐A‐12‐00005, through the Food and Nutrition Technical Assistance III Project (FANTA), managed by FHI 360. This study also received support from the CGIAR Research Program on Agriculture for Nutritionmand Health (A4NH), led by the International Food Policy Research Institute
	**Conflict of interest:** None

Risk of bias table

**Table:** 

Bias	Authors' judgement	Support for judgement
Random sequence generation (selection bias)	Low risk	**Quote:** “Within each stratum, 4 collines were selected by using random numbers with a fixed random number seed in Stata version 11 [StataCorp (8)], for a total of 60 collines. At a public lottery event organized in Ruyigi, at which representatives from both provinces were present, the 4 collines in each stratum were each randomised to 1 of the 4 study arms”
		**Comment:** Adequately done
Allocation concealment (selection bias)	Unclear risk	**Comment:** Insufficient information to permit judgement
Blinding of participants and personnel (performance bias)	Unclear risk	**Comment:** Insufficient information to permit judgement
Blinding of outcome assessment (detection bias)	Unclear risk	**Comment:** Insufficient information to permit judgement
Incomplete outcome data (attrition bias)	Unclear risk	**Comment:** Insufficient information to permit judgement
Selective reporting (reporting bias)	High risk	**Comment:** Outcomes mentioned in methodology section and protocol are not provided in supplementary documents (NCT01072279)
Other bias	Low risk	**Comment:** No other potential sources of bias reported
Was the allocation sequence adequately generated?	Unclear risk	
Was the allocation adequately concealed?	Unclear risk	
Were baseline outcome measurements similar?	Unclear risk	
Were baseline characteristics similar?	Unclear risk	
Were incomplete outcome data adequately addressed?	Unclear risk	
Was knowledge of the allocated intervention adequately prevented during the study?	Unclear risk	
Was the study adequately protected against contamination?	Unclear risk	
Was the study free from selective outcome reporting?	Unclear risk	
Was the study free from other risk of bias?	Unclear risk	

Liu et al. (2017)

**Table:** 

**Methods**	**Study design:** Quasi‐experimental study
	**Unit of randomization:** Not specified
	**Type of study:** Obesity Prevention
**Participants**	**Location/setting:** Large tertiary hospital, Wuhan, China
	**Population:** Not specified
	**Sample size:** 90
	**Drop outs/withdrawal:** 11
	**Socio‐demographics**
	**Mean (*SD*) age:** Total: 26.85 (2.44), Intervention: 26.73 (2.67)
	Control: 27.11 (2.31)
	**Occupation:**
	Intervention:
	Office clerk: 25
	Technician: 4
	Freelance work: 9
	Unemployed: 7
	Control:
	Office clerk: 30
	Technician: 2
	Freelance work: 14
	Unemployed: 5
	**Race:** Not specified
	**Education:** Education level
	Intervention:
	Junior high school: 5
	Senior high school: 7
	College: 13
	Bachelor or above: 20
	Control:
	Junior high school: 3
	Senior high school: 4
	College: 11
	Bachelor or above: 27
	**Family income:**
	Income level (Yuan/month)
	Intervention
	1000–1999: 3
	2000–2999: 13
	3000–3999: 11
	4000–4999: 10
	≥5000: 8
	Control
	1000–1999: 2
	2000–2999: 12
	3000–3999: 12
	4000–4999: 7
	≥5000: 12
	**Inclusion criteria:**
	Primi‐pars at least 20 years of age, having a single pregnancy confirmed by ultrasound, over 20 weeks of gestation, willing to have a vaginal birth, a pre‐pregnancy BMI of 18.5–24.9 and understanding of the written Chinese language
	**Exclusion criteria:**
	(1) over 35 years of age; (2) had pregnancy complications such as cardiovascular, digestive, endocrine and reproductive system diseases; (3) had a multiple gestation; and (4) could not have a vaginal birth because of predisposing factors such as an abnormal pelvis, malposition, or uterine fibroids
**Interventions**	**Intervention (sample size):**
	Transtheoretical model (TTM) interviewing. Intervention women received three face‐to‐face interventions and three follow‐up phone calls which were developed based on the Transtheoretical Mode (TTM)
	Intervention group was also provided a Booklet of Health Management (BHM), which described the benefits and necessary of weight management, the dietary management (controlling food intake, meeting the nutrition needs during different pregnancy stages, keeping a balanced diet, preparing foods using portions from a food exchange) and included information on an exercise plan during pregnancy
	Duration of intervention was from at least 20th week of pregnancy until 42 days postpartum
	(*n* = 45)
	**Control (sample size):**
	At the first prenatal check, the investigator provided routine health education about the effects of excessive gestational weight on pregnancy outcomes and explained the pattern of ideal weekly gain and overall maternal weight gain based on participants' calculated BMI. The maternal health handbook was distributed as a medical record at the first prenatal check which recorded the weight of each prenatal visit, which was a routine prenatal care. Duration was from at least 20th week of pregnancy until 42 days postpartum. It was delivered by the investigator
	(*n* = 45)
	**Training:**
	Not specified
	**Follow‐up:**
	Participants in the intervention group were assessed to determine their readiness for change to control their gestational weight gain during each prenatal visit between 20 and 30 weeks by asking questions congruent with each stage. After 30 weeks, four phone calls were made at 32, 34, 36, and 38–41 weeks of gestation to promote and reinforce the intervention. Women were weighed at the postpartum visit at 42 days postpartum
**Outcomes**	**Primary outcomes:** None
	**Secondary outcomes:**
	Macrosomia
	Birth weight
	**Timing of outcome assessment:** 42 days postpartum
**Notes**	**Study start date:** July 2013
	**Study end date:** June 2014
	**Time period:** 11 months
	**Study country:** China
	**Study limitations:**
	This study was limited to participants at one tertiary hospital in Wuhan. The findings, however, may not be generalized to other populations in China as China is a very large country with regional cultural differences. The study period ended at 42 days postpartum which may be a limited time frame in which to promote postpartum weight management, especially because the Chinese tradition of “doing the month” is a time when food consumption for postpartum recovery is encouraged. Use of participants' self‐report of their weight to calculate BMI may have led to bias in the data. Lastly, another limitation is that data about the incidence of breast‐feeding was not collected and breastfeeding is associated with postpartum weight reduction
	**BMI pre‐pregnancy:**
	Intervention: 20.80 ± 1.58
	Control: 21.24 ± 1.69
	**Funding source:** Not specified
	**Conflict of interest:** None

Risk of bias table

**Table:** 

Bias	Authors' judgement	Support for judgement
Random sequence generation (selection bias)	Unclear risk	
Allocation concealment (selection bias)	Unclear risk	
Blinding of participants and personnel (performance bias)	Unclear risk	
Blinding of outcome assessment (detection bias)	Unclear risk	
Incomplete outcome data (attrition bias)	Unclear risk	
Selective reporting (reporting bias)	Unclear risk	
Other bias	Unclear risk	
Was the allocation sequence adequately generated?	Low risk	**Quote:** “Participants were randomly assigned into the intervention group and control group according to the sequence of enrolment and randomised numbers produced by SPSS 19.0 software”
		**Comment:** Adequately done
Was the allocation adequately concealed?	Unclear risk	**Comment:** Insufficient information to permit judgement
Were baseline outcome measurements similar?	Low risk	**Comment:** Baseline outcomes were similar across groups
Were baseline characteristics similar?	Low risk	**Comment:** Baseline characteristics were similar across groups
Were incomplete outcome data adequately addressed?	High risk	**Comment:** 11/101 × 100 = 10.9% lost to follow‐up
Was knowledge of the allocated intervention adequately prevented during the study?	Unclear risk	**Comment:** Insufficient information to permit judgement
Was the study adequately protected against contamination?	Unclear risk	**Comment:** Insufficient information to permit judgement
Was the study free from selective outcome reporting?	Unclear risk	**Comment:** Published protocol not found. The outcomes specified in the methodology section have been reported in the results section
Was the study free from other risk of bias?	Low risk	**Comment:** No other potential sources of bias reported

Mora et al. (1978b)

**Table:** 

**Methods**	**Study design:** Quasi‐experimental study
	**Unit of randomization:** Not randomised
	**Type of study:** BEP
**Participants**	**Location/setting:** Slum district of Bogota, Colombia.
	**Population:** Not specified
	**Sample size:** 443
	**Drop outs/withdrawal:** Not specified
	**Socio‐demographics**
	**Mean (*SD*) age:**
	Intervention:
	Boys 25.9 (5.6)
	Girls 25.8 (5.1)
	Control:
	Boys 25.5 (5.8)
	Girls 26.9 (5.8)
	**Occupation:** Not specified
	**Race:** Latin American
	**Education:**
	Mothers education (year of school‐mean)
	Boys:
	Supplemented: 2.9
	Unsupplemented: 2.9
	Girls:
	Supplemented: 25.8
	Unsupplemented: 26.9
	**Family income:** (in pesos)
	Boys:
	Supplemented: 1357
	Unsupplemented: 1486
	Girls:
	Supplemented: 1452
	Unsupplemented: 1372
	**Inclusion criteria:**
	Women in first‐ or second‐trimester of pregnancy. Women were selected from a poor community and had at least 50% of previous children whose weight‐for‐height < 85% of Colombian standard
	**Exclusion criteria:** Not specified
**Interventions**	**Intervention (sample size):**
	Supplement: 60 g dried skim milk, 150 g enriched bread, and 20 g vegetable oil (Total energy: 856 kcal) (*n* = 221)
	**Control (sample size):** No intervention (*n* = 222)
	**Concomitant interventions:**
	Both groups were given free medical care for mothers and children under the age of seven. This involved prenatal, postnatal obstetrical services, attendance during delivery, emergency admission to hospital, continuing paediatric services and prescribed medications
	**Trainings:** Not specified
	**Follow up:** No follow‐up
**Outcomes**	**Outcome:**
	Stillbirths
	SGA
	Birth weigt
	Preterm birth
	**Timing of outcome assessment:** 15 days of pregnancy and developmental outcomes at birth
**Notes**	**Study start date:** Not specified
	**Study end date:** Not specified
	**Time period:** Not specified
	**Study country:** Colombia
	**Study limitations:** Not specified
	**Funding source:**
	National Institutes of Child Health and Human Development Grant R01‐HD06774; the Ford Foundation Grant 740‐0348; The German Research Foundation; and the Fund for Research and Teaching, Department of Nutrition, Harvard School of Public Health. The authors acknowledge the invaluable contribution of Nelly Mora, Esther Mantilla and Marilu Fuentes in collection of the data, and also thank Vera Kardonsky for her comments on the draft of the paper
	**Conflict of interest:** Not specified

Risk of bias table

**Table:** 

Bias	Authors' judgement	Support for judgement
Random sequence generation (selection bias)	Unclear risk	
Allocation concealment (selection bias)	Unclear risk	
Blinding of participants and personnel (performance bias)	Unclear risk	
Blinding of outcome assessment (detection bias)	Unclear risk	
Incomplete outcome data (attrition bias)	Unclear risk	
Selective reporting (reporting bias)	Unclear risk	
Other bias	Unclear risk	
Was the allocation sequence adequately generated?	High risk	**Comment**: Quasi‐experimental trial
Was the allocation adequately concealed?	High risk	**Comment**: Quasi‐experimental trial
Were baseline outcome measurements similar?	Unclear risk	**Comment**: Insufficient information to permit judgement
Were baseline characteristics similar?	Unclear risk	**Comment**: Insufficient information to permit judgement
Were incomplete outcome data adequately addressed?	Unclear risk	**Comment:** The reasons for loss of follow‐up was not described
Was knowledge of the allocated intervention adequately prevented during the study?	High risk	**Comment**: Quasi‐experimental trial
Was the study adequately protected against contamination?	High risk	**Comment**: Quasi‐experimental trial
Was the study free from selective outcome reporting?	Unclear risk	**Comment:** Study protocol was not available to permit judgment
Was the study free from other risk of bias?	Low risk	**Comment:** No potential source of bias reported

Mridha et al. (2016)

**Table:** 

**Methods**	**Study design:** Cluster‐randomised controlled trial
	**Unit of randomization:** Cluster‐randomised
	**Type of study:** Food Program
**Participants**	**Location/setting:** 11 rural unions of the Badarganj and Chirirbandar subdistricts of 2 northwest districts of Bangladesh (Rangpur and Dinajpur), 340 km from Dhaka, Bangladesh
	**Population:** 279,614
	**Sample size:** 4011
	Drop outs/withdrawal:
	One maternal death during pregnancy (in the IFA group), and 93 mothers were lost to follow‐up (22 in the LNS‐PL group and 71 in the IFA group)
	**Socio‐demographics**
	**Mean (*SD*) age:**
	Intervention: 21.8 (4.9)
	Control: 22 (5)
	**Occupation:** Transportation, construction and petty trading.
	**Race:** Not specified
	**Education:**
	Years of formal education (Mean):
	LNS‐PL: 6.4
	IFA: 6.1
	**Family income:** Not specified
	**Inclusion criteria:**
	Gestational age to be 20 weeks and no plans to move out of the study area during pregnancy or the following 3 y (i.e., a permanent resident of the study area).
	**Exclusion criteria:** Pregnancy identified and registered in the CHDP program before the beginning of enrolment
**Interventions**	**Intervention (sample size):**
	(1) Comprehensive LNS group (LNS‐PL):
	women received LNS‐PL throughout pregnancy and post partum for 6 months.
	Women received LNS‐PL (LNSs for pregnant and lactating women) during pregnancy and the first 6 months postpartum. Ingredients included soybean oil, powdered milk, peanut paste, sugar, and multiple micronutrients (thiamin, riboflavin, niacin, vitamin B‐6, vitamin B‐12, vitamin D, vitamin E, zinc, copper, and selenium at twice the amount as previously used UNIMAPP formulations), LNS‐PL was produced in individual 20‐g sachets (LNS was 20 g/day 118 kcal per day).The intervention was delivered by LAMB‐CHDP community health workers
	(*n* = 1047)
	**Control (sample size):**
	(1) Control group in which women were given IFA once daily during pregnancy and once every other day for 3 months post partum period
	(2) Child‐only LNS group:
	women received 1 tablet of 60 mg Fe and 400 mg folic acid/d during pregnancy and every alternate day during the first 3 months postpartum and their children received LNS‐C from the ages of 6–24 months
	(3) Child‐only micronutrient group:
	Women received IFA daily during pregnancy and every alternate day during the first 3 months postpartum and their children received micronutrient powder from the ages of 6 to 24 months
	The three groups of women who received IFA during pregnancy were combined and compared with the “comprehensive LNS” arm for the analysis of birth outcomes. The supplements in control group were delivered by LAMB‐CHDP community health workers and village health volunteers (VHVs)
	(*n* = 2964)
	**Concomitant interventions:**
	Monthly, the CHWs and VHVs would set classes for the discussion of child health and maternal health topics
	**Trainings:**
	Anthropometrists were trained and methods were standardized at the beginning of data collection and thereafter periodically by using methods described by (WHO MGRS)
	**Follow‐up:**
	Monthly follow‐up visits by the CHW to the woman's home. Follow‐up during pregnancy included a home visit (at 35 weeks) by the home visit team to collect data on diet and birth preparedness and a subsequent safe delivery unit (SDU) visit at 36 week (wk) for anthropometry and to assess depressive symptoms and collect bio specimens by the SDU visit team
	After delivery, the study protocol required that each woman be visited within 72 h after birth. Each woman was also called at 28 weeks of gestation and every week from 36 weeks of gestation until the delivery occurred. Retrospective data on adherence to supplement use recommendations during pregnancy were collected at a later home visit at 6 week postpartum
**Outcomes**	**Primary outcomes:**
	Miscarriage
	Stillbirth
	Perinatal mortality
	**Secondary outcomes:**
	Maternal mortality
	Low birthweight
	Preterm birth
	Small‐for‐gestational age
	Birth weight
	Birth length
	Head circumference
	Stunting
	Wasting
	Underweight
	**Timing of outcome assessment:** At delivery
**Notes**	**Study start date:** October 2011
	**Study end date:** October 2015
	**Time period:** 4 years
	**Study country:** Bangladesh
	**Study limitations:**
	The disruption of LNS‐PL supply for a period of 10 weeks, compromised the ability to investigate the full potential of LNS‐PL as an intervention. Second, it was not possible to blind the women to the type of supplement provided because the supplements were very different in appearance and taste. Third, they used LMP to estimate the duration of gestation, because it was not feasible to use ultrasonography for all participants. Fourthly, they relied on the women's retrospective recollection of supplement consumption to assess adherence instead of direct observation, so the adherence data could be inaccurate. Finally, they examined effects within several different targeted subgroups, and these exploratory effect modification results need to be interpreted with caution because they examined multiple hypotheses and the study was not powered to test each potential interaction
	**Funding source:**
	US Agency for International development's Food and Nutrition Technical Assistance III Project (FANTA), managed by Family Health International 360
	**Conflict of interest:** None

Risk of bias table

**Table:** 

Bias	Authors' judgement	Support for judgement
Random sequence generation (selection bias)	Low risk	**Quote:** “For the randomisation, the study statistician at UC Davis first stratified the 64 clusters by sub district and union, and then assigned each cluster to 1 of 4 sets containing 16 clusters each”
		**Comment:** Adequately done
Allocation concealment (selection bias)	Unclear risk	**Comment:** Insufficient information to permit judgement
Blinding of participants and personnel (performance bias)	High risk	**Quote:** “It was not possible to blind the women to the type of supplement provided because the supplements were very different in appearance and taste”
		**Comment:** High risk of bias
Blinding of outcome assessment (detection bias)	Low risk	**Quote:** “None of the evaluation staff members was involved in supplement delivery”
		“Nonetheless, researchers responsible for the collection of outcome data were kept blind to study assignment”
		**Comment:** Adequately done
Incomplete outcome data (attrition bias)	High risk	**Comment:** IFA: 413/2964 × 100 = 13.9% lost to follow‐up
		LNS‐PL: 149/1047 × 100 = 14.2% lost to follow‐up
Selective reporting (reporting bias)	High risk	**Comment:** Not all outcomes specified in protocol have been reported in the results section (NCT01715038)
Other bias	Low risk	**Comment:** No other potential sources of bias reported
Was the allocation sequence adequately generated?	Unclear risk	
Was the allocation adequately concealed?	Unclear risk	
Were baseline outcome measurements similar?	Unclear risk	
Were baseline characteristics similar?	Unclear risk	
Were incomplete outcome data adequately addressed?	Unclear risk	
Was knowledge of the allocated intervention adequately prevented during the study?	Unclear risk	
Was the study adequately protected against contamination?	Unclear risk	
Was the study free from selective outcome reporting?	Unclear risk	
Was the study free from other risk of bias?	Unclear risk	

Prentice et al. (1987)

**Table:** 

**Methods**	**Study design:** Quasi‐experimental study
	**Unit of randomization:** Nonrandomized
	**Type of study:** Balanced Energy Protein
**Participants**	**Location/Setting:** Keneba, The Gambia, West Africa
	**Population:** Not specified
	**Sample size:** 385
	**Drop outs/withdrawal:** Not specified
	**Socio‐demographics**
	**Mean (*SD*) age:** Not specified
	**Occupation:** Not specified
	**Race:** Not specified
	**Education:** Not specified
	**Family income:** Not specified
	**Inclusion criteria:** All pregnant women in Keneba
	**Exclusion criteria:** Twins were excluded
**Interventions**	**Intervention (sample size):**
	Post‐supplementation: Supplementation consisted of locally formulated biscuits and tea. The biscuits were composed of 468 kcal energy, 17.4 g protein, 25.5 g fat,180 mg Calcium, riboflavin 0.23, 0 μg vitamin A, 0 mg vitamin C. The tea was composed of 78 kcal energy, 2.9 g protein, 1.6 g fat, calcium 95 μg, vitamin C 10 mg. Maximum intake of the biscuits was limited to three 65 g biscuit and 380 g tea in the dry season and four 65 g biscuits and 380 g tea in the hungry season. Supplementation was provided every morning besides Sundays and public holidays. In Ramadan, the supplementation was carried out at night. Women were enrolled into the program as soon as their pregnancy was discovered and so the average duration of supplementation was 24 weeks. The supplements in the intervention arm were delivered by villagers (*n* = 200)
	**Control (sample size):**
	Pre‐Supplementation group: All subjects had daily access to a sophisticated level of healthcare provided by a resident midwife and paediatrician. All women were provided 6 weekly follow‐up at antenatal and postnatal clinics. This care included: monitoring of vitals, fetal growth, fetal heart rate, fetal presentation, immunization against tetanus, screening urine for infection and screening blood for anaemia and malaria. All women were also provided with 47 mg ferrous sulphate and folate. Major obstetrical difficulties were referred to the hospital for delivery. The supplements were delivered by villagers (*n* = 185)
	**Concomitant interventions:**
	All healthcare provided to the control group was also provided to the intervention group as a baseline
	**Training:** Midwives were trained to assist traditional birth attendants
	**Follow‐up:**
	All women were provided 6 weekly follow‐up at antenatal and postnatal clinics. This care included: monitoring of vitals, fetal growth, fetal heart rate, fetal presentation, immunization against tetanus, screening urine for infection and screening blood for anaemia and malaria
**Outcomes**	**Primary outcomes:** None
	**Secondary outcomes:**
	Low birthweight
	Small‐for‐gestational age
	Birth weight
	**Timing of outcome assessment:** Birth weights were recorded within 24 h of birth
**Notes**	**Study start date:** 1976
	**Study end date:** 1984
	**Time period:** 8 years
	**Study country:** The Gambia
	**Study limitations:**
	The authors used retrospective controls. Also, the effect on neonatal mortality cannot be assessed directly due to small numbers
	**Funding source:** Not specified
	**Conflict of interest:** Not specified

Risk of bias table

**Table:** 

Bias	Authors' judgement	Support for judgement
Random sequence generation (selection bias)	Unclear risk	
Allocation concealment (selection bias)	Unclear risk	
Blinding of participants and personnel (performance bias)	Unclear risk	
Blinding of outcome assessment (detection bias)	Unclear risk	
Incomplete outcome data (attrition bias)	Unclear risk	
Selective reporting (reporting bias)	Unclear risk	
Other bias	Unclear risk	
Was the allocation sequence adequately generated?	High risk	**Quote:** “It was not possible to use a randomised design of supplemented vs control subjects because of the relatively small number of births in Keneba”
		**Comment:** Study design did not permit randomisation to take place
Was the allocation adequately concealed?	Unclear risk	**Comment:** Insufficient information to permit judgement
Were baseline outcome measurements similar?	Low risk	**Comment:** Baseline outcomes were similar across groups
Were baseline characteristics similar?	Low risk	**Comment:** Baseline characteristics were similar across groups
Were incomplete outcome data adequately addressed?	Low risk	**Quote:** “Birthweights were unobtainable in three cases pre‐supplement and two cases post‐supplement because of referral for hospital deliveries”
Was knowledge of the allocated intervention adequately prevented during the study?	Unclear risk	**Comment:** Insufficient information to permit judgement
Was the study adequately protected against contamination?	Low risk	**Comment:** No blinding but the outcomes are not likely to be influenced by lack of blinding
Was the study free from selective outcome reporting?	Unclear risk	**Comment:** Published protocol not found. The outcomes specified in the methodology section have been reported in the results section
Was the study free from other risk of bias?	Low risk	**Comment:** No other potential sources of bias reported

Ross et al. (1985)

**Table:** 

**Methods**	**Study design:** Randomized controlled trial
	**Unit of randomization:** Individually randomized
	**Type of study:** Balanced Energy Protein
**Participants**	**Location/setting:** Kwa‐Mashu Polyclinic near Durban, South Africa
	**Population:** Not specified
	**Sample size:** 127
	**Drop outs/withdrawal:** None
	**Socio‐demographics**
	**Mean (*SD*) age:** Not specified
	**Occupation:** Not specified
	**Race:** Not specified
	**Education:** Not specified
	**Family income:** Not specified
	**Inclusion criteria:**
	Black women presenting before the 20th week of pregnancy with the expectation of a spontaneous vaginal delivery, willingness to attend the clinic daily until delivery to eat dietary supplements under supervision
	**Exclusion criteria:** Not specified
**Interventions**	**Intervention (sample size):**
	Group 3 (high bulk supplements)—mixture of beans and maize in a 1.2:1 ratio as mush with added vitamins. Protein: 36 g vegetable, 3247 kJ energy, 40 mg ascorbic acid (*n* = 31)
	Group 4 (low Bulk supplement)—a porridge containing 100 g dry skimmed milk, maize flour, vitamins and minerals. It differed from the group 3 supplement in its 36 g of animal protein and in its higher levels of several vitamins and calcium. Protein: 36 g animal, 8 g vegetable, 2927 kJ energy (*n* = 31)
	The supplement was provided from the 20th week of pregnancy until delivery (Monday through Friday)
	**Control (sample size):**
	Group I: No supplementation provided (*n* = 33)
	Group 2: Zinc supplementation provided (30–90 mg zinc gluconate daily) (*n* = 32)
	The supplement was provided from the 20th week of pregnancy until delivery (Monday through Friday)
	**Concomitant interventions:**
	All of the women in the study had routine medical care in the Kwa‐Mashu antenatal clinic
	**Trainings:** Not specified
	**Follow up:**
	The analyses performed on maternal serum at the mid‐gestational entry into the study were repeated at delivery on both maternal and cord blood sera. Body weights were recorded on newborns
**Outcomes**	**Primary outcomes:** None
	**Secondary outcomes:** Birth weight
	**Timing of outcome assessment:** At birth
**Notes**	**Study start date/year:** 1977
	**Study end date:** Not specified
	**Time period:** Not specified
	**Study country:** South Africa
	**Study limitations:**
	Women on the low bulk supplement likely consumed more energy on a 24 h basis than those in the other categories. Those on the low bulk supplement claimed that the supplement did not reduce the amount they ate at other meals while those on the high bulk supplements often found they were “overfilled” by the supplement and therefore ate a smaller than normal evening meal
	**Funding source:**
	Partially supported by the Ross Laboratories, Columbus, Ohio, the South African Sugar Association and the South African Medical Research Council
	**Conflict of interest:** Not specified

Risk of bias table

**Table:** 

Bias	Authors' judgement	Support for judgement
Random sequence generation (selection bias)	Unclear risk	**Quote:** “The women were randomly assigned to one of four groups.” and “Primigravidas were equally distributed by chance among the four groups”
		**Comment**: Insufficient information to determine level of bias
Allocation concealment (selection bias)	Unclear risk	**Quote:** “Patients in these two groups were given a number at the time of enrolment corresponding to numbered drug packets prepared at the pharmacy. The packets contained either zinc gluconate or the placebo. The key to the content of a patient's packet was held by the pharmacy until the end of the study”
		**Comment:** Adequately done for control group (no information for intervention group)
Blinding of participants and personnel (performance bias)	Unclear risk	**Comment**: Insufficient information to permit judgement
Blinding of outcome assessment (detection bias)	Unclear risk	**Comment**: Insufficient information to permit judgement
Incomplete outcome data (attrition bias)	High risk	**Quote:** “A move out of the community was, the principal reason why 10% of the women left the study before delivery”
		**Comment:** Not adequately done
Selective reporting (reporting bias)	Unclear risk	**Comment:** Published protocol not found. The outcomes specified in the methodology section have been reported in the results section
Other bias	Low risk	**Comment:** No other potential sources of bias reported
Was the allocation sequence adequately generated?	Unclear risk	
Was the allocation adequately concealed?	Unclear risk	
Were baseline outcome measurements similar?	Unclear risk	
Were baseline characteristics similar?	Unclear risk	
Were incomplete outcome data adequately addressed?	Unclear risk	
Was knowledge of the allocated intervention adequately prevented during the study?	Unclear risk	
Was the study adequately protected against contamination?	Unclear risk	
Was the study free from selective outcome reporting?	Unclear risk	
Was the study free from other risk of bias?	Unclear risk	

Tontisirin et al. (1986)

**Table:** 

**Methods**	**Study design:** Randomized controlled trial
	**Unit of randomization:** Individually randomized
	**Type of study:** Food Program
**Participants**	**Location/setting:** Maternal Child Health (MCH) centre, Rajchaburi (100 km from Bangkok),Thailand
	**Population:** Not specified
	**Sample size:** 43
	**Drop outs/withdrawal:** None
	**Socio‐demographics**
	**Mean (*SD*) age:**
	Intervention groups:
	I: 22.4 (3.0),
	II: 25.0 (4.8)
	III: 23.4 (4.5).
	**Age range:** 16‐30 years
	**Occupation:** Not specified
	**Race:** Not specified
	**Education:** Not specified
	**Family income:** Not specified
	**Inclusion criteria:** All nonsmoking women, who ranged in age from 16 to 30 year who attended the MCH centre (local hospital)
	**Exclusion criteria:** Not specified
**Interventions**	**Intervention (sample size):**
	Supplemented groups (I and II):
	Group I: received a mix of soybean, mungbean, sesame and sugar coming to, on average, 384 kcal energy, 9.1 g fat and 15 g protein
	Group II: received a mix of rice, dried shrimp, groundnut, sugar and oil coming to, on average, 348 kcal energy, 15.6 g fat and 13.1 g protein
	The supplement was instructed to be consumed as an additional snack and was initiated from the 28th (±2) week of gestation and stopped at birth
	**Control (sample size):**
	Nonsupplemented group (III):
	Group III: Did not receive any supplementation
	**Concomitant interventions:**
	Each participant's blood haematocrit was determined before delivery
	**Training:** Not specified
	**Follow‐up:**
	Subjects visited the MCH centre every 2 weeks until delivery. These visits consisted of General physical and obstetric examination, anthropometric measurement, 24‐h dietary recall and acceptability and consumption patterns of supplementary formula
**Outcomes**	**Primary outcomes:** None
	**Secondary outcomes:**
	Birth weight
	Birth length
	Head circumference
	**Timing of outcome assessment:** Every newborn was weighed without clothes within 30 min after delivery
**Notes**	**Study start date:** Not specified
	**Study end date:** Not specified
	**Time period:** Not specified
	**Study country:** Thailand
	**Study limitations:** Not specified
	**Funding source:** The project funded by the research committee of the Faculty of Medicine, Ramathibodi Hospital, Mahidol University
	**Conflict of interest:** Not specified

Risk of bias table

**Table:** 

Bias	Authors' judgement	Support for judgement
Random sequence generation (selection bias)	High risk	**Quote:** “Subjects were randomly divided into three groups, by rotation”
		**Comment:** Utilization of serial randomisation introduces a high risk of bias
Allocation concealment (selection bias)	Unclear risk	**Comment:** Insufficient information to permit judgement
Blinding of participants and personnel (performance bias)	Unclear risk	**Comment:** Insufficient information to permit judgement
Blinding of outcome assessment (detection bias)	Unclear risk	**Comment:** Insufficient information to permit judgement
Incomplete outcome data (attrition bias)	Low risk	**Comment:** 0/43 × 100 = 0% lost to follow‐up
Selective reporting (reporting bias)	Unclear risk	**Comment:** Published protocol not found. The outcomes specified in the methodology section have been reported in the results section
Other bias	Low risk	**Comment:** No other potential sources of bias reported
Was the allocation sequence adequately generated?	Unclear risk	
Was the allocation adequately concealed?	Unclear risk	
Were baseline outcome measurements similar?	Unclear risk	
Were baseline characteristics similar?	Unclear risk	
Were incomplete outcome data adequately addressed?	Unclear risk	
Was knowledge of the allocated intervention adequately prevented during the study?	Unclear risk	
Was the study adequately protected against contamination?	Unclear risk	
Was the study free from selective outcome reporting?	Unclear risk	
Was the study free from other risk of bias?	Unclear risk	

### Characteristics of excluded studies

**Table:** 

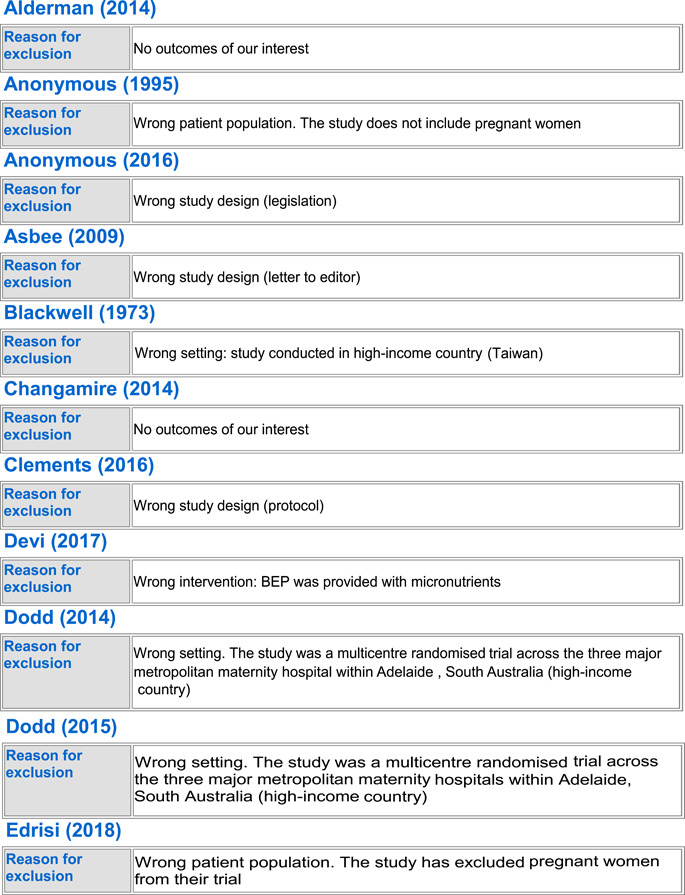
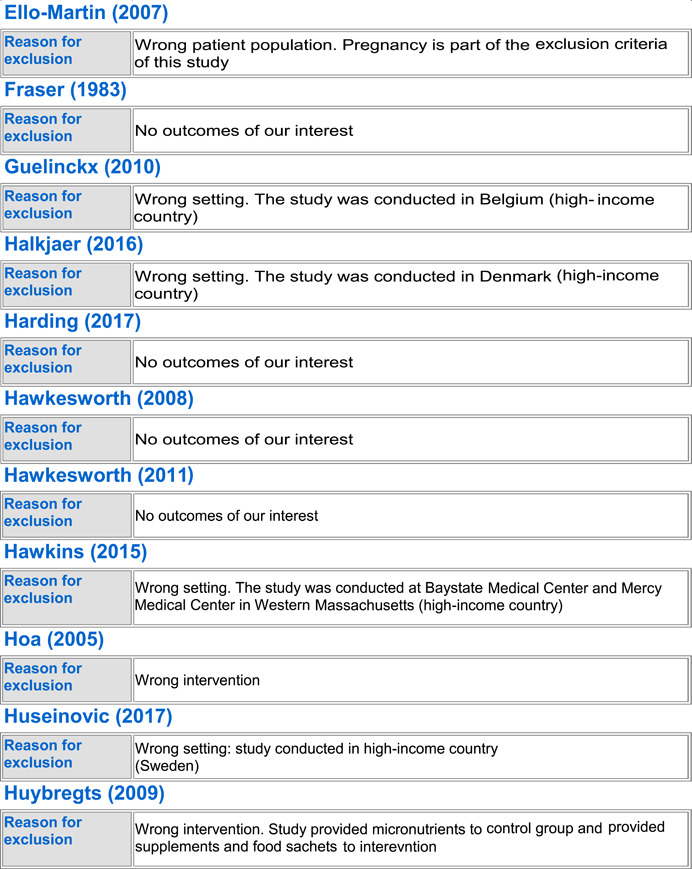
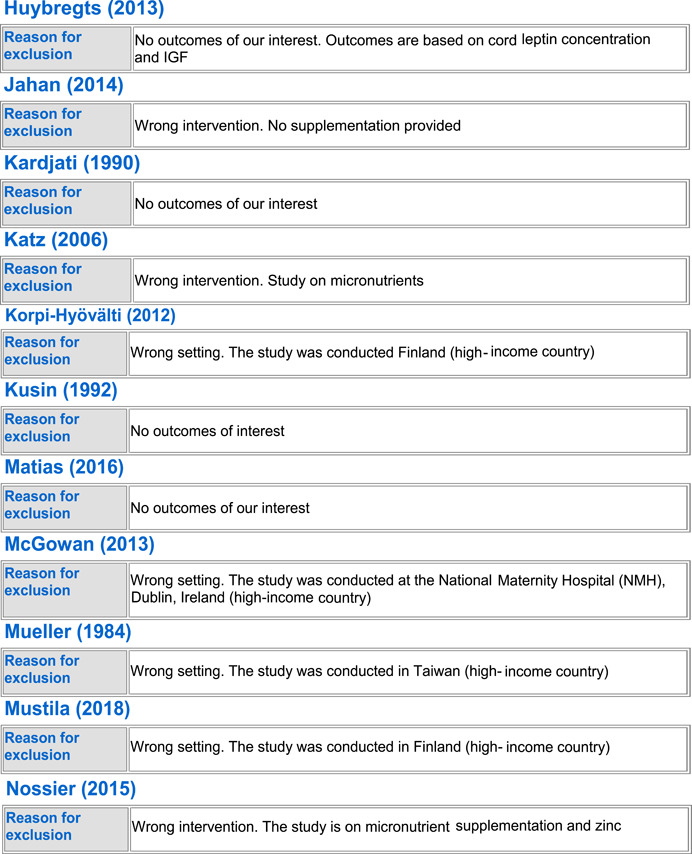
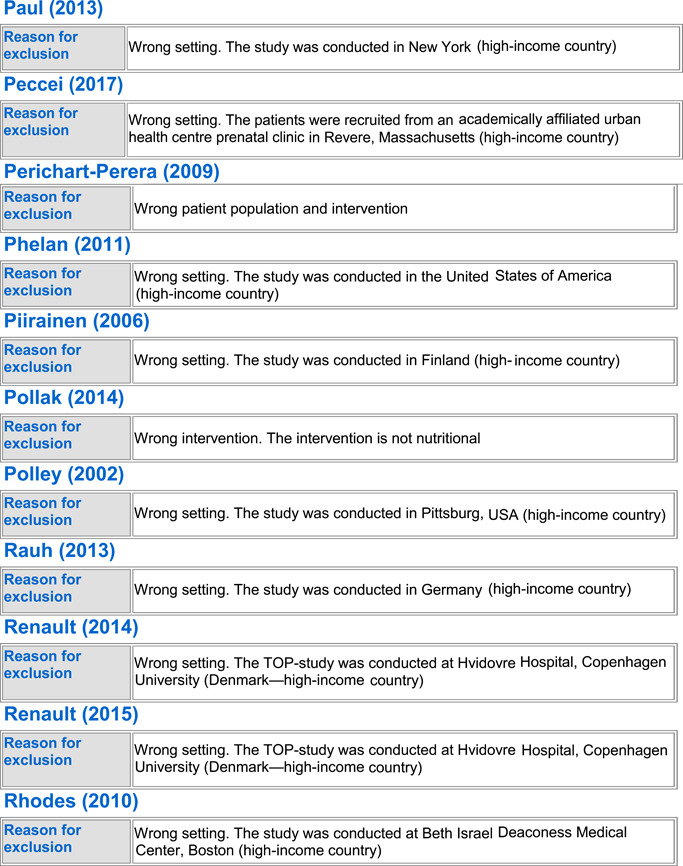
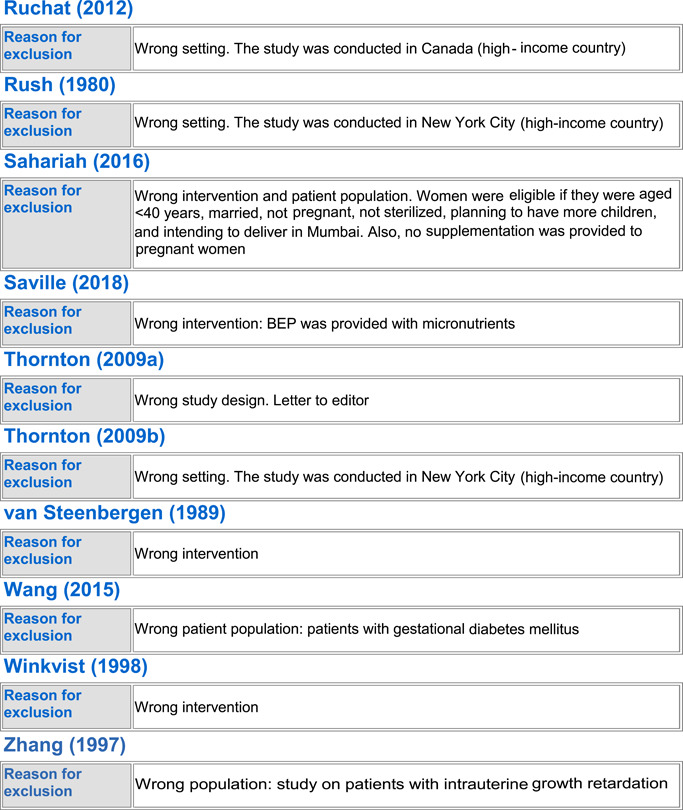

### Characteristics of studies awaiting classification

Abdel‐Aziz et al. (2018)

**Table:** 

**Methods**	**Study design:** Randomized controlled trial
	**Unit of randomization:** Individually randomised
	**Type of study:** Obesity Prevention
**Participants**	**Location/setting:** Antenatal care clinic, Center for Social and Preventive Medicine (CSPM), Pediatrics Hospital, Cairo University, Egypt
	**Population:** Not specified
	**Sample size:** 147
	**Drop outs/withdrawal:**
	Total = 31
	Lost to follow‐up = 10
	Unable to contact = 6
	Exclusions = 15 (six due to miscarriage, four due to abortion and five due to stillbirth)
	**Socio‐demographics**
	**Age range:** 20 to 30 years
	**Occupation:**
	Intervention: *n* (%)
	Housewife: 59 (73.7)
	Working: 21 (26.3)
	Control: *n* (%)
	Housewife: 51 (63.7)
	Working: 29 (36.3)
	**Race:** Food Program
	**Education:**
	Intervention: *n* (%)
	Illiterate (read and write): 24 (30.1)
	Basic education: 41 (51.2)
	Secondary and higher education: 15 (18.7)
	Control: *n* (%)
	Illiterate (read and write): 26 (32.5)
	Basic education: 33 (41.2)
	Secondary and higher education: 21 (26.3)
	**Family income:** Not specified
	**Inclusion criteria:**
	Primigravidae aged between 20 and 30 years in their first trimester (<12 weeks of gestation) of pregnancy, attending the selected “Antenatal Care” clinic, free from history of any chronic medical problems were recruited to participate
	**Exclusion criteria:**
	Women younger than 18 years (to avoid natural linear growth), having the history of previous abortion or stillbirth, presence of any chronic disease, and taking any type of medications that might interfere with the body weight (steroids, diuretics and thyroid hormones)
**Interventions**	**Intervention (sample size):**
	Received standard of care and attended six extra counselling session with a nutrition counsellor and fortnightly face‐to‐face appointments for a duration of 9 months.
	Intervention was delivered through a nutrition counsellor.
	(*n* = 75)
	**Control (sample size):**
	Participants of the control group received the standard maternity care. Women in standard care attended their regularly scheduled visits for a duration of 9 months.
	Intervention was delivered through a nutrition counsellor
	(*n* = 72)
	**Training:** Not specified
	**Follow‐up:**
	Women received standard nutrition counselling provided by the physicians and nurses based on the Maternal and child Health Program components along with prenatal care from 28 weeks of gestation till delivery
**Outcomes**	**Primary outcomes:** Not specified
	**Secondary outcomes:**
	Overweight
	Maternal anaemia
	Preterm birth
	Macrosomia
	**Timing of outcome assessment:** Not specified
**Notes**	**Study start date:** July 2015
	**Study end date:** April 2016
	**Time period:** 9 months
	**Study country:** Egypt
	**Study limitations:**
	Sizable amount of participants were illiterate, materials provided to illiterate participants were advised to get help from their educated spouse. Dropouts at end of study endorsed difficulty in follow‐up of the studied participants throughout the pregnancy until labour
	**Funding source:** Not specified
	**Conflict of interest:** Not specified

## Data and analyses

1. Balanced energy protein versus control

**Table:** 

Outcome or subgroup	Studies	Participants	Statistical method	Effect estimate
11. Miscarriage	1	24	Risk Ratio (M‐H, Random, 95% CI)	1.00 [0.07, 14.21]
12. Stillbirth	3	1913	Risk Ratio (M‐H, Random, 95% CI)	0.39 [0.19, 0.80]
13. Perinatal mortality	1	1446	Risk Ratio (M‐H, Random, 95% CI)	0.50 [0.30, 0.84]
14. Neonatal mortality	1	1446	Risk Ratio (M‐H, Random, 95% CI)	0.58 [0.32, 1.06]
15. Infant mortality	1	1446	Risk Ratio (M‐H, Random, 95% CI)	1.00 [0.59, 1.68]
16. Low birthweight	3	1830	Risk Ratio (M‐H, Random, 95% CI)	0.60 [0.41, 0.86]
17. Preterm birth	2	467	Risk Ratio (M‐H, Random, 95% CI)	0.86 [0.50, 1.46]
18. Small‐for‐gestational age	5	1844	Risk Ratio (M‐H, Random, 95% CI)	0.71 [0.54, 0.94]
19. Birth weight (g)	8	2190	Mean Difference (IV, Random, 95% CI)	107.28 [68.51, 146.04]
110. Birth length (cm)	2	67	Mean Difference (IV, Random, 95% CI)	0.28 [−0.36, 0.92]
111. Head circumference (cm)	1	71	Mean Difference (IV, Random, 95% CI)	0.54 [−0.20, 1.29]

2. Balanced energy protein versus control (subgroup: nutritional status)

**Table:** 

Outcome or subgroup	Studies	Participants	Statistical method	Effect estimate
**21.** Stillbirth	3	1913	Risk Ratio (M‐H, Random, 95% CI)	0.39 [0.19, 0.80]
**211.** Well nourished	1	1446	Risk Ratio (M‐H, Random, 95% CI)	0.45 [0.20, 1.04]
**212.** Under nourished	2	467	Risk Ratio (M‐H, Random, 95% CI)	0.27 [0.07, 1.05]

3. Balanced energy protein versus control (subgroup: region)

**Table:** 

Outcome or subgroup	Studies	Participants	Statistical method	Effect estimate
**31.** Stillbirth	3	1913	Risk Ratio (M‐H, Random, 95% CI)	0.39 [0.19, 0.80]
**311.** Africa	1	1446	Risk Ratio (M‐H, Random, 95% CI)	0.45 [0.20, 1.04]
**312.** Asia	1	24	Risk Ratio (M‐H, Random, 95% CI)	0.33 [0.01, 7.45]
**313.** South America	1	443	Risk Ratio (M‐H, Random, 95% CI)	0.25 [0.05, 1.17]

4. Balanced energy protein versus control (subgroup: duration of supplementation)

**Table:** 

Outcome or subgroup	Studies	Participants	Statistical method	Effect estimate
**41.** Stillbirth	3	1913	Risk Ratio (M‐H, Random, 95% CI)	0.39 [0.19, 0.80]
**411.** Second Trimester	1	1446	Risk Ratio (M‐H, Random, 95% CI)	0.45 [0.20, 1.04]
**412.** Third Trimester	1	443	Risk Ratio (M‐H, Random, 95% CI)	0.25 [0.05, 1.17]
**413.** Whole Pregnancy	1	24	Risk Ratio (M‐H, Random, 95% CI)	0.33 [0.01, 7.45]

5. Balanced energy protein versus control (subgroup: location)

**Table:** 

Outcome or subgroup	Studies	Participants	Statistical method	Effect estimate
**51.** Stillbirth	3	1913	Risk Ratio (M‐H, Random, 95% CI)	0.39 [0.19, 0.80]
**511.** Urban	1	24	Risk Ratio (M‐H, Random, 95% CI)	0.33 [0.01, 7.45]
**512.** Rural	2	1889	Risk Ratio (M‐H, Random, 95% CI)	0.40 [0.19, 0.82]

6. Food distribution program versus control

**Table:** 

Outcome or subgroup	Studies	Participants	Statistical method	Effect estimate
**61.** Miscarriage	1	4011	Risk Ratio (M‐H, Random, 95% CI)	0.89 [0.67, 1.19]
**62.** Perinatal mortality	2	4852	Risk Ratio (M‐H, Random, 95% CI)	0.67 [0.41, 1.09]
**63.** Neonatal mortality	1	841	Risk Ratio (M‐H, Random, 95% CI)	0.46 [0.20, 1.04]
**64.** Infant mortality	1	841	Risk Ratio (M‐H, Random, 95% CI)	0.34 [0.01, 8.41]
**65.** Maternal mortality	2	4925	Risk Ratio (M‐H, Random, 95% CI)	0.41 [0.07, 2.49]
**66.** Anaemia in pregnant women	1	2101	Risk Ratio (M‐H, Random, 95% CI)	0.75 [0.67, 0.85]
**67.** Low birthweight	4	5552	Risk Ratio (M‐H, Random, 95% CI)	0.92 [0.84, 1.00]
**68.** Preterm birth	3	4608	Risk Ratio (M‐H, Random, 95% CI)	0.92 [0.78, 1.10]
**69.** Small‐for‐gestational age	3	4511	Risk Ratio (M‐H, Random, 95% CI)	0.94 [0.89, 1.00]
**610.** Birth weight (g)	3	5272	Mean Difference (IV, Random, 95% CI)	46.00 [45.10, 46.90]
**611.** Birth length (cm)	3	5272	Mean Difference (IV, Random, 95% CI)	0.20 [0.20, 0.20]
**612.** Head circumference (cm)	2	4490	Mean Difference (IV, Random, 95% CI)	0.07 [−0.22, 0.36]
**613.** Stunting (HAZ < −2)	2	4166	Risk Ratio (M‐H, Random, 95% CI)	0.82 [0.71, 0.94]
**614.** Wasting (WLZ < −2)	2	3883	Risk Ratio (M‐H, Random, 95% CI)	0.87 [0.78, 0.97]
**615.** Underweight (WAZ < −2)	2	4174	Risk Ratio (M‐H, Random, 95% CI)	0.84 [0.63, 1.13]

7. Food distribution program versus control (subgroup: region)

**Table:** 

Outcome or subgroup	Studies	Participants	Statistical method	Effect estimate
**71.** Perinatal mortality	2	4852	Risk Ratio (M‐H, Random, 95% CI)	0.67 [0.41, 1.09]
**711.** Asia	1	4011	Risk Ratio (M‐H, Random, 95% CI)	0.78 [0.47, 1.32]
**712.** Africa	1	841	Risk Ratio (M‐H, Random, 95% CI)	0.45 [0.19, 1.09]

8. Interventions for obesity prevention versus control

**Table:** 

Outcome or subgroup	Studies	Participants	Statistical method	Effect estimate
**81.** Macrosomia	1	90	Risk Ratio (M‐H, Random, 95% CI)	0.57 [0.18, 1.82]
**82.** Birth weight (g)	2	180	Mean Difference (IV, Random, 95% CI)	−195.57 [−349.46, −41.68]
**83.** Birth length (cm)	1	90	Mean Difference (IV, Random, 95% CI)	−0.36 [−1.12, 0.40]

## SOURCES OF SUPPORT

### Internal sources

- No sources of support provided

### External sources

- Funding for this review came from a grant from the Bill & Melinda Gates Foundation to the Centre for Global Child Health at The Hospital for Sick Children (Grant No. OPP1137750), USA.

## APPENDIX A

### Search Strategies

#### CENTRAL Search Strategy

**Search Date:** April 26th, 2019

**CENTRAL:**

ID Search Hits

#1 MeSH descriptor: [Pregnant Women] explode all trees 200

#2 'Gravidity' 317

#3 'pregnant women' 15506

#4 'preconception' 376

#5 'periconception' 25

#6 'child bearing' 2127

#7 'obstetrics' 28443

#8 'prepartum' 69

#9 'antepartum' 623

#10 'prenatal' 6238

#11 'perinatal' 7366

#12 'Balanced Energy Protein Supplementation' 134

#13 MeSH descriptor: [Energy Intake] explode all trees 11454

#14 'Dietary Proteins' 4208

#15 'Dietary Diversity Strategies' 178

#16 'diet methods' 23686

#17 'diet trend' 1758

#18 'diet standards' 1016

#19 'dietary diversity' 494

#20 'diet quality' 6776

#21 'dietary quality' 5147

#22 'Dietary Quality Index' 1938

#23 'Dietary Variety' 997

#24 'Dietary Diversity Score' 225

#25 'Food Variety Score' 616

#26 'Nutritional Diversity' 336

#27 'Nutrient Diversity' 111

#28 'Nutritional Functional Diversity' 70

#29 'Nutritional adequacy' 382

#30 'Nutrition adequacy' 433

#31 'Dietary Pattern' 1630

#32 'diet pattern' 1742

#33 'Nutrition pattern' 1288

#34 'Food Distribution Program' 654

#35 'food assistance' 910

#36 'food distribution 1981

#37 'nutrition assistance' 732

#38 'food program' 4692

#39 'Maternal Obesity Intervention' 659

#40 MeSH descriptor: [Obesity prevention and control] explode all trees 5059

#41 'weight gain' 12888

#42 humans 573032

#43 #1 or #2 or #3 or #4 or #5 or #6 or #7 or #8 or #9 or #10 or #11 and #12 or #13 or #14 and #15 or #16 or #17 or #18 or #19 or #20 or #21 or #22 or #23 or #24 or #25 or #26 or #27 or #28 or #29 or #30 or #31 or #31 or #32 or #33 and #34 or #35 or #36 or #37 or #38 and #39 or #40 or #41 and #42

Search Results: 92005

#### PubMed Search Strategy

**Search Date:** April 26th, 2019

Platform: NIH—National Library of Medicine

#### Population Strategy—Pregnant Women

"Pregnancy"[Mesh] OR "Gravidity"[Mesh] OR "Pregnant Women"[Mesh] OR "Prenatal Care"[Mesh] OR "Perinatal Care"[Mesh] OR "Prenatal Diagnosis"[Mesh] OR "Obstetrics"[Mesh] OR pregnan*[tiab] OR gravid*[tiab] OR gestat*[tiab] OR (pregnant women[tiab] OR pregnant woman[tiab] OR Periconception[tiab]) OR childbear*[tiab] OR maternity[tiab] OR obstetric*[tiab] OR (prepartum[tiab] OR pre partum[tiab]) OR (antepartum[tiab] OR ante partum[tiab]) OR (prenat*[tiab] OR pre nat*[tiab]) OR (antenat*[tiab] OR ante nat*[tiab]) OR (perinat*[tiab] OR peri nat*[tiab])

#### Dietary Diversity Strategies

("Diet/methods"[Mesh] OR "Diet/trends"[Mesh] OR "Diet/standards"[Mesh]) OR ("Dietary diversity" OR "diet diversity" OR "Diet quality" OR "Dietary Quality" OR "Dietary Quality Index" OR "Dietary Variety" OR "Diet variety" OR "Dietary Diversity Score" OR "Food Variety Score" OR "Nutritional Diversity" OR "Nutrient diversity" OR "Nutritional Functional Diversity" OR "Nutritional adequacy" OR "Nutrient adequacy" OR "Nutrition adequacy") OR (Dietary Pattern* OR Diet Pattern* OR Nutritional pattern* OR Nutrition pattern*)

#### Balanced Energy Protein Supplementation

("Energy Intake"[Mesh] OR "Dietary Proteins"[Mesh] OR protein[tiab] OR energy[tiab] AND supplement*)

#### Food Distribution Programs

("Food assistance"[Mesh] OR "Food assistance" OR "Food distribution" OR "Food aid" OR "Nutrition assistance" OR food program*)

#### Maternal Obesity Interventions

("Obesity/prevention and control"[Mesh] OR "Weight Gain"[Mesh]) AND "Humans"[Mesh]))

Search Results: 116

#### Embase Search Strategy

**Search Date:** April 26th, 2019

**Search platform**: Ebsco

i. ((((('pregnant woman'/exp OR 'pregnant woman' OR 'pregnancy'/exp OR 'pregnancy' OR 'gravidity'/exp OR 'gravidity' OR 'prenatal care'/exp OR 'prenatal care' OR 'perinatal care'/exp OR 'perinatal care' OR 'prenatal diagnosis'/exp OR 'prenatal diagnosis' OR 'obstetrics'/exp OR 'obstetrics' OR 'child bearing'/exp OR 'child bearing' OR 'preconception'/exp OR 'preconception' OR 'prepartum' OR 'periconception' OR 'antenatal')
ii. 'diet method' OR 'diet standard' OR 'dietary diversity'/exp OR 'dietary diversity' OR 'diet quality'/exp OR 'diet quality' OR 'diet quality index'/exp OR 'diet quality index' OR 'diet variety' OR 'dietary diversity score'/exp OR 'dietary diversity score' OR 'food variety score' OR 'nutritional functional diversity' OR 'nutritional adequacy' OR 'dietary pattern'/exp OR 'dietary pattern' OR 'diet pattern'/exp OR 'diet pattern' OR 'nutrition pattern'/exp OR 'nutrition pattern' OR 'nutritional pattern')
iii. ('energy intake'/exp OR 'energy intake') OR 'protein intake'/exp OR 'protein intake' OR 'dietary proteins'/exp OR 'dietary proteins' OR 'protein supplement')
iv. ('food assistance'/exp OR 'food assistance') OR 'food distribution'/exp OR 'food distribution' OR 'food aid' OR 'nutrition assistance' OR 'food program')
v. ('obesity prevention and control'/exp OR 'obesity prevention and control') OR 'body weight gain'/exp OR 'body weight gain')
vi. ('humans'/exp OR 'humans')
vii. i AND ii AND iii AND iv AND v AND vi

Search Results: 68,085

#### Medline Search Strategy

**Search Date:** April 28th, 2019

**Search platform**: Ovid

1. 'pregnant woman'/exp or 'pregnant woman'.mp. or 'pregnancy'/exp or 'pregnancy'.mp. or 'gravidity'/exp or 'gravidity'.mp. or 'prenatal care'/exp or 'prenatal care'.mp. or 'perinatal care'/exp or 'perinatal care'.mp. or 'prenatal diagnosis'/exp or 'prenatal diagnosis'.mp. or 'obstetrics'/exp or 'obstetrics'.mp. or 'child bearing'/exp or 'child bearing'.mp. or 'preconception'/exp or 'preconception'.mp. or 'prepartum'.mp. or 'periconception'.mp. or 'antenatal'.mp. [mp=title, abstract, original title, name of substance word, subject heading word, floating sub‐heading word, keyword heading word, organism supplementary concept word, protocol supplementary concept word, rare disease supplementary concept word, unique identifier, synonyms] ‐‐‐‐‐‐‐‐‐‐‐‐921733
2. ('diet method' or 'diet standard').mp. or 'dietary diversity'/exp or 'dietary diversity'.mp. or 'diet quality'/exp or 'diet quality'.mp. or 'diet quality index'/exp or 'diet quality index'.mp. or 'diet variety'.mp. or 'dietary diversity score'/exp or 'dietary diversity score'.mp. or 'food variety score'.mp. or 'nutritional functional diversity'.mp. or 'nutritional adequacy'.mp. or 'dietary pattern'/exp or 'dietary pattern'.mp. or 'diet pattern'/exp or 'diet pattern'.mp. or 'nutrition pattern'/exp or 'nutrition pattern'.mp. or 'nutritional pattern'.mp. [mp=title, abstract, original title, name of substance word, subject heading word, floating sub‐heading word, keyword heading word, organism supplementary concept word, protocol supplementary concept word, rare disease supplementary concept word, unique identifier, synonyms]‐‐‐‐‐‐‐‐‐‐‐‐‐6615
3. 'energy intake'/exp or 'energy intake'.mp. or 'protein intake'/exp or 'protein intake'.mp. or 'dietary proteins'/exp or 'dietary proteins'.mp. or 'protein supplement'.mp. [mp=title, abstract, original title, name of substance word, subject heading word, floating sub‐heading word, keyword heading word, organism supplementary concept word, protocol supplementary concept word, rare disease supplementary concept word, unique identifier, synonyms]‐‐‐‐‐‐‐‐‐‐‐‐‐‐‐‐‐‐79931
4. 'food assistance'/exp or 'food assistance'.mp. or 'food distribution'/exp or 'food distribution'.mp. or 'food aid'.mp. or 'nutrition assistance'.mp. or 'food program'.mp. [mp=title, abstract, original title, name of substance word, subject heading word, floating sub‐heading word, keyword heading word, organism supplementary concept word, protocol supplementary concept word, rare disease supplementary concept word, unique identifier, synonyms]‐‐‐‐‐‐‐‐‐1836
5. ((('obesity prevention.mp. and control'/exp) or 'obesity prevention.mp.) and control'.mp.) or 'body weight gain'/exp or 'body weight gain'.mp. [mp=title, abstract, original title, name of substance word, subject heading word, floating sub‐heading word, keyword heading word, organism supplementary concept word, protocol supplementary concept word, rare disease supplementary concept word, unique identifier, synonyms]‐‐‐‐‐‐11458
6. ('human' or 'humans').mp. [mp=title, abstract, original title, name of substance word, subject heading word, floating sub‐heading word, keyword heading word, organism supplementary concept word, protocol supplementary concept word, rare disease supplementary concept word, unique identifier, synonyms]‐‐‐‐18028637
7. 1 and 2 and 3 and 4 and 5 and 6‐‐‐‐‐‐‐‐‐‐‐‐‐‐‐‐0
8. 1 and 2 and 3 and 4 and 5‐‐‐‐‐‐‐‐‐‐‐‐‐‐‐‐‐‐0

#### Psych Info Search Strategy

**Search Date:** April 28th, 2019

Platform: OVID

((((('pregnant woman'/exp OR 'pregnant woman' OR 'pregnancy'/exp OR 'pregnancy' OR 'gravidity'/exp OR 'gravidity' OR 'prenatal care'/exp OR 'prenatal care' OR 'perinatal care'/exp OR 'perinatal care' OR 'prenatal diagnosis'/exp OR 'prenatal diagnosis' OR 'obstetrics'/exp OR 'obstetrics' OR 'child bearing'/exp OR 'child bearing' OR 'preconception'/exp OR 'preconception' OR 'prepartum' OR 'periconception' OR 'antenatal') AND ('diet method' OR 'diet standard' OR 'dietary diversity'/exp OR 'dietary diversity' OR 'diet quality'/exp OR 'diet quality' OR 'diet quality index'/exp OR 'diet quality index' OR 'diet variety' OR 'dietary diversity score'/exp OR 'dietary diversity score' OR 'food variety score' OR 'nutritional functional diversity' OR 'nutritional adequacy' OR 'dietary pattern'/exp OR 'dietary pattern' OR 'diet pattern'/exp OR 'diet pattern' OR 'nutrition pattern'/exp OR 'nutrition pattern' OR 'nutritional pattern') AND ('energy intake'/exp OR 'energy intake') OR 'protein intake'/exp OR 'protein intake' OR 'dietary proteins'/exp OR 'dietary proteins' OR 'protein supplement') AND ('food assistance'/exp OR 'food assistance') OR 'food distribution'/exp OR 'food distribution' OR 'food aid' OR 'nutrition assistance' OR 'food program') AND ('obesity prevention and control'/exp OR 'obesity prevention and control') OR 'body weight gain'/exp OR 'body weight gain') AND ('humans'/exp OR 'humans')

Search Results: 46

## References

[cl21150-bib-0001] Aşcı, Ö. , & Rathfisch, G. (2016). Effect of lifestyle interventions of pregnant women on their dietary habits, lifestyle behaviours, and weight gain: A randomised controlled trial. Journal of Health, Population and Nutition, 35, 7. 10.1186/s41043-016-0044-2 PMC5025976 26911204

[cl21150-bib-0002] Ashorn, P. , Alho, L. , Ashorn, U. , Cheung, Y. B. , Dewey, K. G. , Gondwe, A. , Harjunmaa, U. , Lartey, A. , Phiri, N. , Phiri, T. E. , & Vosti, S. A. (2015). Supplementation of maternal diets during pregnancy and for 6 months postpartum and infant diets thereafter with small‐quantity lipid‐based nutrient supplements does not promote child growth by 18 months of age in rural Malawi: A randomized controlled trial. The Journal of Nutrition, 145(6), 1345–1353. 25926413 10.3945/jn.114.207225

[cl21150-bib-0003] Ashorn, P. , Alho, L. , Ashorn, U. , Cheung, Y. B. , Dewey, K. G. , Harjunmaa, U. , Lartey, A. , Nkhoma, M. , Phiri, N. , Phuka, J. , & Vosti, S. A. (2014). The impact of lipid‐based nutrient supplement provision to pregnant women on newborn size in rural Malawi: A randomized controlled trial. The American Journal of Clinical Nutrition, 101(2), 387–397. 25646337 10.3945/ajcn.114.088617

[cl21150-bib-0004] Ceesay, S. M. , Prentice, A. M. , Cole, T. J. , Foord, F. , Poskitt, E. M. , Weaver, L. T. , & Whitehead, R. G. (1997). Effects on birth weight and perinatal mortality of maternal dietary supplements in rural Gambia: 5 year randomised controlled trial. BMJ, 315(7116), 786–7909. 9345173 10.1136/bmj.315.7111.786 PMC2127544

[cl21150-bib-0005] Dwarkanath, P. , Hsu, J. W. , Tang, G. J. , Anand, P. , Thomas, T. , Thomas, A. , Sheela, C. N. , Kurpad, A. V. , & Jahoor, F. (2016). Energy and protein supplementation does not affect protein and amino acid kinetics or pregnancy outcomes in underweight Indian women. The Journal of Nutrition, 146(2), 218–226. 26764317 10.3945/jn.115.218776

[cl21150-bib-0006] Frith, A. L. , Naved, R. T. , Persson, L. A. , & Frongillo, E. A. (2015). Early prenatal food supplementation ameliorates the negative association of maternal stress with birth size in a randomised trial. Maternal & Child Nutrition, 11(4), 537–549. 23556466 10.1111/mcn.12047 PMC4794629

[cl21150-bib-0007] Girija, A. , Geervani, P. , & Rao, G. N. (1984). Influence of dietary supplementation during pregnancy on lactation performance. Journal of Tropical Pediatrics, 30, 79–83. 6726837 10.1093/tropej/30.2.79

[cl21150-bib-0008] Johnson, W. , Darboe, M. K. , Sosseh, F. , Nshe, P. , Prentice, A. M. , & Moore, S. E. (2016). Association of prenatal lipid‐based nutritional supplementation with fetal growth in rural Gambia. Maternal & Child Nutrition, 13(2), e12367. 27696720 10.1111/mcn.12367 PMC5396370

[cl21150-bib-0009] Kaseb, F. , Kimiagar, M. , Ghafarpoor, M. , & Valaii, N. (2002). Effect of traditional food supplementation during pregnancy on maternal weight gain and birthweight. International Journal for Vitamin and Nutrition Research, 72(6), 389–393. 12596505 10.1024/0300-9831.72.6.389

[cl21150-bib-0010] Leroy, J. L. , Olney, D. , & Ruel, M. (2016). Tubaramure, a food‐assisted integrated health and nutrition program in Burundi, increases maternal and child hemoglobin concentrations and reduces anemia: A theory‐based cluster‐randomized controlled intervention trial. The Journal of Nutrition, 146(8), 1601–1608. 27412269 10.3945/jn.115.227462

[cl21150-bib-0011] Liu, Y. Q. , Liu, Y. , Hua, Y. , & Chen, X. L. (2017). Effect of diet and exercise intervention in Chinese pregnant women on gestational weight gain and perinatal outcomes: A quasi‐experimental study. Applied Nursing Research, 36, 50–56. 28720239 10.1016/j.apnr.2017.05.001

[cl21150-bib-0012] Mora, J. , Navarro, L. , Clement, J. , Wagner, M. , De Paredes, B. , & Herrera, M. G. (1978a). The effect of nutritional supplementation on calorie and protein intake of pregnant women. Nutrition Reports International, 17, 217–228.

[cl21150-bib-0013] Mora, J. O. , Clement, J. , Christiansen, N. , & Suescun, J. (1978b). Nutritional supplementation and the outcome of pregnancy. III. Perinatal and neonatal mortality. Obstetrical & Gynecological Survey, 34(6), 437–439.

[cl21150-bib-0014] Mora, J. O. , de Paredes, B. , Wagner, M. , & de Navarro, L. (1979). Nutritional supplementation and the outcome of pregnancy. I. Birth weight. The American Journal of Clinical Nutrition, 32(2), 455–462. 420135 10.1093/ajcn/32.2.455

[cl21150-bib-0015] Mridha, M. K. , Matias, S. L. , Chaparro, C. M. , Paul, R. R. , Hussain, S. , Vosti, S. A. , Harding, K. L. , Cummins, J. R. , Day, L. T. , Saha, S. L. , & Peerson, J. M. (2016). Lipid‐based nutrient supplements for pregnant women reduce newborn stunting in a cluster‐randomized controlled effectiveness trial in Bangladesh. The American Journal of Clinical Nutrition, 103(1), 236–249. 26607935 10.3945/ajcn.115.111336 PMC6443293

[cl21150-bib-0016] Prentice, A. M. , Cole, T. J. , Foord, F. A. , Lamb, W. H. , & Whitehead, R. G. (1987). Increased birthweight after prenatal dietary supplementation of rural African women. The American Journal of Clinical Nutrition, 46(6), 912–925. 3687824 10.1093/ajcn/46.6.912

[cl21150-bib-0017] Ross, S. M. , Nel, E. , & Naeye, R. L. (1985). Differing effects of low and high bulk maternal dietary supplements during pregnancy. Early Human Development, 10(3‐4), 295–302. 2985353 10.1016/0378-3782(85)90061-1

[cl21150-bib-0018] Tontisirin, K. , Booranasubkajorn, U. , Hongsumarn, A. , & Thewtong, D. (1986). Formulation and evaluation of supplementary foods for Thai pregnant women. American Journal of Clinical Nutrition, 43(6), 931–939. 3521254 10.1093/ajcn/43.6.931

[cl21150-bib-0019] Vuori, L. , Christiansen, N. , Clement, J. , Mora, J. O. , Wagner, M. , & Herrera (1979). Nutritional supplementation and the outcome of pregnancy. II. Visual habituation at 15 days. The American Journal of Clinical Nutrition, 32(2), 463–469. 420136 10.1093/ajcn/32.2.463

[cl21150-bib-0020] Vuori, L. , de Navarro, L. , Christiansen, N. , Mora, J. O. , & Herrera, M. G. (1980). Food supplementation of pregnant women at risk of malnutrition and their newborns' responsiveness to stimulation. Developmental Medicine & Child Neurology, 22(1), 61–71. 7358235 10.1111/j.1469-8749.1980.tb04306.x

[cl21150-bib-0021] Alderman, H. , Hawkesworth, S. , Lundberg, M. , Tasneem, A. , Mark, H. , & Moore, S. E. (2014). Supplemental feeding during pregnancy compared with maternal supplementation during lactation does not affect schooling and cognitive development through late adolescence. The American Journal of Clinical Nutrition, 99(1), 122–129. 24132979 10.3945/ajcn.113.063404 PMC3862451

[cl21150-bib-0022] Anonymous . (1995). Protein diet study. Treatment Review, 16, 7. 11362293

[cl21150-bib-0023] Anonymous (2016). Supplemental nutrition assistance program: Nutrition education and obesity prevention grant program. Final rule. Federal Register, 81(62), 18447–18456. 27039409

[cl21150-bib-0024] Asbee, S. M. , Jenkins, T. R. , Butler, J. R. , White, J. , Elliot, M. , & Rutledge, A. (2009). Preventing excessive weight gain during pregnancy through dietary and lifestyle counseling: A randomized controlled trial. Obstetrics & Gynecology, 113(2), 305–312. 19155899 10.1097/AOG.0b013e318195baef

[cl21150-bib-0025] Blackwell, R. , Chow, B. , Chinn, K. , Blackwell, B. , & Hsu, S. (1973). Prospective maternal nutrition study in Taiwan: Rationale, study design, feasibility and preliminary findings. Nutrition Reports International, 7, 517–532.

[cl21150-bib-0026] Changamire, F. T. , Mwiru, R. S. , Msamanga, G. I. , Spiegelman, D. , Urassa, W. , Hertzmark, E. , Fawzi, W. W. , & Peterson, K. E. (2014). Macronutrient and sociodemographic determinants of gestational weight gain among HIV‐negative women in Tanzania. Food and nutrition bulletin, 35(1), 43–50. 24791578 10.1177/156482651403500106

[cl21150-bib-0027] Clements, V. , Leung, K. , Khanal, S. , Raymond, J. , Maxwell, M. , & Rissel, C. (2016). Pragmatic cluster randomised trial of a free telephone‐based health coaching program to support women in managing weight gain during pregnancy: The Get Healthy in Pregnancy Trial. BMC Health Services Research, 16(1), 454. 27578294 10.1186/s12913-016-1704-z PMC5006383

[cl21150-bib-0028] Devi, S. , Mukhopadhyay, A. , Dwarkanath, P. , Thomas, T. , Crasta, J. , Thomas, A. , Sheela, C. N. , Hsu, J. W. , Tang, G. J. , Jahoor, F. , & Kurpad, A. V. (2017). Combined vitamin B‐12 and balanced protein‐energy supplementation affect homocysteine remethylation in the methionine cycle in pregnant south Indian women of low vitamin B‐12 status. Journal of Nutrition, 147(6), 1094–1103. 28446631 10.3945/jn.116.241042

[cl21150-bib-0029] Dodd, J. M. , Cramp, C. , Sui, Z. , Yelland, L. N. , Deussen, A. R. , Grivell, R. M. , Moran, L. J. , Crowther, C. A. , Turnbull, D. , McPhee, A. J. , & Wittert, G. (2014). The effects of antenatal dietary and lifestyle advice for women who are overweight or obese on maternal diet and physical activity: The LIMIT randomised trial. BMC Medicine, 12(1), 161. 25315237 10.1186/s12916-014-0161-y PMC4194375

[cl21150-bib-0030] Dodd, J. M. , Kannieappan, L. M. , Grivell, R. M. , Deussen, A. R. , Moran, L. J. , Yelland, L. N. , & Owens, J. A. (2015). Effects of an antenatal dietary intervention on maternal anthropometric measures in pregnant women with obesity. Obesity, 23(8), 1555–1562. 26175260 10.1002/oby.21145 PMC5054850

[cl21150-bib-0031] Edrisi, F. , Salehi, M. , Ahmadi, A. , Fararoei, M. , Rusta, F. , & Mahmoodianfard, S. (2018). Effects of supplementation with rice husk powder and rice bran on inflammatory factors in overweight and obese adults following an energy‐restricted diet: A randomized controlled trial. European Journal of Nutrition, 57(2), 833–843. 29063186 10.1007/s00394-017-1555-3

[cl21150-bib-0032] Ello‐Martin, J. A. , Roe, L. S. , Ledikwe, J. H. , Beach, A. M. , & Rolls, B. J. (2007). Dietary energy density in the treatment of obesity: A year‐long trial comparing 2 weight‐loss diets. American Journal of Clinical Nutrition, 85(6), 1465–1477. 17556681 10.1093/ajcn/85.6.1465 PMC2018610

[cl21150-bib-0033] Fraser, R. B. , Ford, F. A. , & Milner, R. D. G. (1983). A controlled trial of a high dietary fibre intake in pregnancy—Effects on plasma glucose and insulin levels. Diabetologica, 25(3), 238–241. 10.1007/BF00279936 6315514

[cl21150-bib-0034] Guelinckx, I. , Devlieger, R. , Mullie, P. , & Vansant, G. (2010). Effect of lifestyle intervention on dietary habits, physical activity, and gestational weight gain in obese pregnant women: A randomized controlled trial. The American Journal of Clinical Nutrition, 91(2), 373–380. 19955397 10.3945/ajcn.2009.28166

[cl21150-bib-0035] Halkjaer, S. I. , Nilas, L. , Carlsen, E. M. , Cortes, D. , Halldórsson, T. I. , Olsen, S. F. , Pedersen, A. E. , Krogfelt, K. A. , & Petersen, A. M. (2016). Effects of probiotics (Vivomixx®) in obese pregnant women and their newborn: Study protocol for a randomized controlled trial. Trials, 17(1), 491. 27724923 10.1186/s13063-016-1617-5 PMC5057415

[cl21150-bib-0036] Harding, K. L. , Matias, S. L. , Mridha, M. K. , Vosti, S. A. , Hussain, S. , Dewey, K. G. , & Stewart (2017). Eating down or simply eating less? The diet and health implications of these practices during pregnancy and postpartum in rural Bangladesh. Public Health Nutrition, 20(11), 1928–1940. 28629489 10.1017/S1368980017000672 PMC10261490

[cl21150-bib-0037] Hawkesworth, S. , Prentice, A. M. , Fulford, A. J. , & Moore, S. E. (2008). Dietary supplementation of rural Gambian women during pregnancy does not affect body composition in offspring at 11‐17 years of age. The Journal of Nutrition, 138(12), 2468–2473. 19022974 10.3945/jn.108.098665 PMC2635503

[cl21150-bib-0038] Hawkesworth, S. , Walker, C. G. , Sawo, Y. , Fulford, A. J. , Jarjou, L. M. , Goldberg, G. R. , Prentice, A. , Prentice, A. M. , & Moore, S. E. (2011). Nutritional supplementation during pregnancy and offspring cardiovascular disease risk in the Gambia. The American Journal of Clinical Nutrition, 94(6), 1853S–1860S. 21677054 10.3945/ajcn.110.000877

[cl21150-bib-0039] Hawkins, M. , Hosker, M. , Marcus, B. H. , Rosal, M. C. , Braun, B. , Stanek, E. J., III , Markenson, G. , & Chasan‐Taber, L. (2015). A pregnancy lifestyle intervention to prevent gestational diabetes risk factors in overweight Hispanic women: A feasibility randomized controlled trial. Diabetic Medicine, 32(1), 108–115. 25306925 10.1111/dme.12601

[cl21150-bib-0040] Hoa, P. T. , Khan, N. C. , van Beusekom, C. , Gross, R. , Conde, W. L. , & Khoi, H. D. (2005). Milk fortified with iron or iron supplementation to improve nutritional status of pregnant women: An intervention trial from rural Vietnam. Food and Nutrition Bulletin, 26(1), 32–38. 15810797 10.1177/156482650502600104

[cl21150-bib-0041] Huseinovic, E. , Ohlin, M. , Winkvist, A. , Bertz, F. , Sonesson, U. , & Brekke, H. K. (2017). Does diet intervention in line with nutrition recommendations affect dietary carbon footprint? Results from a weight loss trial among lactating women. European Journal of Clinical Nutrition, 71(10), 1241–1245. 28488687 10.1038/ejcn.2017.63

[cl21150-bib-0042] Huybregts, L. , Roberfroid, D. , Lanou, H. , Menten, J. , Meda, N. , Van Camp, J. , & Kolsteren, P. (2009). Prenatal food supplementation fortified with multiple micronutrients increases birth length: A randomized controlled trial in rural Burkina Faso. American Journal of Clinical Nutrition, 90, 1593–1600. 19812173 10.3945/ajcn.2009.28253

[cl21150-bib-0043] Huybregts, L. , Roberfroid, D. , Lanou, H. , Meda, N. , Taes, Y. , Valea, I. , D'Alessandro, U. , Kolsteren, P. , & Van Camp, J. (2013). Prenatal lipid‐based nutrient supplements increase cord leptin concentration in pregnant women from rural Burkina Faso. The Journal of Nutrition, 143(5), 576–583. 23535609 10.3945/jn.112.171181

[cl21150-bib-0044] Jahan, K. , Roy, S. K. , Mihrshahi, S. , Sultana, N. , Khatoon, S. , Roy, H. , Datta, L. R. , Roy, A. , Jahan, S. , Khatun, W. , & Nahar, N. (2014). Short‐term nutrition education reduces low birthweight and improves pregnancy outcomes among urban poor women in Bangladesh. Food and nutrition bulletin, 35(4), 414–421. 25639126 10.1177/156482651403500403

[cl21150-bib-0045] Kardjati, S. R. I. , Kusin, J. A. , & De With, C. (1990). Energy supplementation in the last trimester of pregnancy in East Java, Indonesia: Effect on maternal anthropometry. BJOG: An International Journal of Obstetrics & Gynaecology, 95(8), 783–794. 10.1111/j.1471-0528.1988.tb06553.x 3048373

[cl21150-bib-0046] Katz, J. , Christian, P. , Dominici, F. , & Zeger, S. L. (2006). Treatment effects of maternal micronutrient supplementation vary by percentiles of the birth weight distribution in rural Nepal. The Journal of Nutrition, 136(5), 1389–1394. 16614435 10.1093/jn/136.5.1389

[cl21150-bib-0047] Korpi‐Hyövälti, E. , Schwab, U. , Laaksonen, D. E. , Linjama, H. , Heinonen, S. , & Niskanen, L. (2012). Effect of intensive counselling on the quality of dietary fats in pregnant women at high risk of gestational diabetes mellitus. British Journal of Nutrition, 108(5), 910–917. 22093485 10.1017/S0007114511006118

[cl21150-bib-0049] Kusin, J. A. , Kardjari, S. , Houtkooper, J. M. , & Renqvist, U. H. (1992). Energy supplementation during pregnancy and postnatal growth. The Lancet, 340(8820), 623–626. 10.1016/0140-6736(92)92168-f 1355209

[cl21150-bib-0050] Matias, S. L. , Mridha, M. K. , Paul, R. R. , Hussain, S. , Vosti, S. A. , Arnold, C. D. , & Dewey, K. G. (2016). Prenatal lipid‐based nutrient supplements affect maternal anthropometric indicators only in certain subgroups of rural Bangladeshi women. The Journal of Nutrition, 146(9), 1775–1782. 27440259 10.3945/jn.116.232181

[cl21150-bib-0051] McGowan, C. A. , Walsh, J. M. , Byrne, J. , Curran, S. , & McAuliffe, F. M. (2013). The influence of a low glycemic index dietary intervention on maternal dietary intake, glycemic index and gestational weight gain during pregnancy: A randomized controlled trial. Nutrition Journal, 12(1), 140. 24175958 10.1186/1475-2891-12-140 PMC4176103

[cl21150-bib-0052] Mueller, W. H. , & Pollitt, E. (1984). The bacon Chow study: Effects of maternal nutritional supplementation on birth measurements of children, accounting for the size of a previous (unsupplemented) child. Early Human Development, 10(1‐2), 127–136. 6499715 10.1016/0378-3782(84)90119-1

[cl21150-bib-0053] Mustila, T. , Raitanen, J. , Keskinen, P. , & Luoto, R. (2018). A pragmatic controlled trial to prevent childhood obesity within a risk group at maternity and child health‐care clinics: Results up to six years of age (the VACOPP study). BMC Pediatrics, 18(1), 89. 29486763 10.1186/s12887-018-1065-3 PMC5828437

[cl21150-bib-0054] Nossier, S. A. , Naeim, N. E. , El‐Sayed, N. A. , & Zeid, A. A. A. (2015). The effect of zinc supplementation on pregnancy outcomes: A double‐blind, randomised controlled trial, Egypt. British Journal of Nutrition, 114(2), 274–285. 26099195 10.1017/S000711451500166X

[cl21150-bib-0055] Paul, K. H. , & Olson, C. M. (2013). Moving beyond quantity of participation in process evaluation of an intervention to prevent excessive pregnancy weight gain. International Journal of Behavioral Nutrition and Physical Activity, 10(1), 23. 23406294 10.1186/1479-5868-10-23 PMC3577440

[cl21150-bib-0056] Peccei, A. , Blake‐Lamb, T. , Rahilly, D. , Hatoum, I. , & Bryant, A. (2017). Intensive prenatal nutrition counseling in a community health setting: A randomized controlled trial. Obstetrics & Gynecology, 130(2), 423–432. 28697099 10.1097/AOG.0000000000002134

[cl21150-bib-0057] Perichart‐Perera, O. , Balas‐Nakash, M. , Parra‐Covarrubias, A. , Rodriguez‐Cano, A. , Ramirez‐Torres, A. , Ortega‐González, C. , & Vadillo‐Ortega, F. (2009). A medical nutrition therapy program improves perinatal outcomes in Mexican pregnant women with gestational diabetes and type 2 diabetes mellitus. Diabetes Educator, 35(6), 1004–1013. 19696205 10.1177/0145721709343125

[cl21150-bib-0058] Phelan, S. , Phipps, M. G. , Abrams, B. , Darroch, F. , Schaffner, A. , & Wing, R. R. (2011). Randomized trial of a behavioral intervention to prevent excessive gestational weight gain: The Fit for Delivery Study. The American Journal of Clinical Nutrition, 93(4), 772–779. 21310836 10.3945/ajcn.110.005306 PMC3057546

[cl21150-bib-0059] Piirainen, T. , Isolauri, E. , Lagström, H. , & Laitinen, K. (2006). Impact of dietary counselling on nutrient intake during pregnancy: A prospective cohort study. British Journal of Nutrition, 96(6), 1095–1104. 17181885 10.1017/bjn20061952

[cl21150-bib-0060] Pollak, K. I. , Alexander, S. C. , Bennett, G. , Lyna, P. , Coffman, C. J. , Bilheimer, A. , Farrell, D. , Bodner, M. E. , Swamy, G. K. , & Østbye, T. (2014). Weight‐related SMS texts promoting appropriate pregnancy weight gain: A pilot study. Patient Education and Counseling, 97(2), 256–260. 25153313 10.1016/j.pec.2014.07.030 PMC4512734

[cl21150-bib-0061] Polley, B. A. , Wing, R. R. , & Sims, C. J. (2002). Randomized controlled trial to prevent excessive weight gain in pregnant women. International Journal of Obesity, 26(11), 1494–1502. 12439652 10.1038/sj.ijo.0802130

[cl21150-bib-0062] Rauh, K. , Gabriel, E. , Kerschbaum, E. , Schuster, T. , von Kries, R. , Amann‐Gassner, U. , & Hauner, H. (2013). Safety and efficacy of a lifestyle intervention for pregnant women to prevent excessive maternal weight gain: A cluster‐randomized controlled trial. BMC Pregnancy and Childbirth, 13(1), 151. 23865624 10.1186/1471-2393-13-151 PMC3718707

[cl21150-bib-0063] Renault, K. M. , Carlsen, E. M. , Nørgaard, K. , Nilas, L. , Pryds, O. , Secher, N. J. , Olsen, S. F. , & Halldorsson, T. I. (2015). Intake of sweets, snacks and soft drinks predicts weight gain in obese pregnant women: Detailed analysis of the results of a randomised controlled trial. PLOS One, 10(7), e0133041. 26192183 10.1371/journal.pone.0133041 PMC4507874

[cl21150-bib-0064] Renault, K. M. , Nørgaard, K. , Nilas, L. , Carlsen, E. M. , Cortes, D. , Pryds, O. , & Secher, N. J. (2014). The Treatment of Obese Pregnant Women (TOP) study: a randomized controlled trial of the effect of physical activity intervention assessed by pedometer with or without dietary intervention in obese pregnant women. American Journal of Obstetrics and Gynecology, 210(2), 134. 10.1016/j.ajog.2013.09.029 24060449

[cl21150-bib-0065] Rhodes, E. T. , Pawlak, D. B. , Takoudes, T. C. , Ebbeling, C. B. , Feldman, H. A. , Lovesky, M. M. , Cooke, E. A. , Leidig, M. M. , & Ludwig, D. S. (2010). Effects of a low‐glycemic load diet in overweight and obese pregnant women: A pilot randomized controlled trial. The American Journal of Clinical Nutrition, 92(6), 1306–1315. 20962162 10.3945/ajcn.2010.30130 PMC2980957

[cl21150-bib-0066] Ruchat, S. M. , Davenport, M. H. , Giroux, I. , Hillier, M. , Batada, A. , Sopper, M. M. , Hammond, J. M. , & Mottola, M. F. (2012). Nutrition and exercise reduce excessive weight gain in normal‐weight pregnant women. Medicine and Science in Sports and Exercise, 44(8), 1419–1426. 22453250 10.1249/MSS.0b013e31825365f1

[cl21150-bib-0067] Rush, D. , Stein, Z. , & Susser, M. (1980). Diet in pregnancy: A randomized controlled trial of nutritional supplements. March of dimes birth defects foundation, New York (USA). Medical Education Div, 16(3), 181–187. 7000197

[cl21150-bib-0068] Sahariah, S. A. , Potdar, R. D. , Gandhi, M. , Kehoe, S. H. , Brown, N. , Sane, H. , Coakley, P. J. , Marley‐Zagar, E. , Chopra, H. , Shivshankaran, D. , & Cox, V. A. (2016). A daily snack containing leafy green vegetables, fruit, and milk before and during pregnancy prevents gestational diabetes in a randomized, controlled trial in Mumbai, India. The Journal of Nutrition, 146(7), 1453S–1460S. 27281802 10.3945/jn.115.223461 PMC4926846

[cl21150-bib-0069] Saville, N. M. , Shrestha, B. P. , Style, S. , Harris‐Fry, H. , Beard, B. J. , Sen, A. , Jha, S. , Rai, A. , Paudel, V. , Sah, R. , Paudel, P. , Copas, A. , Bhandari, B. , Neupane, R. , Morrison, J. , Gram, L. , Pulkki‐Brännström, A. M. , Skordis‐Worrall, J. , Basnet, M. , … Costello, A. (2018). Impact on birth weight and child growth of Participatory Learning and Action women's groups with and without transfers of food or cash during pregnancy: Findings of the low birth weight South Asia cluster‐randomised controlled trial (LBWSAT) in Nepal. PLOS One, 9(13), e0194064. 10.1371/journal.pone.0194064 PMC5942768 29742136

[cl21150-bib-0070] Thornton, Y. S. (2009a). Preventing excessive weight gain during pregnancy through dietary and lifestyle counseling: A randomized controlled trial. Obstetrics & Gynecology, 114(1), 173. 19546789 10.1097/AOG.0b013e3181ac3aa9

[cl21150-bib-0071] Thornton, Y. S. , Smarkola, C. , Kopacz, S. M. , & Ishoof, S. B. (2009b). Perinatal outcomes in nutritionally monitored obese pregnant women: A randomized clinical trial. Journal of the National Medical Association, 101(6), 569–577. 19585925 10.1016/s0027-9684(15)30942-1

[cl21150-bib-0072] van Steenbergen, W. M. , Kusin, J. A. , Kardjati, S. , & De With, C. (1989). Energy supplementation in the last trimester of pregnancy in East Java, Indonesia: Effect on breast‐milk output. The American Journal of Clinical Nutrition, 50(2), 274–279. 2756913 10.1093/ajcn/50.2.274

[cl21150-bib-0073] Wang, H. Y. , Jiang, H. Y. , Yang, L. P. , & Zhang, M. (2015). Impacts of dietary fat changes on pregnant women with gestational diabetes mellitus: A randomized controlled study. Asia Pacific Journal of Clinical Nutrition, 24(1), 58–64. 25740743 10.6133/apjcn.2015.24.1.19

[cl21150-bib-0074] Winkvist, A. , Habicht, J. P. , & Rasmussen, K. M. (1998). Linking maternal and infant benefits of a nutritional supplement during pregnancy and lactation. The American Journal of Clinical Nutrition, 68(3), 656–661. 9734744 10.1093/ajcn/68.3.656

[cl21150-bib-0075] Zhang, L. (1997). The effects of essential fatty acids preparation in the treatment of intrauterine growth retardation. American Journal of Perinatology, 14(09), 535–537. 9394162 10.1055/s-2007-994329

[cl21150-bib-0076] Abdel‐Aziz, S. B. , Hegazy, I. S. , Mohamed, D. A. , Kasem, M. A. E. , & Hagag, S. S. (2018). Effect of dietary counselling on preventing excessive weight gain during pregnancy. Public Health, 154, 172–181. 29248827 10.1016/j.puhe.2017.10.014

[cl21150-bib-0077] Ahmed, T. , Hossain, M. , & Sanin, K. I. (2012). Global burden of maternal and child undernutrition and micronutrient deficiencies maternal and child undernutrition and micronutrient deficiencies. Annals of Nutrition and Metabolism, 61(Suppl 1), 8–17. 23343943 10.1159/000345165

[cl21150-bib-0078] Alfaradhi, M. Z. , & Ozanne, S. E. (2011). Developmental programming in response to maternal over nutrition. Frontiers in Genetics, 2, 27. 22303323 10.3389/fgene.2011.00027 PMC3268582

[cl21150-bib-0079] Allen, L. , De Benoist, B. , Dary, O. , & Hurrell, R. (2006). Guidelines on food fortification with micronutrients. Geneva: World Health Organization. https://www.who.int/nutrition/publications/guide_food_fortification_micronutrients.pdf

[cl21150-bib-0080] Baqui, A. (2008). Impact of an integrated nutrition and health programme on neonatal mortality in rural northern India. Bulletin of the World Health Organization, 86(10), 796–804. 18949217 10.2471/BLT.07.042226 PMC2649510

[cl21150-bib-0081] Bhutta, Z. A. , Das, J. K. , Rizvi, A. , Gaffey, M. F. , Walker, N. , Horton, S. , Webb, P. , Lartey, A. , & Black, R. E. (2013). Evidence‐based interventions for improvement of maternal and child nutrition: What can be done and at what cost? Lancet, 382, 452–477. 23746776 10.1016/S0140-6736(13)60996-4

[cl21150-bib-0082] Black, R. E. , Victora, C. G. , Walker, S. P. , Bhutta, Z. A. , Christian, P. , de Onis, M. , Ezzati, M. , Grantham‐McGregor, S. , Katz, J. , Martorell, R. , & Uauy, R. (2013). Maternal and child undernutrition and overweight in low‐income and middle‐income countries. Lancet, 382, 427–451. 23746772 10.1016/S0140-6736(13)60937-X

[cl21150-bib-0083] Catalano, P. , & DeMouzon, S. H. (2015). Maternal obesity and metabolic risk to the offspring: Why lifestyle interventions may have not achieved the desired outcomes. International Journal of Obesity, 39(4), 642–649. 25777180 10.1038/ijo.2015.15 PMC4700513

[cl21150-bib-0084] Centers for Disease Control and Prevention . (1998). *Recommendations to prevent and control iron deficiency in the United States*. https://www.cdc.gov/mmwr/preview/mmwrhtml/00051880.htm#:~:text=In%20these%20studies%20of%20development,than%2010.0%20g%2FdL%20but

[cl21150-bib-0085] Cheng, T. L. , & Solomon, B. S. (2014). Translating life course theory to clinical practice to address health disparities. Maternal and Child Health Journal, 18, 389–395. 23677685 10.1007/s10995-013-1279-9 PMC3883993

[cl21150-bib-0086] Christian, P. , Mullany, L. C. , Hurley, K. M. , Katz, J. , & Black, R. E. (2015). Nutrition and maternal, neonatal, and child health. Seminars in Perinatology, 39, 361–372. 26166560 10.1053/j.semperi.2015.06.009

[cl21150-bib-0087] Crispi, F. , Miranda, J. , & Gratacós, E. (2018). Long‐term cardiovascular consequences of fetal growth restriction: Biology, clinical implications, and opportunities for prevention of adult disease. American Journal of Obstetrics and Gynecology, 218, S869–S879. 29422215 10.1016/j.ajog.2017.12.012

[cl21150-bib-0088] Cochrane Effective Practice and Organisation of Care (EPOC) . *Suggested risk of bias criteria for EPOC reviews*. http://epoc.cochrane.org/resources/epoc-resources-review-authors. EPOC Resources for review authors 2017.

[cl21150-bib-0089] Dodd, J. M. , Crowther, C. A. , & Robinson, J. S. (2008). Dietary and lifestyle interventions to limit weight gain during pregnancy for obese or overweight women: A systematic review. Acta Obstetricia et Gynecologica Scandinavica, 87(7), 702–706. 18607830 10.1080/00016340802061111

[cl21150-bib-0090] Flynn, A. C. , Dalrymple, K. , Barr, S. , Poston, L. , Goff, L. M. , Rogozińska, E. , van Poppel, M. N. M. , Rayanagoudar, G. , Yeo, S. , Barakat Carballo, R. , Perales, M. , Bogaerts, A. , Cecatti, J. G. , Dodd, J. , Owens, J. , Devlieger, R. , Teede, H. , Haakstad, L. , Motahari‐Tabari, N. , … Thangaratinam, S. (2016). Dietary interventions in overweight and obese pregnant women: A systematic review of the content, delivery, and outcomes of randomized controlled trials. Nutrition Reviews, 74(5), 312–328. 27083868 10.1093/nutrit/nuw005

[cl21150-bib-0091] Furber, C. M. , McGowan, L. , Bower, P. , Kontopantelis, E. , Quenby, S. , & Lavendar, T. (2013). Antenatal interventions for reducing weight in obese women for improving pregnancy outcome. Cochrane Database of Systematic Reviews, 1, CD009334. 10.1002/14651858.CD009334.pub2 PMC11297397 23440836

[cl21150-bib-0092] Garlick, P. J. , & Reeds, P. J. (2000). Proteins. In J. S. Garrow , W. P. T. James & A. Ralph (Eds.), Human nutrition and dietetics (10th ed. (77–96). Churchill Livingstone.

[cl21150-bib-0093] Gibson, R. S. , & Hotz, C. (2018). Dietary diversification/modification strategies to enhance micronutrient content and bioavailability of diets in developing countries. British Journal of Nutrition, 85(Suppl 2), S159–S166. 10.1079/bjn2001309 11509105

[cl21150-bib-0094] Gluckman, P. D. , Hanson, M. A. , Cooper, C. , & Thornburg, K. L. (2008). Effect of in utero and early‐life conditions on adult health and disease. New England Journal of Medicine, 359(1), 61–73. 18596274 10.1056/NEJMra0708473 PMC3923653

[cl21150-bib-0095] GRADEpro GDT (2015). McMaster University (developed by Evidence Prime) [Computer program]. Hamilton, ON.

[cl21150-bib-0096] Hales, C. N. , Barker, D. J. , Clark, P. M. , Cox, L. J. , Fall, C. , Osmond, C. , & Winter, P. D. (1991). Fetal and infant growth and impaired glucose tolerance at age 4. BMJ, 303(6809), 1019–1022. 1954451 10.1136/bmj.303.6809.1019 PMC1671766

[cl21150-bib-0097] Heaver, R. (2002). The World Bank India's Tamil Nadu nutrition program lessons and issues in management and capacity development. HNP discussion paper series. Washington, DC: World Bank. https://openknowledge.worldbank.org/handle/10986/13787

[cl21150-bib-0098] Higgins, J. P. T. , Altman, D. G. , Gotzsche, P. C. , Juni, P. , Moher, D. , Oxman, A. D. , Savovic, J. , Schulz, K. F. , Weeks, L. , & Sterne, J. A. C. (2011). The Cochrane Collaboration's tool for assessing risk of bias in randomised trials. BMJ, 343, d5928. 22008217 10.1136/bmj.d5928 PMC3196245

[cl21150-bib-0099] Imdad, A. , & Bhutta, Z. A. (2011). Effect of balanced protein energy supplementation during pregnancy on birth outcomes. BMC Public Health, 11(Suppl 3), S17. 21501434 10.1186/1471-2458-11-S3-S17 PMC3231890

[cl21150-bib-0100] Imdad, A. , & Bhutta Zulfiqar, A. (2012). Maternal nutrition and birth outcomes: Effect of balanced protein‐energy supplementation. Paediatric and Perinatal Epidemiology, 26, 178–190. 22742610 10.1111/j.1365-3016.2012.01308.x

[cl21150-bib-0101] Kapil, U. , Chaturvedi, S. , & Nayar, D. (1992). National nutrition supplementation programmes. Indian Pediatrics, 29(12), 1601–1613. 1291517

[cl21150-bib-0102] Kapil, U. (2002). Integrated child development services (ICDS) scheme: A program for holistic development of children in India. The Indian Journal of Pediatrics, 69(7), 597–601. 12173700 10.1007/BF02722688

[cl21150-bib-0103] Kimani‐Murage, E. W. , Muthuri, S. K. , Oti, S. O. , Mutua, M. K. , van de Vijver, S. , & Kyobutungi, C. (2015). Evidence of a double burden of malnutrition in urban poor settings in Nairobi, Kenya. PLOS One, 10, e0129943. 26098561 10.1371/journal.pone.0129943 PMC4476587

[cl21150-bib-0104] Kramer, M. S. , & Kakuma, R. (2003). Energy and protein intake in pregnancy. Cochrane Database of Systematic Reviews, 4, CD000032. 10.1002/14651858.CD000032 14583907

[cl21150-bib-0105] Lassi, Z. S. , Imdad, A. , Ranjit, D. , Surin, G. S. S. , Salam, R. A. , & Bhutta, Z. A. (2019). PROTOCOL: Effects of nutritional interventions during pregnancy on birth, child health, and development outcomes: A systematic review of evidence from low and middle income countries. Campbell Systematic Reviews, 15(1–2), e1019. 10.1002/cl2.1019 37131465 PMC8533798

[cl21150-bib-0106] Mandy, M. , & Nyirenda, M. (2018). Developmental origins of health and disease: The relevance to developing nations. International Health, 10(2), 66–70. 29528398 10.1093/inthealth/ihy006 PMC5856182

[cl21150-bib-0107] Maršál, K. (2018). Physiological adaptation of the growth‐restricted fetus. Best Practice & Research Clinical Obstetrics & Gynaecology, 49, 37–52. 29753694 10.1016/j.bpobgyn.2018.02.006

[cl21150-bib-0108] Meehan, S. , Beck, C. R. , Mair‐Jenkins, J. , Leonardi‐Bee, J. , & Puleston, R. (2014). Maternal obesity and infant mortality: A meta‐analysis. Pediatrics, 133(5), 863–871. 24709933 10.1542/peds.2013-1480

[cl21150-bib-0109] Muktabhant, B. , Lawrie, T. A. , Lumbiganon, P. , & Laopaiboon, M. (2015). Diet or exercise, or both, for preventing excessive weight gain in pregnancy. Cochrane Database of Systematic Reviews, 6, CD007145. 10.1002/14651858.CD007145.pub3 PMC9428894 26068707

[cl21150-bib-0110] Nascimento, S. L. , Surita, F. G. , Parpinelli, M. , Siani, S. , & Pinto e Silva, J. L. (2011). The effect of an antenatal physical exercise programme on maternal/perinatal outcomes and quality of life in overweight and obese pregnant women: A randomised clinical trial. BJOG, 118(12), 1455–1463. 21895947 10.1111/j.1471-0528.2011.03084.x

[cl21150-bib-0111] Nguyen, P. H. , Kim, S. S. , Sanghvi, T. , Mahmud, Z. , Tran, L. M. , Shabnam, S. , Aktar, B. , Haque, R. , Afsana, K. , Frongillo, E. A. , Ruel, M. T. , & Menon, P. (2017). Integrating nutrition interventions into an existing maternal, neonatal, and child health program increased maternal dietary diversity, micronutrient intake, and exclusive breastfeeding practices in Bangladesh: Results of a cluster‐randomized program evaluation. Journal of Nutrition, 147(12), 2326–2337. 29021370 10.3945/jn.117.257303 PMC5697969

[cl21150-bib-0112] Ota, E. , Hori, H. , Mori, R. , Tobe‐Gai, R. , & Farrar, D. (2015). Antenatal dietary education and supplementation to increase energy and protein intake. Cochrane Database of Systematic Reviews, 6, CD000032. 10.1002/14651858.CD000032.pub3 PMC12634316 26031211

[cl21150-bib-0113] Review Manager (RevMan) . [Computer program]. Version [5.3] [Computer program]. Copenhagen: The Nordic Cochrane Centre, The Cochrane Collaboration, 2014.

[cl21150-bib-0114] Rozowski, J. , & Parodi, C. G. (2008). Implications of the nutrition transition in the nutritional status on pregnant women. (307–317). Humana Press.

[cl21150-bib-0115] Stothard, K. J. , Tennant, P. W. , Bell, R. , & Rankin, J. (2009). Maternal overweight and obesity and the risk of congenital anomalies: A systematic review and meta‐analysis. Journal of the American Medical Association, 301(6), 636–650. 19211471 10.1001/jama.2009.113

[cl21150-bib-0116] Viswanathan, M. , Siega‐Riz, A. M. , Moos, M. K. , Deierlein, A. , Mumford, S. , Knaack, J. , Thieda, P. , Lux, L. J. , & Lohr, K. N. (2008). Outcomes of maternal weight gain. Evidence Report/Technology Assessment, 168, 1–223. PMC4781425 18620471

[cl21150-bib-0117] WHO (2017). *Double burden of malnutrition*. https://www.who.int/nutrition/double-burden-malnutrition/en/

[cl21150-bib-0118] World Health Organization (2020). *Stillbirths*. https://www.who.int/maternal_child_adolescent/epidemiology/stillbirth/en/

[cl21150-bib-0119] Zerfu, T. A. , Umeta, M. , & Baye, K. (2016). Dietary diversity during pregnancy is associated with reduced risk of maternal anemia, preterm delivery, and low birth weight in a prospective cohort study in rural Ethiopia. American Journal of Clinical Nutrition, 10(6), 1482–1488. 10.3945/ajcn.115.116798 27169832

